# Supplementary material for: Brain connectivity changes underlying depression and fatigue in relapsing-remitting multiple sclerosis: A systematic review
Source: PLoS One. 2024 Mar 29;19(3):e0299634. doi: 10.1371/journal.pone.0299634 (PMC10980255; doi:10.1371/journal.pone.0299634)
Supplement: S1 Table — (PDF) [file pone.0299634.s004.pdf]

S1 Table. An overview of all studies read in full and final decision.

Author	Year	Title	Decision
A. Alshehri; O. Al-iedani; J. Arm; N. Gholizadeh; T. Billiet; R. Lea; J. Lechner-Scott; S. Ramadan	2022	Neural diffusion tensor imaging metrics correlate with clinical measures in people with relapsing-remitting MS	Final Inclusion
A. Altermatt; L. Gaetano; S. Magon; D. A. Häring; D. Tomic; J. Wuerfel; E. W. Radue; L. Kappos; T. Sprenger	2018	Clinical Correlations of Brain Lesion Location in Multiple Sclerosis: Voxel-Based Analysis of a Large Clinical Trial Dataset	Final Inclusion
A. Andravizou; V. Siokas; A. Artemiadis; C. Bakirtzis; A. M. Aloizou; N. Grigoriadis; M. H. Kosmidis; G. Nasios; L. Messinis; G. Hadjigeorgiou; E. Dardiotis; E. Peristeri	2020	Clinically reliable cognitive decline in relapsing remitting multiple sclerosis: Is it the tip of the iceberg?	no D/F
A. Bisecco; G. Caiazzo; A. d'Ambrosio; R. Sacco; S. Bonavita; R. Docimo; M. Cirillo; E. Pagani; M. Filippi; F. Esposito; G. Tedeschi; A. Gallo	2016	Fatigue in multiple sclerosis: The contribution of occult white matter damage	Final Inclusion
A. C. Vogel; H. Schmidt; S. Loud; R. McBurney; F. J. Mateen	2020	Impact of the COVID-19 pandemic on the health care of >1,000 People living with multiple sclerosis: A cross-sectional study	do not correlate MRI to D/F
A. Carotenuto; H. Wilson; B. Giordano; S. P. Caminiti; Z. Chappell; S. C. R. Williams; A. Hammers; E. Silber; P. Brex; M. Politis	2020	Impaired connectivity within neuromodulatory networks in multiple sclerosis and clinical implications	Final Inclusion
A. Conte; C. Giannì; D. Belvisi; A. Cortese; N. Petsas; M. Tartaglia; P. Cimino; E. Millefiorini; A. Berardelli; P. Pantano	2020	Deep grey matter involvement and altered sensory gating in multiple sclerosis	no D/F
A. Damasceno; B. P. Damasceno; F. Cendes	2016	Atrophy of reward-related striatal structures in fatigued MS patients is independent of physical disability	Final Inclusion
A. Damasceno; L. R. Pimentel-Silva; B. P. Damasceno; F. Cendes	2020	Cognitive trajectories in relapsing–remitting multiple sclerosis: A longitudinal 6-year study	no D/F
A. E. Williams; J. T. Vietri; G. Isherwood; A. Flor	2014	Symptoms and Association with Health Outcomes in Relapsing-Remitting Multiple Sclerosis: Results of a US Patient Survey	no MRI
A. Ernst; M. Sourty; D. Roquet; V. Noblet; D. Gounot; F. Blanc; J. De Seze; L. Manning	2016	Functional and structural cerebral changes in key brain regions after a facilitation programme for episodic future thought in relapsing-remitting multiple sclerosis patients	do not correlate MRI to D/F
A. Ernst; M. Sourty; D. Roquet; V. Noblet; D. Gounot; F. Blanc; J. de Seze; L. Manning	2018	Benefits from an autobiographical memory facilitation programme in relapsing-remitting multiple sclerosis patients: a clinical and neuroimaging study	do not correlate MRI to D/F
A. Ernst; V. Noblet; E. Denkova; F. Blanc; J. de Seze; D. Gounot; L. Manning	2015	Functional cerebral changes in multiple sclerosis patients during an autobiographical memory test	Sample size <20
A. Harel; D. Sperling; M. Petracca; A. Ntranos; I. Katz-Sand; S. Krieger; F. Lublin; Z. Wang; Y. Liu; M. Inglese	2018	Brain microstructural injury occurs in patients with RRMS despite €no evidence of disease activity'	wrong D/F test

A. J. Cruz Gomez; N. V. Campos; A. Belenguer; C. Avila; C. Forn	2013	Regional Brain Atrophy and Functional Connectivity Changes Related to Fatigue in Multiple Sclerosis	Final Inclusion
A. K. Andreasen; J. Jakobsen; L. Soerensen; H. Andersen; T. Petersen; C. R. Bjarkam; J. Ahdidan	2010	Regional brain atrophy in primary fatigued patients with multiple sclerosis	Final Inclusion
A. Kever; K. Buyukturkoglu; S. N. Levin; C. S. Riley; P. De Jager; V. M. Leavitt	2022	Associations of social network structure with cognition and amygdala volume in multiple sclerosis: An exploratory investigation	Final Inclusion
A. L. Ruiz-Rizzo; P. Bublak; S. Kluckow; K. Finke; C. Gaser; M. Schwab; D. Güllmar; H. J. Müller; O. Witte; S. Rupprecht	2022	Neural distinctiveness of fatigue and low sleep quality in multiple sclerosis	Final Inclusion
A. Lazzarotto; M. Margoni; S. Franciotta; S. Zywicki; A. Riccardi; D. Poggiali; M. Anglani; P. Gallo	2020	Selective Cerebellar Atrophy Associates with Depression and Fatigue in the Early Phases of Relapse-Onset Multiple Sclerosis	Final Inclusion
A. M. Beaudoin; F. Rheault; G. Theaud; F. Laberge; K. Whittingstall; A. Lamontagne; M. Descoteaux	2021	Modern Technology in Multi-Shell Diffusion MRI Reveals Diffuse White Matter Changes in Young Adults With Relapsing-Remitting Multiple Sclerosis	Final Inclusion
A. Papadopoulou; N. Müller-Lenke; Y. Naegelin; G. Kalt; K. Bendfeldt; P. Kuster; M. Stoecklin; A. Gass; T. Sprenger; E. W. Radue; L. Kappos; I. K. Penner	2013	Contribution of cortical and white matter lesions to cognitive impairment in multiple sclerosis	Mixed MS: Do not report actual data, only stats values for RRMS group
A. Pokryszko-Dragan; A. Banaszek; M. Nowakowska-Kotas; K. Jeżowska-Jurczyk; E. Dziadkowiak; E. Gruszka; M. Zagrajek; M. Bilińska; S. Budrewicz; M. Sąsiadek; J. Bladowska	2018	Diffusion tensor imaging findings in the multiple sclerosis patients and their relationships to various aspects of disability	Final Inclusion
A. Romanello; S. Krohn; N. von Schwanenflug; C. Chien; J. Bellmann-Strobl; K. Ruprecht; F. Paul; C. Finke	2022	Functional connectivity dynamics reflect disability and multi-domain clinical impairment in patients with relapsing-remitting multiple sclerosis	Final Inclusion
A. Saberi; A. Abdolalizadeh; E. Mohammadi; M. A. Nahayati; H. Bagheri; B. Shekarchi; J. Kargar	2021	Thalamic shape abnormalities in patients with multiple sclerosis-related fatigue	Final Inclusion
A. Salter; K. Kowalec; K. C. Fitzgerald; G. Cutter; R. A. Marrie	2020	Comorbidity is associated with disease activity in MS: Findings from the CombiRx trial	Protocol paper
Akmaz, O., Koskderelioglu, A., Eskut, N., Sahan, B., Kusbeci, T.	2022	Restless legs syndrome in multiple sclerosis is related to retinal thinning	do not correlate MRI to D/F
Arm, J., Al-iedani, O., Ribbons, K., Lea, R., Lechner-Scott, J., Ramadan, S.	2021	Biochemical Correlations with Fatigue in Multiple Sclerosis Detected by MR 2D Localized Correlated Spectroscopy	no MRI
B. A. Parmenter; J. L. Shucard; D. W. Shucard	2007	Information processing deficits in multiple sclerosis: A matter of complexity	do not correlate MRI to D/F

B. A. Parmenter; R. Zivadinov; L. Kerenyi; R. Gavett; B. Weinstock-Guttman; M. G. Dwyer; N. Garg; F. Munschauer; R. H. B. Benedict	2007	Validity of the Wisconsin card sorting and Delis-Kaplan Executive Function System (DKEFS) sorting tests in multiple sclerosis	no D/F
B. Kis; B. Rumberg; P. Berlit	2008	Clinical characteristics of patients with late-onset multiple sclerosis	Mixed MS: Three different MS types but data is not differentiated
B. Nourbakhsh; C. Azevedo; J. Nunan-Saah; A. H. Maghzi; R. Spain; D. Pelletier; E. Waubant	2016	Longitudinal associations between brain structural changes and fatigue in early MS	Mixed MS:
Barešić, M., Reihl Crnogaj, M., Zadro, I., Anić, B.	2021	Demyelinating disease (multiple sclerosis) in a patient with psoriatic arthritis treated with adalimumab: a case-based review	Treatment: DMT
Benedict, R.H.B., Pol, J., Yasin, F., Hojnacki, D., Kolb, C., Eckert, S., Tacca, B., Drake, A., Wojcik, C., Morrow, S.A., Jakimovski, D., Fuchs, T.A., Dwyer, M.G., Zivadinov, R., Weinstock-Guttman, B.	2021	Recovery of cognitive function after relapse in multiple sclerosis	Treatment: Gel treatment/no fatigue or depression
C. Bauer; T. B. Dyrby; F. Sellebjerg; K. S. Madsen; O. Svolgaard; M. Blinkenberg; H. R. Siebner; K. W. Andersen	2020	Motor fatigue is associated with asymmetric connectivity properties of the corticospinal tract in multiple sclerosis	Final Inclusion
C. E. Schwartz; B. R. Quaranto; B. C. Healy; R. H. Benedict; T. L. Vollmer	2013	Cognitive reserve and symptom experience in multiple sclerosis: a buffer to disability progression over time?	wrong D/F test
C. Fazekas; M. Khalil; C. Enzinger; F. Matzer; S. Fuchs; F. Fazekas	2013	No impact of adult attachment and temperament on clinical variability in patients with clinically isolated syndrome and early multiple sclerosis	do not correlate MRI to D/F
C. Finke; J. Schlichting; S. Papazoglou; M. Scheel; A. Freing; C. Soemmer; L. M. Pech; A. Pajkert; C. Pfüller; J. T. Wuerfel; C. J. Ploner; F. Paul; A. U. Brandt	2015	Altered basal ganglia functional connectivity in multiple sclerosis patients with fatigue	Final Inclusion
C. J. Archibald; X. C. Wei; J. N. Scott; C. J. Wallace; Y. Zhang; L. M. Metz; J. R. Mitchell	2004	Posterior fossa lesion volume and slowed information processing in multiple sclerosis	Sample size <20
C. Lebrun; C. Bensa; M. Debouverie; J. De Seze; S. Wiertlievski; B. Brochet; P. Clavelou; D. Brassat; P. Labauge; E. Roullet	2008	Unexpected multiple sclerosis: Follow-up of 30 patients with magnetic resonance imaging and clinical conversion profile	do not correlate MRI to D/F
C. Lebrun; O. H. Kantarci; A. Siva; D. Pelletier; D. T. Okuda	2018	Anomalies Characteristic of Central Nervous System Demyelination: Radiologically Isolated Syndrome	do not correlate MRI to D/F
Carandini, T., Mancini, M., Bogdan, I., Rae, C.L., Barritt, A.W., Clerico, M., Sethi, A., Harrison, N., Rashid, W., Scarpini, E., Galimberti, D., Bozzali, M., Cercignani, M.	2021	In vivo evidence of functional disconnection between brainstem monoaminergic nuclei and brain networks in multiple sclerosis	no D/F
Carotenuto, A., Valsasina, P., Preziosa, P., Mistri, D., Filippi, M., Rocca, M.A.	2022	Monoaminergic network abnormalities: A marker for multiple sclerosis-related fatigue and depression	Mixed MS: Did not separate

			between MS subgroups in analysis
Chylińska, M., Karaszewski, B., Komendziński, J., Wyszomirski, A., Sabisz, A., Halas, M., Szurowska, E.	2022	Skeletonized mean diffusivity and neuropsychological performance in relapsing-remitting multiple sclerosis	Treatment: DMT
D. A. Woo; M. J. Olek; E. M. Frohman	2006	Diagnosis and Management of Multiple Sclerosis: Case Studies	case studies
D. Iancheva; A. G. Trenova; K. Terziyski; S. Kandilarova; S. Mantarova	2018	Translational validity of PASAT and the effect of fatigue and mood in patients with relapsing remitting MS: A functional MRI study	Sample size <20
D. Iancheva; A. Trenova; S. Mantarovau; K. Terziyski	2019	Functional Magnetic Resonance Imaging Correlations Between Fatigue and Cognitive Performance in Patients With Relapsing Remitting Multiple Sclerosis	Final Inclusion
Deverdun, J., Coget, A., Ayrignac, X., Carra-Dalliere, C., Krainik, A., Metzger, A., Labauge, P., Menjot de Champfleur, N., Le Bars, E.	2021	Cerebral Vasoreactivity as an Indirect MRI Marker of White Matter Tracts Alterations in Multiple Sclerosis	no D/F
E. M. Khedr; T. Desoky; A. Gamea; M. Y. Ezzeldin; A. F. Zaki	2022	Fatigue and brain atrophy in Egyptian patients with relapsing remitting multiple sclerosis	Final Inclusion
E. Portaccio; B. Goretti; V. Zipoli; B. Nacmias; M. L. Stromillo; M. L. Bartolozzi; G. Siracusa; L. Guidi; A. Federico; S. Sorbi; N. De Stefano; M. P. Amato	2009	APOE-epsilon 4 is not associated with cognitive impairment in relapsing-remitting multiple sclerosis	no D/F
E. Pravatà; C. Zecca; C. Sestieri; M. Caulo; G. C. Riccitelli; M. A. Rocca; M. Filippi; A. Cianfoni; C. Gobbi	2016	Hyperconnectivity of the dorsolateral prefrontal cortex following mental effort in multiple sclerosis patients with cognitive fatigue	Final Inclusion
E. Szabadi	2013	Functional neuroanatomy of the central noradrenergic system	no MRI
F. Al-Hussain; N. Al-Salloum; N. Alazwary; J. Saeedi; S. Howaidi; A. Daif	2017	Depression, anxiety and stress severities in multiple sclerosis patients using injectable versus oral treatments	do not correlate MRI to D/F
F. B. Tijhuis; T. A. A. Broeders; F. A. N. Santos; M. M. Schoonheim; J. Killestein; C. E. Leurs; Q. van Geest; M. D. Steenwijk; J. J. G. Geurts; H. E. Hulst; L. Douw	2021	Dynamic functional connectivity as a neural correlate of fatigue in multiple sclerosis	Final Inclusion
F. Morgante; V. Dattola; D. Crupi; M. Russo; V. Rizzo; M. F. Ghilardi; C. Terranova; P. Girlanda; A. Quartarone	2011	Is central fatigue in multiple sclerosis a disorder of movement preparation?	Final Inclusion
F. Patti; M. P. Amato; M. Trojano; S. Bastianello; M. R. Tola; B. Goretti; L. Caniatti; E. Di Monte; P. Ferrazza; V. B. Morra; S. Lo Fermo; O. Picconi; G. Luccichenti; C. S. Grp	2009	Cognitive impairment and its relation with disease measures in mildly disabled patients with relapsing-remitting multiple sclerosis: baseline results from the Cognitive Impairment in Multiple Sclerosis (COGIMUS) study	no D/F
F. Yousuf; G. Kim; S. Tauhid; B. I. Glanz; R. Chu; S. Tummala; B. C. Healy; R. Bakshi	2016	The contribution of cortical lesions to a composite MRI scale of disease severity in multiple sclerosis	do not correlate MRI to D/F
F. Zellini; G. Niepel; C. R. Tench; C. S. Constantinescu	2009	Hypothalamic involvement assessed by T1 relaxation time in patients with relapsing-remitting multiple sclerosis	Final Inclusion

F. Zhou; H. Gong; Q. Chen; B. Wang; Y. Peng; Y. Zhuang; C. S. Zee	2016	Intrinsic Functional Plasticity of the Thalamocortical System in Minimally Disabled Patients with Relapsing-Remitting Multiple Sclerosis	Final Inclusion
F. Zhou; Y. Zhuang; H. Gong; B. Wang; X. Wang; Q. Chen; L. Wu; H. Wan	2014	Altered inter-subregion connectivity of the default mode network in relapsing remitting multiple sclerosis: A functional and structural connectivity study	Final Inclusion
Fleischer, M., Schuh, H., Bickmann, N.M., Hagenacker, T., Krüger, K., Skripuletz, T., Fiedler, M., Kleinschnitz, C., Pul, R., Skuljec, J.	2022	Anti-EBNA1 IgG titre is not associated with fatigue in multiple sclerosis patients	no MRI
G. Bonnier; A. Roche; D. Romascano; S. Simioni; D. E. Meskaldji; D. Rotzinger; Y. C. Lin; G. Menegaz; M. Schluep; R. Du Pasquier; T. J. Sumpf; J. Frahm; J. P. Thiran; G. Krueger; C. Granziera	2015	Multicontrast MRI quantification of focal inflammation and degeneration in multiple sclerosis	do not correlate MRI to D/F
G. Niepel; R. Tench Ch; P. S. Morgan; N. Evangelou; D. P. Auer; C. S. Constantinescu	2006	Deep gray matter and fatigue in MS: a T1 relaxation time study	Final Inclusion
G. O. Nygaard; K. B. Walhovd; P. Sowa; J. L. Chepkoech; A. Bjørnerud; P. Due-Tønnessen; N. I. Landrø; S. Damangir; G. Spulber; A. B. Storsve; M. K. Beyer; A. M. Fjell; E. G. Celius; H. F. Harbo	2015	Cortical thickness and surface area relate to specific symptoms in early relapsing-remitting multiple sclerosis	Final Inclusion
G. Santangelo; M. D. Corte; M. Sparaco; G. Miele; F. Garramone; M. Cropano; S. Esposito; L. Lavorgna; A. Gallo; G. Tedeschi; S. Bonavita	2021	Coping strategies in relapsing-remitting multiple sclerosis non-depressed patients and their associations with disease activity	no D/F
G. Zito; E. Luders; L. Tomasevic; D. Lupoi; A. W. Toga; P. M. Thompson; P. M. Rossini; M. M. Filippi; F. Tecchio	2014	INTER-HEMISPHERIC FUNCTIONAL CONNECTIVITY CHANGES WITH CORPUS CALLOSUM MORPHOLOGY IN MULTIPLE SCLEROSIS	do not correlate MRI to D/F
Gilio, L., Fresegna, D., Gentile, A., Guadalupi, L., Sanna, K., De Vito, F., Balletta, S., Caioli, S., Rizzo, F.R., Musella, A., Iezzi, E., Moscatelli, A., Galifi, G., Fantozzi, R., Bellantonio, P., Furlan, R., Finardi, A., Vanni, V., Dolcetti, E., Bruno, A., Buttari, F., Mandolesi, G., Centonze, D., Stampanoni Bassi, M.	2022	Preventive exercise attenuates IL-2-driven mood disorders in multiple sclerosis	no MRI
Glasner P, Sabisz A, Chylińska M, Komendziński J, Wyszomirski A, Karaszewski B.	2022	Retinal nerve fiber and ganglion cell complex layer thicknesses mirror brain atrophy in patients with relapsing-remitting multiple sclerosis	do not correlate MRI to D/F
H. D. Keklikoğlu; T. K. Yoldaş; O. Zengin; E. B. Solak; S. Keskin	2010	Cognitive impairment in patients with early relapsing-remitting multiple sclerosis	no MRI
H. Hildebrandt; H. K. Hahn; J. A. Kraus; A. Schulte-Herbrüggen; B. Schwarze; G. Schwendemann	2006	Memory performance in multiple sclerosis patients correlates with central brain atrophy	Final Inclusion
H. Hildebrandt; P. Eling	2014	A longitudinal study on fatigue, depression, and their relation to neurocognition in multiple sclerosis	Final Inclusion
H. Joly; N. Capet; L. Mondot; M. Cohen; C. Suply; S. Bresch; C. Lebrun-Frenay	2020	Thalamic atrophy correlates with dysfunctional impulsivity in multiple sclerosis	do not correlate MRI to D/F

Healy, B.C., Glanz, B.I., Swallow, E., Signorovitch, J., Hagan, K., Silva, D., Pelletier, C., Chitnis, T., Weiner, H.	2021	Confirmed disability progression provides limited predictive information regarding future disease progression in multiple sclerosis	do not correlate MRI to D/F
Høgestøl, E.A., Ghezzo, S., Nygaard, G.O., Espeseth, T., Sowa, P., Beyer, M.K., Harbo, H.F., Westlye, L.T., Hulst, H.E., Alnæs, D.	2022	Functional connectivity in multiple sclerosis modelled as connectome stability: A 5-year follow-up study	do not correlate MRI to D/F
I. Håkansson; L. Johansson; C. Dahle; M. Vrethem; J. Ernerudh	2019	Fatigue scores correlate with other self-assessment data, but not with clinical and biomarker parameters, in CIS and RRMS	Mixed MS: CI and RRMS not separated for analysis
I. Specogna; F. Casagrande; A. Lorusso; M. Catalan; A. Gorian; L. Zugna; R. Longo; M. Zorzon; M. Naccarato; G. Pizzolato; M. Ukmar; M. A. Cova	2012	Functional MRI during the execution of a motor task in patients with multiple sclerosis and fatigue	Final Inclusion
J. I. Rojas; F. Sanchez; L. Patrucco; J. Miguez; C. Besada; E. Cristiano	2017	Brain structural changes in patients in the early stages of multiple sclerosis with depression	Final Inclusion
J. R. Abbatemarco; D. Ontaneda; K. Nakamura; S. Husak; Z. N. Wang; E. Alshehri; R. A. Bermel; D. S. Conway	2020	Comorbidity effect on processing speed test and MRI measures in multiple sclerosis patients	Mixed MS: MS not classified
J. Sepulcre; J. C. Masdeu; J. Goñi; G. Arrondo; N. Vélez de Mendizábal; B. Bejarano; P. Villoslada	2009	Fatigue in multiple sclerosis is associated with the disruption of frontal and parietal pathways	Mixed MS: MS not classified
J. Wilting; H. O. Rolfsnes; H. Zimmermann; M. Behrens; V. Fleischer; F. Zipp; A. Gröger	2016	Structural correlates for fatigue in early relapsing remitting multiple sclerosis	Final Inclusion
K. C. Kern; S. M. Gold; B. Lee; M. Montag; J. Horsfall; M. F. O'Connor; N. L. Sicotte	2015	Thalamic-hippocampal-prefrontal disruption in relapsing-remitting multiple sclerosis	no D/F
K. Konstantopoulos; M. Vikelis; J. A. Seikel; D. D. Mitsikostas	2010	The existence of phonatory instability in multiple sclerosis: An acoustic and electroglottographic study	do not correlate MRI to D/F
K. Makowiecki, , Stevens, N., Cullen, C.L., Zarghami, A., Nguyen, P.T., Johnson, L., Rodger, J., Hinder, M.R., Barnett, M., Young, K.M., Taylor, B.V.	2022	Safety of low-intensity repetitive transcranial magneTic brAin stimUlation foR people living with mUltiple Sclerosis (TAURUS): study protocol for a randomised controlled trial	Protocol paper
K. Okada; S. Kakeda; M. Tahara	2020	Olfactory identification associates with cognitive function and the third ventricle width in patients with relapsing-remitting multiple sclerosis	no D/F
K. Pierzchala; M. Adamczyk-Sowa; P. Dobrakowski; K. Kubicka-Baczyk; N. Niedziela; P. Sowa	2015	Demographic characteristics of MS patients in Poland's upper Silesia region	do not correlate MRI to D/F
K. Yarraguntla; F. Bao; S. Lichtman-Mikol; S. Razmjou; C. Santiago-Martinez; N. Seraji-Bozorgzad; S. Sriwastava; E. Bernitsas	2019	Characterizing Fatigue-Related White Matter Changes in MS: A Proton Magnetic Resonance Spectroscopy Study	Final Inclusion
K. Yarraguntla; N. Seraji-Bozorgzad; S. Lichtman-Mikol; S. Razmjou; F. Bao; S. Sriwastava; C. Santiago-Martinez; O. Khan; E. Bernitsas	2018	Multiple Sclerosis Fatigue: A Longitudinal Structural MRI and Diffusion Tensor Imaging Study	Final Inclusion

Kantorová, E., Hnilicová, P., Bogner, W., Grendár, M., Grossmann, J., Kováčová, S., Hečková, E., Strasser, B., Čierny, D., Zeleňák, K., Kurča, E.	2022	Neurocognitive performance in relapsing-remitting multiple sclerosis patients is associated with metabolic abnormalities of the thalamus but not the hippocampus– GABA-edited 1H MRS study	no MRI
Khedr, E.M., Abo-Elfetoh, N., Deaf, E., Hassan, H.M., Amin, M.T., Soliman, R.K., Attia, A.A., Zarzour, A.A., Zain, M., Mohamed-Hussein, A., Hashem, M.K., Hassany, S.M., Aly, A., Shoyb, A., Saber, M.	2021	Surveillance study of acute neurological manifestations among 439 egyptian patients with COVID-19 in assiut and Aswan University Hospitals	do not correlate MRI to D/F
Kopchak, O.O., Odintsova, T.A., Pulyk, O.R.	2021	COGNITIVE FUNCTIONS IN MULTIPLE SCLEROSIS PATIENTS DEPENDING ON THE DIFFERENT RISK FACTORS PRESENCE	can't access/non-English
Koubiyr, I., Dulau-Metras, C., Deloire, M., Charré-Morin, J., Saubusse, A., Brochet, B., Ruet, A.	2022	Amygdala network reorganization mediates the theory of mind performances in multiple sclerosis	do not correlate MRI to D/F
L. De Meijer; D. Merlo; O. Skibina; E. J. Grobbee; J. Gale; J. Haartsen; P. Maruff; D. Darby; H. Butzkueven; A. Van der Walt	2018	Monitoring cognitive change in multiple sclerosis using a computerized cognitive battery	Mixed MS: Do not distinguish between MS types
L. Debernard; T. R. Melzer; S. Alla; J. Eagle; S. Van Stockum; C. Graham; J. R. Osborne; J. C. Dalrymple-Alford; D. H. Miller; D. F. Mason	2015	Deep grey matter MRI abnormalities and cognitive function in relapsing-remitting multiple sclerosis	no D/F
L. Gilio; F. Buttari; L. Pavone; E. Iezzi; G. Galifi; E. Dolcetti; F. Azzolini; A. Bruno; A. Borrelli; M. Storto; R. Furlan; A. Finardi; T. Pekmezovic; J. Drulovic; G. Mandolesi; D. Fresegna; V. Vanni; D. Centonze; M. S. Bassi	2022	Fatigue in Multiple Sclerosis Is Associated with Reduced Expression of Interleukin-10 and Worse Prospective Disease Activity	Final Inclusion
L. Hofstetter; Y. Naegelin; L. Filli; P. Kuster; S. Traud; R. Smieskova; N. Mueller-Lenke; L. Kappos; A. Gass; T. Sprenger; I. K. Penner; T. E. Nichols; H. Vrenken; F. Barkhof; C. Polman; E. W. Radue; S. J. Borgwardt; K. Bendfeldt	2014	Progression in disability and regional grey matter atrophy in relapsing-remitting multiple sclerosis	wrong D/F test
L. Locatelli; R. Zivadinov; A. Grop; M. Zorzon	2004	Frontal parenchymal atrophy measures in multiple sclerosis	do not correlate MRI to D/F
L. Passamonti; A. Cerasa; M. Liguori; M. C. Gioia; P. Valentino; R. Nistico; A. Quattrone; F. Fera	2009	Neurobiological mechanisms underlying emotional processing in relapsing-remitting multiple sclerosis	Sample size <20
L. Tomasevic; G. Zito; P. Pasqualetti; M. Filippi; D. Landi; A. Ghazaryan; D. Lupoi; C. Porcaro; F. Bagnato; P. Rossini; F. Tecchio	2013	Cortico-muscular coherence as an index of fatigue in multiple sclerosis	Final Inclusion
L. Wu; M. Huang; F. Zhou; X. Zeng; H. Gong	2020	Distributed causality in resting-state network connectivity in the acute and remitting phases of RRMS	Final Inclusion
L. Wu; Y. Zhang; F. Q. Zhou; L. Gao; L. C. He; X. J. Zeng; H. H. Gong	2016	Altered intra- and interregional synchronization in relapsing-remitting multiple sclerosis: a resting-state fMRI study	Final Inclusion

Labbe, T.P., Montalba, C., Zurita, M., Ciampi, E.L., Cruz, J.P., Vasquez, M., Uribe, S., Crossley, N., Cárcamo, C.	2021	Regional brain atrophy is related to social cognition impairment in multiple sclerosis [La atrofia cerebral regional se relaciona con el deterioro de la cognición social en sclerosis múltiple]	do not correlate MRI to D/F
M. A. Rocca; A. Meani; G. C. Riccitelli; B. Colombo; M. Rodegher; A. Falini; G. Comi; M. Filippi	2016	Abnormal adaptation over time of motor network recruitment in multiple sclerosis patients with fatigue	Final Inclusion
M. A. Rocca; M. Absinta; P. Valsasina; M. Copetti; D. Caputo; G. Comi; M. Filippi	2012	Abnormal cervical cord function contributes to fatigue in multiple sclerosis	Spinal cord
M. A. Rocca; R. Gatti; F. Agosta; P. Broglia; P. Rossi; E. Riboldi; M. Corti; G. Comi; M. Filippi	2009	Influence of task complexity during coordinated hand and foot movements in MS patients with and without fatigue. A kinematic and functional MRI study	Final Inclusion
M. A. Wojtowicz; Y. Ishigami; E. L. Mazerolle; J. D. Fisk	2014	Stability of intraindividual variability as a marker of neurologic dysfunction in relapsing remitting multiple sclerosis	Sample size <20
M. C. Bonnet; M. S. A. Deloire; E. Salort; V. Dousset; K. G. Petry; B. Brochet	2006	Evidence of cognitive compensation associated with educational level in early relapsing-remitting multiple sclerosis	do not correlate MRI to D/F
M. Calabrese; F. Rinaldi; P. Grossi; I. Mattisi; V. Bernardi; A. Favaretto; P. Perini; P. Gallo	2010	Basal ganglia and frontal/parietal cortical atrophy is associated with fatigue in relapsing-remitting multiple sclerosis	Final Inclusion
M. Cavallari; M. Palotai; B. I. Glanz; S. Egorova; J. C. Prieto; B. C. Healy; T. Chitnis; C. R. G. Guttmann	2016	Fatigue predicts disease worsening in relapsing-remitting multiple sclerosis patients	Final Inclusion
M. Codella; M. A. Rocca; B. Colombo; F. Martinelli-Boneschi; G. Comi; M. Filippi	2002	Cerebral grey matter pathology and fatigue in patients with multiple sclerosis: a preliminary study	Final Inclusion
M. Filippi; M. A. Rocca; B. Colombo; A. Falini; M. Codella; G. Scotti; G. Comi	2002	Functional magnetic resonance imaging correlates of fatigue in multiple sclerosis	Final Inclusion
M. Gschwind; M. Hardmeier; D. Van De Ville; M. I. Tomescu; I. K. Penner; Y. Naegelin; P. Fuhr; C. M. Michel; M. Seeck	2016	Fluctuations of spontaneous EEG topographies predict disease state in relapsing-remitting multiple sclerosis	do not correlate MRI to D/F
M. Huang; F. Zhou; L. Wu; B. Wang; H. Wan; F. Li; X. Zeng; H. Gong	2018	Synchronization within, and interactions between, the default mode and dorsal attention networks in relapsing-remitting multiple sclerosis	Final Inclusion
M. Inglese; S. J. Park; G. Johnson; J. S. Babb; L. Miles; H. Jaggi; J. Herbert; R. I. Grossman	2007	Deep gray matter perfusion in multiple sclerosis: Dynamic susceptibility contrast perfusion magnetic resonance imaging at 3 T	wrong D/F test
M. J. Fartaria; K. O'Brien; A. Sorega; G. Bonnier; A. Roche; P. Falkovskiy; G. Krueger; T. Kober; M. B. Cuadra; C. Granziera	2017	An Ultra-High Field Study of Cerebellar Pathology in Early Relapsing-Remitting Multiple Sclerosis Using MP2RAGE	no D/F
M. Jehna; C. Langkammer; M. Wallner-Blazek; C. Neuper; M. Loitfelder; S. Ropele; S. Fuchs; M. Khalil; A. Pluta-Fuerst; F. Fazekas; C. Enzinger	2011	Cognitively preserved MS patients demonstrate functional differences in processing neutral and emotional faces	do not correlate MRI to D/F
M. L. Polliack; Y. Barak; A. Achiron	2001	Late-onset multiple sclerosis	no MRI

M. N. Burns; E. Nawacki; M. J. Kwasny; D. Pelletier; D. C. Mohr	2014	Do positive or negative stressful events predict the development of new brain lesions in people with multiple sclerosis?	no D/F
M. P. Amato; E. Portaccio; B. Goretti; V. Zipoli; A. Iudice; D. D. Pina; G. Malentacchi; S. Sabatini; P. Annunziata; M. Falcini; M. Mazzoni; M. Mortilla; C. Fonda; N. De Stefano	2010	Relevance of cognitive deterioration in early relapsing-remitting MS: A 3-year follow-up study	no D/F
M. Pardini; L. Bonzano; G. L. Mancardi; L. Roccatagliata	2010	Frontal networks play a role in fatigue perception in multiple sclerosis	Final Inclusion
M. Pardini; L. Bonzano; M. Bergamino; G. Bommarito; P. Feraco; A. Murugavel; M. Bove; G. Brichetto; A. Uccelli; G. Mancardi; L. Roccatagliata	2015	Cingulum bundle alterations underlie subjective fatigue in multiple sclerosis	Final Inclusion
M. Russo; A. Calamuneri; A. Cacciola; L. Bonanno; A. Naro; V. Dattola; E. Sessa; M. Buccafusca; D. Milardi; P. Bramanti; R. S. Calabro; G. Anastasi; A. Quartarone	2017	Neural correlates of fatigue in multiple sclerosis: a combined neurophysiological and neuroimaging approach (R1)	can't access
M. Stangel; I. K. Penner; B. A. Kallmann; C. Lukas; B. C. Kieseier	2015	Towards the implementation of ‘no evidence of disease activity' in multiple sclerosis treatment: The multiple sclerosis decision model	no D/F
M. Summers; J. Swanton; K. Fernando; C. Dalton; D. H. Miller; L. Cipolotti; M. A. Ron	2008	Cognitive impairment in multiple sclerosis can be predicted by imaging early in the disease	do not correlate MRI to D/F
M. Yildiz; F. Brugger; N. Kästle; B. Tettenborn	2016	Neurocognitive impairment is associated with corpus callosum atrophy in multiple sclerosis	Mixed MS: Includes other MS types in the MS group
M. Zorzon; R. Zivadinov; L. Locatelli; B. Stival; D. Nasuelli; A. Bratina; A. Bosco; M. A. Tommasi; R. S. Pozzi Mucelli; M. Ukmar; G. Cazzato	2003	Correlation of sexual dysfunction and brain magnetic resonance imaging in multiple sclerosis	do not correlate MRI to D/F
Manca, R., Mitolo, M., Wilkinson, I., Paling, D., Sharrack, B., Venneri, A.	2021	A network-based cognitive training induces cognitive improvements and neuroplastic changes in patients with relapsing-remitting multiple sclerosis: An exploratory case-control study	Treatmen: non-drug intervention study
N. Bergsland; R. Zivadinov; M. G. Dwyer; B. Weinstock-Guttman; R. H. B. Benedict	2016	Localized atrophy of the thalamus and slowed cognitive processing speed in MS patients	do not correlate MRI to D/F
N. Derache; B. Grassiot; F. Mézenge; A. Emmanuelle Dugué; B. Desgranges; J. M. Constans; G. L. Defer	2013	Fatigue is associated with metabolic and density alterations of cortical and deep gray matter in Relapsing-Remitting-Multiple Sclerosis patients at the earlier stage of the disease: A PET/MR study	wrong D/F test
N. Téllez; J. Alonso; J. Río; M. Tintoré; C. Nos; X. Montalban; A. Rovira	2008	The basal ganglia: a substrate for fatigue in multiple sclerosis	Final Inclusion
Nabizadeh, F., Balabandian, M., Rostami, M.R., Owji, M., Sahraian, M.A., Bidadian, M., Ghadiri, F., Rezaeimanesh, N., Moghadasi, A.N.	2022	Association of cognitive impairment and quality of life in patients with multiple sclerosis: A cross-sectional study	do not correlate MRI to D/F

Nath, S.R., Grewal, P., Cho, T., Mao-Draayer, Y.	2022	Familial multiple sclerosis in patients with Von Hippel-Lindau disease	do not correlate MRI to D/F
Newland, P., Chen, L., Sun, P., Zempel, J.	2021	Neurophysiological Correlates of Fatigue in Multiple Sclerosis	Sample size <20
O. O. Kopchak; T. A. Odintsova	2021	Cognitive impairment and depression in patients with relapsing-remitting multiple sclerosis depending on age and neuroimaging findings	Final Inclusion
O. Svolgaard; K. W. Andersen; C. Bauer; K. H. Madsen; M. Blinkenberg; F. Selleberg; H. R. Siebner	2018	Cerebellar and premotor activity during a non-fatiguing grip task reflects motor fatigue in relapsing-remitting multiple sclerosis	Final Inclusion
O. Svolgaard; K. W. Andersen; C. Bauer; K. H. Madsen; M. Blinkenberg; F. Sellebjerg; H. R. Siebner	2022	Mapping grip-force related brain activity after a fatiguing motor task in multiple sclerosis	Final Inclusion
Ö. Yaldizli; I. K. Penner; T. Yonekawa; Y. Naegelin; J. Kuhle; M. Pardini; D. T. Chard; C. Stippich; J. I. Kira; K. Bendfeldt; M. Amann; E. W. Radue; L. Kappos; T. Sprenger	2016	The association between olfactory bulb volume, cognitive dysfunction, physical disability and depression in multiple sclerosis	Final Inclusion
Ö. Yaldizli; S. Glassl; D. Sturm; A. Papadopoulou; A. Gass; B. Tettenborn; N. Putzki	2011	Fatigue and progression of corpus callosum atrophy in multiple sclerosis	Final Inclusion
Ogisu, K., Niino, M., Miyazaki, Y., Kikuchi, S.	2021	Optimal indicator for histogram analysis of fractional anisotropy for normal-appearing white matter in multiple sclerosis	Sample size <20
Ooi, S., Kalincik, T., Perucca, P., Monif, M.	2021	The prevalence of epileptic seizures in multiple sclerosis in a large tertiary hospital in Australia	do not correlate MRI to D/F
P. Puz; A. Lasek-Bal; A. Steposz; K. Bartoszek	2018	Effect of comorbidities on the course of multiple sclerosis	do not correlate MRI to D/F
Palotai, M., Wallack, M., Kujbus, G., Dalnoki, A., Guttmann, C.	2021	Usability of a mobile app for real-time assessment of fatigue and related symptoms in patients with multiple sclerosis: Observational study	no MRI
Parray, Z., Zargar, M.H., Asimi, R., Dar, W.R., Yaqoob, A., Raina, A., Ganie, H., Wani, M., Shah, Z.A.	2022	Interleukin 32 gene promoter polymorphism: A genetic risk factor for multiple sclerosis in Kashmiri population	no MRI
R. Riccelli; L. Passamonti; A. Cerasa; S. Nigro; S. M. Cavalli; C. Chiriaco; P. Valentino; R. Nisticò; A. Quattrone	2016	Individual differences in depression are associated with abnormal function of the limbic system in multiple sclerosis patients	Final Inclusion
R. Righart; V. Biberacher; L. E. Jonkman; R. Klaver; P. Schmidt; D. Buck; A. Berthele; J. S. Kirschke; C. Zimmer; B. Hemmer; J. J. G. Geurts; M. Mühlau	2017	Cortical pathology in multiple sclerosis detected by the T1/T2-weighted ratio from routine magnetic resonance imaging	do not correlate MRI to D/F
R. Zivadinov; J. Sepcic; D. Nasuelli; R. De Masi; L. M. Bragadin; M. A. Tommasi; S. Zambito-Marsala; R. Moretti; A. Bratina; M. Ukmar; R. S. Pozzi-Mucelli; A. Grop; G. Cazzato; M. Zorzon	2001	A longitudinal study of brain atrophy and cognitive disturbances in the early phase of relapsing-remitting multiple sclerosis	do not correlate MRI to D/F
R. Zivadinov; M. Zorzon; L. Locatelli; B. Stival; F. Monti; D. Nasuelli; M. A. Tommasi; A. Bratina; G. Cazzato	2003	Sexual dysfunction in multiple sclerosis: A MRI, neurophysiological and urodynamic study	no D/F

Rocca, M.A., Valsasina, P., Colombo, B., Martinelli, V., Filippi, M.	2021	Cortico-subcortical functional connectivity modifications in fatigued multiple sclerosis patients treated with fampridine and amantadine	Treatment: DMT
Rojas, J.I., Patrucco, L., Alonso, R., Garcea, O., Deri, N., Carnero Contentti, E., Lopez, P.A., Pettinicchi, J.P., Caride, A., Cristiano, E.	2021	Diagnostic uncertainty during the transition to secondary progressive multiple sclerosis: Multicenter study in Argentina	do not correlate MRI to D/F
S. A. Mohamed; O. El-Deib	2014	Depressive symptoms as a predictor of outcome in patients with multiple sclerosis	no MRI
S. Barone; M. E. Caligiuri; P. Valentino; A. Cherubini; C. Chiriaco; A. Granata; E. Filippelli; T. Tallarico; R. Nistico; A. Quattrone	2018	Multimodal assessment of normal-appearing corpus callosum is a useful marker of disability in relapsing-remitting multiple sclerosis: an MRI cluster analysis study	do not correlate MRI to D/F
S. Cader; A. Cifelli; Y. Abu-Omar; J. Palace; P. M. Matthews	2006	Reduced brain functional reserve and altered functional connectivity in patients with multiple sclerosis	no D/F
S. Collorone; N. Cawley; F. Grussu; F. Prados; F. Tona; A. Calvi; B. Kanber; T. Schneider; L. Kipp; H. Zhang; D. C. Alexander; A. J. Thompson; A. Toosy; C. A. M. G. Wheeler-Kingshott; O. Ciccarelli	2020	Reduced neurite density in the brain and cervical spinal cord in relapsing–remitting multiple sclerosis: A NODDI study	no D/F
S. Golde; J. Heine; J. Pöttgen; M. Mantwill; S. Lau; K. Wingenfeld; C. Otte; I. K. Penner; A. K. Engel; C. Heesen; J. P. Stellmann; I. Dziobek; C. Finke; S. M. Gold	2020	Distinct Functional Connectivity Signatures of Impaired Social Cognition in Multiple Sclerosis	Final Inclusion
S. Jaeger; F. Paul; M. Scheel; A. Brandt; J. Heine; D. Pach; C. M. Witt; J. Bellmann-Strobl; C. Finke	2019	Multiple sclerosis-related fatigue: Altered resting-state functional connectivity of the ventral striatum and dorsolateral prefrontal cortex	Final Inclusion
S. M. Gold; K. C. Kern; M. F. O'Connor; M. J. Montag; A. Kim; Y. S. Yoo; B. S. Giesser; N. L. Sicotte	2010	Smaller cornu ammonis 2-3/dentate gyrus volumes and elevated cortisol in multiple sclerosis patients with depressive symptoms	Final Inclusion
S. Nigro; L. Passamonti; R. Riccelli; N. Toschi; F. Rocca; P. Valentino; R. Nisticò; F. Fera; A. Quattrone	2015	Structural 'connectomic' alterations in the limbic system of multiple sclerosis patients with major depression	Final Inclusion
S. P. Hojjat; C. G. Cantrell; T. J. Carroll; R. Vitorino; A. Feinstein; L. Zhang; S. P. Symons; S. A. Morrow; L. Lee; P. O'Connor; R. I. Aviv	2016	Perfusion reduction in the absence of structural differences in cognitively impaired versus unimpaired RRMS patients	do not correlate MRI to D/F
S. Rossi; V. Studer; C. Motta; S. Polidoro; J. Perugini; G. Macchiarulo; A. M. Giovannetti; L. Pareja-Gutierrez; A. Calò; I. Colonna; R. Furlan; G. Martino; D. Centonze	2017	Neuroinflammation drives anxiety and depression in relapsing-remitting multiple sclerosis	do not correlate MRI to D/F
S. Sevim	2016	Relapses in multiple sclerosis: Definition, pathophysiology, features, imitators, and treatment	no D/F
Saruhan, E., Korkmaz, M., Altiparmak, B., Tosun, K., Kutlu, G.	2022	COMPARISON OF OREXIN-A AND NEUROFILAMENT LIGHT CHAIN LEVELS IN PATIENTS WITH RELAPSING-REMITTING MULTIPLE SCLEROSIS: A PILOT STUDY [OREXIN-A- ÉS NEUROFILAMENTUM-	no MRI

		KÖNNYÛLÁNC FEHÉRJESZINTEK RELAPSZÁLÓ-REMITTÁLÓ SCLEROSIS MULTIPLEXBEN SZENVEDÔKNÉL: PILOT VIZSGÁLAT]	
Soares, J.M., Conde, R., Magalhães, R., Marques, P., Magalhães, R., Gomes, L., Gonçalves, Ó.F., Arantes, M., Sampaio, A.	2021	Alterations in functional connectivity are associated with white matter lesions and information processing efficiency in multiple sclerosis	do not correlate MRI to D/F
Stascheit, F., Li, L., Mai, K., Baum, K., Siebert, E., Ruprecht, K.	2021	Delayed onset hypophysitis after therapy with daclizumab for multiple sclerosis – A report of two cases	Treatment: DMT
T. A. Hassan; S. F. Elkholy; B. E. Mahmoud; M. ElSherbiny	2019	Multiple sclerosis and depressive manifestations: can diffusion tensor MR imaging help in the detection of microstructural white matter changes?	Final Inclusion
T. K. Yoldas; H. D. Keklikoglu; O. Zengin; E. B. Solak; S. Keskin	2010	Relation of Serum Uric Acid Level with Cognitive Functions and Number of Plaques in Patients with Relapsing-Remitting Multiple Sclerosis	no D/F
T. Štecková; P. Hluštík; V. Sládková; F. Odstrčil; J. Mareš; P. Kaňovský	2014	Thalamic atrophy and cognitive impairment in clinically isolated syndrome and multiple sclerosis	Final Inclusion
Trufanov, A., Bisaga, G., Skulyabin, D., Temniy, A., Poplyak, M., Chakchir, O., Efimtsev, A., Dmitriy, T., Odinak, M., Litvinenko, I.	2021	Thalamic nuclei degeneration in multiple sclerosis	do not correlate MRI to D/F
V. Biberacher; C. C. Boucard; P. Schmidt; C. Engl; D. Buck; A. Berthele; M. M. Hoshi; C. Zimmer; B. Hemmer; M. Muhlau	2015	Atrophy and structural variability of the upper cervical cord in early multiple sclerosis	Spinal cord
V. M. Leavitt; E. De Meo; G. Riccitelli; M. A. Rocca; G. Comi; M. Filippi; J. F. Sumowski	2015	Elevated body temperature is linked to fatigue in an Italian sample of relapsing-remitting multiple sclerosis patients	do not correlate MRI to D/F
V. Martinovic; I. Nikolic; S. Mesaros; J. Drulovic	2020	Bilateral horizontal gaze palsy in benign multiple sclerosis	no D/F
Waliszewska-Prosół, M., Nowakowska-Kotas, M., Misiak, B., Chojdak-Łukasiewicz, J., Budrewicz, S., Pokryszko-Dragan, A.	2022	Allostatic load index in patients with multiple sclerosis: A case-control study	no MRI
Y. Benesova; I. Niedermayerova; M. Mechl; P. Havlikova	2003	The relation between brain MRI lesions and depressive symptoms in multiple sclerosis	Final Inclusion
Y. Shen; L. Bai; Y. Gao; F. Cui; Z. Tan; Y. Tao; C. Sun; L. Zhou	2014	Depressive symptoms in multiple sclerosis from an in vivo study with TBSS	Sample size <20
Yalachkov, Y., Anschuetz, V., Jakob, J., Schaller-Paule, M.A., Schaefer, J.H., Reilaender, A., Friedauer, L., Behrens, M., Foerch, C.	2021	C-Reactive Protein Levels and Gadolinium-Enhancing Lesions Are Associated With the Degree of Depressive Symptoms in Newly Diagnosed Multiple Sclerosis	Mixed MS: Did not separate between MS subgroups in analysis
Zanghì, A., D'Amico, E., Lo Fermo, S., Patti, F.	2021	Exploring polypharmacy phenomenon in newly diagnosed relapsing–remitting multiple sclerosis: a cohort ambispective single-centre study	Treatment: DMT
