# Supplementary material for: Brain connectivity changes underlying depression and fatigue in relapsing-remitting multiple sclerosis: A systematic review
Source: PLoS One. 2024 Mar 29;19(3):e0299634. doi: 10.1371/journal.pone.0299634 (PMC10980255; doi:10.1371/journal.pone.0299634)
Supplement: S2 Table — (PDF) [file pone.0299634.s005.pdf]

S2 Table. Data extraction table.

DOI	Paper	Author	Type of study - Longitudinal or cross sectional	Part of brain studied	MRI measures	MRI full report	Machine + Field strength	Participants - N for each group	Participants age range; Median/mean age	EDSS score median/mean (or range if median not given)	Fatigue - What assessment method was used and if cut off values were used	Depression - What assessment method was used: if cut off values were used	Major findings - Differences (or no differences) between groups and in what regions (p-values, CI, correlation coefficient, etc.)	Comments
