# Supplementary material for: Brain connectivity changes underlying depression and fatigue in relapsing-remitting multiple sclerosis: A systematic review
Source: PLoS One. 2024 Mar 29;19(3):e0299634. doi: 10.1371/journal.pone.0299634 (PMC10980255; doi:10.1371/journal.pone.0299634)
Supplement: S5 Table — (PDF) [file pone.0299634.s008.pdf]

S5 Table. Full breakdown of AXIS scores [1]. ns = not stated, considered as a negative score.

Study	Study design				Selection bias				Comparability/Method					Outcomes				Total	
	Were the aims/objectives of the study clear?	Was the study design appropriate for the stated aim(s)?	Was the sample size justified (based on pre-study considerations of statistical power)?	Was the target/reference population clearly defined? (Is it clear who the research was about?)	Was the sample frame taken from an appropriate population base so that it closely represented the target/reference population under investigation?	Was the selection process likely to select subjects/participants that were representative of the target/reference population under investigation?	Was ethical aspect approval or consent of participants attained?	Were the risk factor and outcome variable measured appropriate to the aims of the study?	Were the risk factor and outcome variables measured correctly using instruments/ measurements been trialled, piloted or published previously?	Is it clear what was used to determined statistical significance and/or precisions estimates? (e.g. p-values, confidence intervals)	Were the methods (including statistical methods) sufficiently described to enable them to be repeated?	Are the basic data adequately described?	Were the results internally consistent?	Were the results presented for all the analyses described in the method?	Were the author's discussions and conclusions justified by the results?	Were the limitations of the study discussed?	Were there any funding sources or conflicts of interest that may affect the authors' interpretation of the results?	Score	Percentage
Alshehri et al. [2]	Y	Y	N	Y	Y	Y	Y	Y	Y	Y	Y	Y	Y	Y	Y	Y	N	16	94
Altermatt et al. [3]	Y	Y	N	Y	Y	Y	Y	Y	Y	Y	Y	Y	Y	Y	Y	Y	N	16	94
Andreasen et al. [4]	Y	Y	N	Y	Y	Y	Y	Y	Y	Y	Y	Y	Y	Y	Y	Y	N	16	94
Bauer et al. [5]	Y	Y	N	Y	N	Y	Y	Y	Y	Y	Y	Y	Y	Y	Y	Y	N	15	88
Beaudoin et al. [6]	Y	Y	N	Y	Y	Y	Y	Y	Y	Y	Y	Y	Y	Y	Y	Y	N	16	94
Benesova et al. [7]	Y	Y	N	Y	Y	Y	N	Y	Y	Y	Y	N	Y	Y	Y	N	ns	12	71
Bisecco et al. [8]	Y	Y	N	Y	Y	Y	Y	Y	Y	Y	Y	Y	Y	Y	Y	Y	N	16	94
Carotenuto et al. [9]	Y	Y	N	Y	Y	Y	Y	Y	Y	Y	Y	Y	Y	Y	Y	Y	N	16	94
Codella et al. [10]	Y	Y	N	Y	Y	Y	N	Y	Y	Y	Y	Y	Y	Y	Y	N	ns	13	76
Damasceno et al. [11]	Y	Y	N	Y	Y	Y	Y	Y	Y	Y	Y	Y	Y	Y	N	Y	N	15	88
Filippi et al. [12]	Y	Y	N	Y	Y	Y	Y	Y	Y	Y	Y	Y	Y	Y	Y	N	N	15	88
Finke et al. [13]	Y	Y	N	Y	Y	Y	Y	Y	Y	Y	Y	Y	Y	Y	Y	N	N	15	88
Gold et al. [14]	Y	y	N	Y	Y	Y	Y	Y	Y	Y	Y	Y	Y	Y	Y	Y	N	16	94
Golde et al. [15]	Y	Y	Y	Y	N	Y	Y	Y	Y	Y	Y	Y	Y	Y	Y	Y	N	16	94
Cruz Gomez et al. [16]	Y	Y	Y	Y	N	Y	Y	Y	Y	N	Y	Y	Y	Y	Y	Y	N	15	88

Hassan et al. [17]	Y	Y	Y	Y	N	Y	Y	Y	Y	N	Y	N	N	Y	Y	Y	N	13	76
Hildebrandt et al. [18]	Y	Y	N	Y	Y	Y	Y	Y	Y	Y	Y	Y	Y	Y	Y	Y	ns	15	88
Huang et al. [19]	Y	Y	N	Y	Y	Y	Y	Y	Y	Y	Y	Y	Y	Y	Y	Y	N	16	94
Iancheva et al. [20]	Y	Y	N	Y	Y	Y	Y	Y	Y	Y	Y	N	Y	N	Y	Y	N	14	82
Jaeger et al. [21]	Y	Y	N	Y	Y	Y	Y	Y	Y	Y	Y	Y	Y	Y	Y	Y	N	16	94
Kever et al. [22]	Y	Y	N	Y	Y	Y	Y	Y	Y	Y	Y	N	Y	Y	Y	Y	N	15	88
Khedr et al. [23]	Y	Y	N	Y	Y	Y	Y	Y	Y	Y	Y	Y	Y	Y	Y	Y	N	16	94
Kopchak and Odintsova [22]	Y	Y	N	Y	Y	Y	Y	N	N	Y	N	N	Y	Y	N	N	N	10	59
Lazzarotto et al. [24]	Y	Y	N	Y	Y	Y	Y	Y	Y	Y	Y	Y	N	Y	Y	Y	N	15	88
Morgante et al. [25]	Y	Y	N	Y	Y	Y	Y	Y	Y	Y	Y	Y	Y	Y	Y	Y	N	16	94
Niepel et al. [26]	Y	Y	N	Y	Y	Y	Y	Y	Y	Y	Y	Y	Y	Y	Y	N	ns	14	82
Nigro et al. [27]	Y	Y	N	Y	Y	Y	Y	Y	Y	Y	Y	Y	Y	Y	Y	Y	N	16	94
Nygaard et al. [28]	Y	Y	N	Y	Y	Y	Y	Y	Y	Y	Y	Y	Y	Y	Y	Y	N	16	94
Pardini et al. [29]	Y	Y	N	Y	Y	Y	Y	Y	Y	Y	Y	Y	Y	Y	Y	N	ns	14	82
Pardini et al. [30]	Y	Y	N	Y	Y	Y	ns	Y	Y	N	Y	N	Y	Y	Y	Y	N	13	76
Pokryszko-Dragan et al. [31]	Y	Y	N	Y	Y	Y	Y	Y	Y	Y	Y	Y	Y	Y	Y	Y	N	16	94
Pravatà et al. [32]	Y	Y	N	Y	Y	Y	Y	Y	Y	Y	Y	Y	Y	Y	Y	Y	N	16	94
Riccelli et al. [33]	Y	Y	N	Y	Y	Y	Y	Y	Y	Y	Y	Y	Y	Y	Y	Y	N	16	94
Rocca et al. [34]	Y	Y	N	Y	Y	Y	Y	Y	Y	Y	Y	Y	Y	Y	Y	Y	N	16	94
Rocca et al. [35]	Y	Y	N	Y	Y	Y	Y	Y	Y	Y	Y	Y	Y	Y	Y	Y	N	16	94
Rojas et al. [36]	Y	Y	N	Y	Y	Y	Y	Y	Y	Y	Y	Y	Y	Y	Y	N	N	15	88
Romanello et al. [37]	Y	Y	N	Y	Y	Y	Y	Y	Y	Y	Y	Y	Y	Y	Y	Y	N	16	94
Ruiz-Rizzo et al. [38]	Y	Y	N	Y	Y	Y	Y	Y	Y	Y	Y	Y	Y	Y	Y	Y	N	16	94
Saberi et al. [39]	Y	Y	N	Y	Y	Y	Y	Y	Y	Y	Y	N	Y	Y	Y	Y	N	15	88
Specogna et al. [40]	Y	Y	N	Y	Y	Y	Y	Y	Y	Y	Y	Y	Y	Y	Y	N	N	15	88
Štecková et al. [41]	Y	Y	N	Y	Y	Y	Y	Y	Y	Y	Y	Y	Y	Y	Y	Y	N	16	94
Svolgaard et al. [42]	Y	Y	N	Y	N	Y	Y	Y	Y	Y	Y	Y	Y	Y	Y	Y	ns	14	82
Svolgaard et al. [43]	N	Y	N	Y	Y	Y	Y	Y	Y	Y	Y	Y	Y	Y	Y	Y	N	16	94
Téllez et al. [44]	Y	Y	N	Y	Y	Y	Y	Y	Y	Y	Y	Y	Y	Y	Y	N	N	15	88
Tomasevic et al. [45]	Y	Y	N	Y	Y	Y	Y	Y	Y	Y	Y	Y	Y	Y	Y	N	N	15	88
Wilting et al. [46]	Y	Y	N	Y	Y	Y	Y	Y	Y	Y	Y	Y	Y	Y	Y	N	N	15	88
Wu et al. [47]	Y	Y	N	Y	Y	Y	Y	Y	Y	Y	Y	Y	Y	Y	Y	Y	N	16	94
Wu et al. [48]	Y	Y	Y	Y	N	Y	Y	Y	Y	Y	Y	Y	Y	Y	Y	Y	N	16	94
Yaldizli et al. [49]	Y	Y	N	y	Y	Y	Y	Y	Y	Y	Y	Y	Y	Y	Y	Y	N	16	94
Zellini et al. [50]	Y	Y	N	N	Y	N	Y	N	Y	Y	Y	Y	Y	Y	Y	N	N	12	71

Zhou et al. [51]	Y	Y	Y	Y	N	Y	Y	Y	Y	Y	Y	Y	Y	Y	Y	Y	N	16	94
Zhou et al. [52]	Y	Y	N	Y	Y	Y	Y	Y	Y	Y	Y	Y	Y	Y	Y	Y	N	16	94

References:

1. Downes MJ, Brennan ML, Williams HC, Dean RS. Development of a critical appraisal tool to assess the quality of cross-sectional studies (AXIS). BMJ open. 2016;6(12):e011458.
2. Alshehri A, Al-iedani O, Arm J, Gholizadeh N, Billiet T, Lea R, et al. Neural diffusion tensor imaging metrics correlate with clinical measures in people with relapsing-remitting MS. Neuroradiology Journal. 2022;35(5):592-9. doi: 10.1177/19714009211067400.
3. Altermatt A, Gaetano L, Magon S, Häring DA, Tomic D, Wuerfel J, et al. Clinical Correlations of Brain Lesion Location in Multiple Sclerosis: Voxel-Based Analysis of a Large Clinical Trial Dataset. Brain Topogr. 2018;31(5):886-94. Epub 20180529. doi: 10.1007/s10548-018-0652-9. PubMed PMID: 29845492.
4. Andreasen AK, Jakobsen J, Soerensen L, Andersen H, Petersen T, Bjarkam CR, et al. Regional brain atrophy in primary fatigued patients with multiple sclerosis. Neuroimage. 2010;50(2):608-15. Epub 20100106. doi: 10.1016/j.neuroimage.2009.12.118. PubMed PMID: 20060048.
5. Bauer C, Dyrby TB, Sellebjerg F, Madsen KS, Svolgaard O, Blinkenberg M, et al. Motor fatigue is associated with asymmetric connectivity properties of the corticospinal tract in multiple sclerosis. Neuroimage-Clinical. 2020;28. doi: 10.1016/j.nicl.2020.102393. PubMed PMID: WOS:000600619100036.
6. Beaudoin AM, Rheault F, Theaud G, Laberge F, Whittingstall K, Lamontagne A, et al. Modern Technology in Multi-Shell Diffusion MRI Reveals Diffuse White Matter Changes in Young Adults With Relapsing-Remitting Multiple Sclerosis. Frontiers in Neuroscience. 2021;15:13. doi: 10.3389/fnins.2021.665017. PubMed PMID: WOS:000687832800001.
7. Benesova Y, Niedermayerova I, Mechl M, Havlikova P. The relation between brain MRI lesions and depressive symptoms in multiple sclerosis. Bratisl Lek Listy. 2003;104(4-5):174-6. PubMed PMID: 14604264.
8. Bisecco A, Caiazzo G, d'Ambrosio A, Sacco R, Bonavita S, Docimo R, et al. Fatigue in multiple sclerosis: The contribution of occult white matter damage. Mult Scler. 2016;22(13):1676-84. Epub 20160204. doi: 10.1177/1352458516628331. PubMed PMID: 26846989.
9. Carotenuto A, Wilson H, Giordano B, Caminiti SP, Chappell Z, Williams SCR, et al. Impaired connectivity within neuromodulatory networks in multiple sclerosis and clinical implications. J Neurol. 2020;267(7):2042-53. Epub 20200326. doi: 10.1007/s00415-020-09806-3. PubMed PMID: 32219555; PubMed Central PMCID: PMCPMC7320961.
10. Codella M, Rocca MA, Colombo B, Martinelli-Boneschi F, Comi G, Filippi M. Cerebral grey matter pathology and fatigue in patients with multiple sclerosis: a preliminary study. J Neurol Sci. 2002;194(1):71-4. doi: 10.1016/s0022-510x(01)00682-7. PubMed PMID: 11809169.
11. Damasceno A, Damasceno BP, Cendes F. Atrophy of reward-related striatal structures in fatigued MS patients is independent of physical disability. Mult Scler. 2016;22(6):822-9. Epub 20150803. doi: 10.1177/1352458515599451. PubMed PMID: 26238465.
12. Filippi M, Rocca MA, Colombo B, Falini A, Codella M, Scotti G, et al. Functional magnetic resonance imaging correlates of fatigue in multiple sclerosis. Neuroimage. 2002;15(3):559-67. doi: 10.1006/nimg.2001.1011. PubMed PMID: 11848698.

13. Finke C, Schlichting J, Papazoglou S, Scheel M, Freing A, Soemmer C, et al. Altered basal ganglia functional connectivity in multiple sclerosis patients with fatigue. Mult Scler. 2015;21(7):925-34. Epub 20141112. doi: 10.1177/1352458514555784. PubMed PMID: 25392321.
14. Gold SM, Kern KC, O'Connor MF, Montag MJ, Kim A, Yoo YS, et al. Smaller cornu ammonis 2-3/dentate gyrus volumes and elevated cortisol in multiple sclerosis patients with depressive symptoms. Biol Psychiatry. 2010;68(6):553-9. Epub 20100619. doi: 10.1016/j.biopsych.2010.04.025. PubMed PMID: 20646680; PubMed Central PMCID: PMCPMC3122328.
15. Golde S, Heine J, Pöttgen J, Mantwill M, Lau S, Wingenfeld K, et al. Distinct Functional Connectivity Signatures of Impaired Social Cognition in Multiple Sclerosis. Frontiers in Neurology. 2020;11. doi: 10.3389/fneur.2020.00507.
16. Cruz Gomez AJ, Campos NV, Belenguer A, Avila C, Forn C. Regional Brain Atrophy and Functional Connectivity Changes Related to Fatigue in Multiple Sclerosis. Plos One. 2013;8(10). doi: 10.1371/journal.pone.0077914. PubMed PMID: WOS:000326034500049.
17. Hassan TA, Elkholy SF, Mahmoud BE, ElSherbiny M. Multiple sclerosis and depressive manifestations: can diffusion tensor MR imaging help in the detection of microstructural white matter changes? Egyptian Journal of Radiology and Nuclear Medicine. 2019;50(1). doi: 10.1186/s43055-019-0033-8. PubMed PMID: WOS:000486165900001.
18. Hildebrandt H, Hahn HK, Kraus JA, Schulte-Herbrüggen A, Schwarze B, Schwendemann G. Memory performance in multiple sclerosis patients correlates with central brain atrophy. Mult Scler. 2006;12(4):428-36. doi: 10.1191/1352458506ms1286oa. PubMed PMID: 16900756.
19. Huang M, Zhou F, Wu L, Wang B, Wan H, Li F, et al. Synchronization within, and interactions between, the default mode and dorsal attention networks in relapsing-remitting multiple sclerosis. Neuropsychiatr Dis Treat. 2018;14:1241-52. Epub 20180514. doi: 10.2147/ndt.S155478. PubMed PMID: 29795982; PubMed Central PMCID: PMCPMC5957478.
20. Iancheva D, Trenova A, Mantarovau S, Terziyski K. Functional Magnetic Resonance Imaging Correlations Between Fatigue and Cognitive Performance in Patients With Relapsing Remitting Multiple Sclerosis. Frontiers in Psychiatry. 2019;10. doi: 10.3389/fpsyt.2019.00754. PubMed PMID: WOS:000496145700001.
21. Jaeger S, Paul F, Scheel M, Brandt A, Heine J, Pach D, et al. Multiple sclerosis-related fatigue: Altered resting-state functional connectivity of the ventral striatum and dorsolateral prefrontal cortex. Mult Scler. 2019;25(4):554-64. Epub 20180221. doi: 10.1177/1352458518758911. PubMed PMID: 29464981.
22. Kopchak OO, Odintsova TA. Cognitive impairment and depression in patients with relapsing-remitting multiple sclerosis depending on age and neuroimaging findings. Egypt J Neurol Psychiatr Neurosurg. 2021;57(1):119. Epub 20210908. doi: 10.1186/s41983-021-00376-3. PubMed PMID: 34511865; PubMed Central PMCID: PMCPMC8424158.
23. Khedr EM, Desoky T, Gamea A, Ezzeldin MY, Zaki AF. Fatigue and brain atrophy in Egyptian patients with relapsing remitting multiple sclerosis. Multiple Sclerosis and Related Disorders. 2022;63:6. doi: 10.1016/j.msard.2022.103841. PubMed PMID: WOS:000832865100007.
24. Lazzarotto A, Margoni M, Franciotta S, Zywicki S, Riccardi A, Poggiali D, et al. Selective Cerebellar Atrophy Associates with Depression and Fatigue in the Early Phases of Relapse-Onset Multiple Sclerosis. Cerebellum. 2020;19(2):192-200. doi: 10.1007/s12311-019-01096-4. PubMed PMID: 31898280.
25. Morgante F, Dattola V, Crupi D, Russo M, Rizzo V, Ghilardi MF, et al. Is central fatigue in multiple sclerosis a disorder of movement preparation? J Neurol. 2011;258(2):263-72. Epub 20100922. doi: 10.1007/s00415-010-5742-x. PubMed PMID: 20859746.

26. Niepel G, Tench Ch R, Morgan PS, Evangelou N, Auer DP, Constantinescu CS. Deep gray matter and fatigue in MS: a T1 relaxation time study. J Neurol. 2006;253(7):896-902. Epub 20060313. doi: 10.1007/s00415-006-0128-9. PubMed PMID: 16525881.
27. Nigro S, Passamonti L, Riccelli R, Toschi N, Rocca F, Valentino P, et al. Structural 'connectomic' alterations in the limbic system of multiple sclerosis patients with major depression. Mult Scler. 2015;21(8):1003-12. Epub 20141222. doi: 10.1177/1352458514558474. PubMed PMID: 25533294.
28. Nygaard GO, Walhovd KB, Sowa P, Chepkoech JL, Bjørnerud A, Due-Tønnessen P, et al. Cortical thickness and surface area relate to specific symptoms in early relapsing-remitting multiple sclerosis. Mult Scler. 2015;21(4):402-14. Epub 20140819. doi: 10.1177/1352458514543811. PubMed PMID: 25139946.
29. Pardini M, Bonzano L, Mancardi GL, Roccatagliata L. Frontal networks play a role in fatigue perception in multiple sclerosis. Behav Neurosci. 2010;124(3):329-36. doi: 10.1037/a0019585. PubMed PMID: 20528076.
30. Pardini M, Bonzano L, Bergamino M, Bommarito G, Feraco P, Murugavel A, et al. Cingulum bundle alterations underlie subjective fatigue in multiple sclerosis. Mult Scler. 2015;21(4):442-7. Epub 20140821. doi: 10.1177/1352458514546791. PubMed PMID: 25145692.
31. Pokryszko-Dragan A, Banaszek A, Nowakowska-Kotas M, Jeżowska-Jurczyk K, Dziadkowiak E, Gruszka E, et al. Diffusion tensor imaging findings in the multiple sclerosis patients and their relationships to various aspects of disability. J Neurol Sci. 2018;391:127-33. Epub 20180613. doi: 10.1016/j.jns.2018.06.007. PubMed PMID: 30103962.
32. Pravatà E, Zecca C, Sestieri C, Caulo M, Riccitelli GC, Rocca MA, et al. Hyperconnectivity of the dorsolateral prefrontal cortex following mental effort in multiple sclerosis patients with cognitive fatigue. Mult Scler. 2016;22(13):1665-75. Epub 20160204. doi: 10.1177/1352458515625806. PubMed PMID: 26846988.
33. Riccelli R, Passamonti L, Cerasa A, Nigro S, Cavalli SM, Chiriaco C, et al. Individual differences in depression are associated with abnormal function of the limbic system in multiple sclerosis patients. Mult Scler. 2016;22(8):1094-105. Epub 20151009. doi: 10.1177/1352458515606987. PubMed PMID: 26453680.
34. Rocca MA, Gatti R, Agosta F, Broglia P, Rossi P, Riboldi E, et al. Influence of task complexity during coordinated hand and foot movements in MS patients with and without fatigue. A kinematic and functional MRI study. J Neurol. 2009;256(3):470-82. Epub 20090306. doi: 10.1007/s00415-009-0116-y. PubMed PMID: 19271107.
35. Rocca MA, Meani A, Riccitelli GC, Colombo B, Rodegher M, Falini A, et al. Abnormal adaptation over time of motor network recruitment in multiple sclerosis patients with fatigue. Mult Scler. 2016;22(9):1144-53. Epub 20151022. doi: 10.1177/1352458515614407. PubMed PMID: 26493126.
36. Rojas JI, Sanchez F, Patrucco L, Miguez J, Besada C, Cristiano E. Brain structural changes in patients in the early stages of multiple sclerosis with depression. Neurol Res. 2017;39(7):596-600. Epub 20170301. doi: 10.1080/01616412.2017.1298279. PubMed PMID: 28245725.
37. Romanello A, Krohn S, von Schwanenflug N, Chien C, Bellmann-Strobl J, Ruprecht K, et al. Functional connectivity dynamics reflect disability and multi-domain clinical impairment in patients with relapsing-remitting multiple sclerosis. Neuroimage Clin. 2022;36:103203. Epub 20220916. doi: 10.1016/j.nicl.2022.103203. PubMed PMID: 36179389; PubMed Central PMCID: PMCPMC9668632.
38. Ruiz-Rizzo AL, Bublak P, Kluckow S, Finke K, Gaser C, Schwab M, et al. Neural distinctiveness of fatigue and low sleep quality in multiple sclerosis. European Journal of Neurology. 2022;29(10):3017-27. doi: 10.1111/ene.15445.

39. Saberi A, Abdolalizadeh A, Mohammadi E, Nahayati MA, Bagheri H, Shekarchi B, et al. Thalamic shape abnormalities in patients with multiple sclerosis-related fatigue. Neuroreport. 2021;32(6):438-42. doi: 10.1097/wnr.0000000000001616. PubMed PMID: 33788816.
40. Specogna I, Casagrande F, Lorusso A, Catalan M, Gorian A, Zugna L, et al. Functional MRI during the execution of a motor task in patients with multiple sclerosis and fatigue. Radiol Med. 2012;117(8):1398-407. Epub 20120622. doi: 10.1007/s11547-012-0845-3. PubMed PMID: 22729506.
41. Štecková T, Hluštík P, Sládková V, Odstrčil F, Mareš J, Kaňovský P. Thalamic atrophy and cognitive impairment in clinically isolated syndrome and multiple sclerosis. J Neurol Sci. 2014;342(1-2):62-8. Epub 20140430. doi: 10.1016/j.jns.2014.04.026. PubMed PMID: 24819917.
42. Svolgaard O, Andersen KW, Bauer C, Madsen KH, Blinkenberg M, Selleberg F, et al. Cerebellar and premotor activity during a non-fatiguing grip task reflects motor fatigue in relapsing-remitting multiple sclerosis. PLoS One. 2018;13(10):e0201162. Epub 20181024. doi: 10.1371/journal.pone.0201162. PubMed PMID: 30356315; PubMed Central PMCID: PMCPMC6200185.
43. Svolgaard O, Andersen KW, Bauer C, Madsen KH, Blinkenberg M, Sellebjerg F, et al. Mapping grip-force related brain activity after a fatiguing motor task in multiple sclerosis. Neuroimage-Clinical. 2022;36:11. doi: 10.1016/j.nicl.2022.103147. PubMed PMID: WOS:000889292900001.
44. Téllez N, Alonso J, Río J, Tintoré M, Nos C, Montalban X, et al. The basal ganglia: a substrate for fatigue in multiple sclerosis. Neuroradiology. 2008;50(1):17-23. Epub 20071023. doi: 10.1007/s00234-007-0304-3. PubMed PMID: 17955232.
45. Tomasevic L, Zito G, Pasqualetti P, Filippi M, Landi D, Ghazaryan A, et al. Cortico-muscular coherence as an index of fatigue in multiple sclerosis. Mult Scler. 2013;19(3):334-43. Epub 20120703. doi: 10.1177/1352458512452921. PubMed PMID: 22760098.
46. Wilting J, Rolfsnes HO, Zimmermann H, Behrens M, Fleischer V, Zipp F, et al. Structural correlates for fatigue in early relapsing remitting multiple sclerosis. Eur Radiol. 2016;26(2):515-23. Epub 20150531. doi: 10.1007/s00330-015-3857-2. PubMed PMID: 26026721.
47. Wu L, Zhang Y, Zhou FQ, Gao L, He LC, Zeng XJ, et al. Altered intra- and interregional synchronization in relapsing-remitting multiple sclerosis: a resting-state fMRI study. Neuropsychiatric Disease and Treatment. 2016;12:853-62. doi: 10.2147/ndt.S98962. PubMed PMID: WOS:000374146300001.
48. Wu L, Huang M, Zhou F, Zeng X, Gong H. Distributed causality in resting-state network connectivity in the acute and remitting phases of RRMS. BMC Neuroscience. 2020;21(1). doi: 10.1186/s12868-020-00590-4.
49. Yaldizli Ö, Penner IK, Yonekawa T, Naegelin Y, Kuhle J, Pardini M, et al. The association between olfactory bulb volume, cognitive dysfunction, physical disability and depression in multiple sclerosis. Eur J Neurol. 2016;23(3):510-9. Epub 20151119. doi: 10.1111/ene.12891. PubMed PMID: 26699999.
50. Zellini F, Niepel G, Tench CR, Constantinescu CS. Hypothalamic involvement assessed by T1 relaxation time in patients with relapsing-remitting multiple sclerosis. Mult Scler. 2009;15(12):1442-9. Epub 20091207. doi: 10.1177/1352458509350306. PubMed PMID: 19995847.
51. Zhou F, Zhuang Y, Gong H, Wang B, Wang X, Chen Q, et al. Altered inter-subregion connectivity of the default mode network in relapsing remitting multiple sclerosis: A functional and structural connectivity study. PLoS ONE. 2014;9(7). doi: 10.1371/journal.pone.0101198.
52. Zhou F, Gong H, Chen Q, Wang B, Peng Y, Zhuang Y, et al. Intrinsic Functional Plasticity of the Thalamocortical System in Minimally Disabled Patients with Relapsing-Remitting Multiple Sclerosis. Front Hum Neurosci. 2016;10:2. Epub 20160125. doi: 10.3389/fnhum.2016.00002. PubMed PMID: 26834600; PubMed Central PMCID: PMCPMC4725198.
