# Supplementary material for: Brain connectivity changes underlying depression and fatigue in relapsing-remitting multiple sclerosis: A systematic review
Source: PLoS One. 2024 Mar 29;19(3):e0299634. doi: 10.1371/journal.pone.0299634 (PMC10980255; doi:10.1371/journal.pone.0299634)
Supplement: S6 Table — (PDF) [file pone.0299634.s009.pdf]

S6 Table. Full breakdown of Institute of Health Economics (IHE) checklist scores [1]. Y=Yes; N=No; ?=Unclear; P=Partial; Y*=retrospective study, same points as a ‘yes’ answer.

	Study objective	Study design			Study population			Outcome measure		Statistical analysis	Results and conclusions				Competing interests and sources of support	
Paper	Was the hypothesis/aim/objective of the study clearly stated?	Was the study conducted prospectively?	Were the cases collected in more than one center?	Were patients recruited consecutively?	Were the characteristics of the patients included in the study described?	Were the eligibility criteria (i.e. inclusion and exclusion criteria) for entry into the study clearly stated? Were the eligibility criteria (i.e. inclusion and exclusion criteria) for entry into the study clearly stated?	Did patients enter the study at a similar point in the disease?	Were relevant outcome measures established a priori?	Were the relevant outcomes measured using appropriate objective/ subjective methods?	Were the statistical tests used to assess the relevant outcomes appropriate?	Was follow-up long enough for important events and outcomes to occur?	Were losses to follow-up reported?	Did the study provided estimates of random variability in the data analysis of relevant outcomes?	Were the conclusions of the study supported by the results?	Were both competing interests and sources of support for the study reported?	Total
Calabrese et al. [2]	Y	Y	N	Y	Y	P	N	Y	y	y	Y	N	Y	y	y	10.5 (70%)
Cavallari et al. [3]	Y	Y	?	?	Y	Y	N	Y	Y	Y	Y	Y*	Y	Y	Y	11 (73%)
Hildebrandt and Eling [4]	P	Y	?	?	Y	Y	N	Y	Y	?	Y	N	Y	?	Y	7.5 (50%)
Yaldizli et al. [5]	Y	Y	N	Y	Y	Y	N	Y	Y	Y	Y	N	Y	Y	Y	11 (73%)
Yarraguntla et al. [6]	Y	Y	N	?	Y	Y	Y	Y	Y	Y	Y	N	Y	Y	Y	11 (73%)
Yarraguntla et al. [7]	Y	N	N	?	Y	Y	N	Y	y	Y	Y	Y*	P	Y	N	11.5 (77%)
Gilio et al. [8]	Y	N	N	Y	Y	Y	Y	?	Y	Y	Y	Y*	P	Y	Y	10.5 (70%)
Tijhuis et al. [9]	Y	Y	?	?	Y	Y	N	Y	Y	Y	Y	Y	Y	Y	Y	11 (73%)

References:

1. (IHE) IoHE. Institute of Health Economics (IHE). Quality Appraisal of Case Series Studies Checklist. : Edmonton (AB); 2014 [cited 2023 06-03-2023]. Available from: http://www.ihe.ca/research-programs/rmd/cssqac/cssqac-about.
2. Calabrese M, Rinaldi F, Grossi P, Mattisi I, Bernardi V, Favaretto A, et al. Basal ganglia and frontal/parietal cortical atrophy is associated with fatigue in relapsing-remitting multiple sclerosis. Mult Scler. 2010;16(10):1220-8. Epub 20100729. doi: 10.1177/1352458510376405. PubMed PMID: 20670981.
3. Cavallari M, Palotai M, Glanz BI, Egorova S, Prieto JC, Healy BC, et al. Fatigue predicts disease worsening in relapsing-remitting multiple sclerosis patients. Multiple Sclerosis Journal. 2016;22(14):1841-9. doi: 10.1177/1352458516635874. PubMed PMID: WOS:000390576600013.
4. Hildebrandt H, Eling P. A longitudinal study on fatigue, depression, and their relation to neurocognition in multiple sclerosis. J Clin Exp Neuropsychol. 2014;36(4):410-7. Epub 20140407. doi: 10.1080/13803395.2014.903900. PubMed PMID: 24702275.
5. Yaldizli Ö, Glassl S, Sturm D, Papadopoulou A, Gass A, Tettenborn B, et al. Fatigue and progression of corpus callosum atrophy in multiple sclerosis. J Neurol. 2011;258(12):2199-205. Epub 20110519. doi: 10.1007/s00415-011-6091-0. PubMed PMID: 21594686.
6. Yarraguntla K, Bao F, Lichtman-Mikol S, Razmjou S, Santiago-Martinez C, Seraji-Bozorgzad N, et al. Characterizing Fatigue-Related White Matter Changes in MS: A Proton Magnetic Resonance Spectroscopy Study. Brain Sci. 2019;9(5). Epub 20190527. doi: 10.3390/brainsci9050122. PubMed PMID: 31137831; PubMed Central PMCID: PMCPMC6562940.
7. Yarraguntla K, Seraji-Bozorgzad N, Lichtman-Mikol S, Razmjou S, Bao F, Sriwastava S, et al. Multiple Sclerosis Fatigue: A Longitudinal Structural MRI and Diffusion Tensor Imaging Study. J Neuroimaging. 2018;28(6):650-5. Epub 20180723. doi: 10.1111/jon.12548. PubMed PMID: 30039613.
8. Gilio L, Buttari F, Pavone L, Iezzi E, Galifi G, Dolcetti E, et al. Fatigue in Multiple Sclerosis Is Associated with Reduced Expression of Interleukin-10 and Worse Prospective Disease Activity. Biomedicines. 2022;10(9):13. doi: 10.3390/biomedicines10092058. PubMed PMID: WOS:000858486900001.
9. Tijhuis FB, Broeders TAA, Santos FAN, Schoonheim MM, Killestein J, Leurs CE, et al. Dynamic functional connectivity as a neural correlate of fatigue in multiple sclerosis. Neuroimage-Clinical. 2021;29:9. doi: 10.1016/j.nicl.2020.102556. PubMed PMID: WOS:000620121700041.
