# Supplementary material for: Brain connectivity changes underlying depression and fatigue in relapsing-remitting multiple sclerosis: A systematic review
Source: PLoS One. 2024 Mar 29;19(3):e0299634. doi: 10.1371/journal.pone.0299634 (PMC10980255; doi:10.1371/journal.pone.0299634)
Supplement: S7 Table — (PDF) [file pone.0299634.s010.pdf]

S7 Table. Overview of study details for publications included (N=60) in the current systematic review.

Authors	MRI sequence	Field Strength	Design	Participants (n)	Sex: no. female (%)	Country	Source of participants
Alshehri et al. [1]	T1W, FLAIR, DTI	3T	Cross-sectional	RRMS: 37; HC: 19;<br>EDSS ≤ 4.0 (1.9±0.15)	RRMS 37(78%)<br>HC 19(80%)	Australia	Patients
Altermatt et al. [2]	T1W, T2W, FLAIR	1T/1.5T/3T	Retrospective	RRMS (1907)<br>Mean age: 39<br>EDSS: 2	RRMS 1362 (71%)	Multicentre, mostly the United States)	All available data from patients participating in the multicentre FREEDOMS (N = 1272) and FREEDOMS II (n = 1083) phase 3 clinical trials
Andreasen et al. [3]	T1W, T2W, FLAIR, DTI, MRS proton spectroscopy	3T	Cross-sectional	RRMS (34): F (17), nF (17)<br>HC (7)<br>Mean age: 43 (F), 39 (nF), 39 (HC)<br>EDSS: 1-3.5	F 12(71%)<br>nF 8(47%)<br>HC 6(86%)	Denmark	Patients
Bauer et al. [4]	T1W, T2W, FLAIR, DWI (dMRI)	3T	Cross-sectional	RRMS (46); HC (25);<br>Mean age(range)[DS]: RRMS: 36.1(22–53)[8.6]; HC: 35.8 (19–55)[10.6]; EDSS Mean 1.5(0–3.5)[1.3]	MS: 33 (72%)<br>HC: 16(64%)	Denmark	Patients
Beaudoin et al. [5]	T1W, T2W, HARDI	3T	Cross-sectional	RRMS:24; HC: 11;<br>EDSS(median[range]): 1.5[0-3]	RRMS 20(83%)<br>HC: 8(73%)	Canada	Patients
Benesova et al. [6]	T1W, T2W	1.5T	Cross-sectional	RRMS (20): D (10), nD (10);<br>EDSS: 1-4<br>Mean age: 37.3	RRMS 14(70%)	Czech Republic	Patients
Bisecco et al. [7]	T1W, T2W, DTI	3T	Cross sectional	RRMS (60): F (30), nF (30), HC (29);<br>Mean age: 40.2 (nF), 40.7 (F)<br>EDSS: 2(F), 1.5 (nF)	RRMS 41(68%)<br>nF 21(70%)<br>F 20(67%)<br>HC 16(55%)	Italy	Patients. HC were recruited from a large HC database.
Calabrese et al. [8]	FLAIR, T2W	1.5T	Longitudinal	RRMS (152)<br>HC (42)<br>Mean age: 34 (RRMS), 35.5 (HC)<br>EDSS: 2.7	RRMS 95(63%)<br>HC 26(62%)	Italy	Patients
Carotenuto et al. [9]	T1, T2, FLAIR, rs-fMRI	3T	Cross-sectional	RRMS (29), HC (24)<br>Mean age: 42 (RRMS), 38 (HC)<br>Mean EDSS: 3.2	RRMS 17(59%)<br>HC 15(63%)	UK	Patients
Cavallari et al. [10]	T2W	1.5T	Retrospective	RRMS (66)<br>Mean age: 48<br>EDSS: 1.5	RRMS 52(79%)<br>Converters* 26(79%)<br>Non-converters 26(79%)	USA	Retrospectively selected from a larger cohort of over 800 prospectively followed MS patients within the CLIMB study
Codella et al. [11]	DE TSE, 2D GE, pulsed gradient spin-echo echo-planar	1.5T	Cross-sectional	RRMS (28): F (14), nF (14)<br>HC (30)<br>Mean age: 37.6 (nF), 39.1 (F)<br>Median EDSS: 1	RRMS 19(68%)<br>HC 18(60%)	Italy	Patients
Damasceno et al. [12]	FLAIR, T1W, T2W	3T	Cross-sectional	RRMS (49)<br>HC (30)<br>Mean age: 30.94 (RRMS), 29.52 (HC)	RRMS 38 (77.6%)<br>HC 23 (76.7%)	Brazil	Patients

				Median EDSS: 2			
Filippi et al. [13]	T1W, fMRI, DE TSE	1.5T	Cross-sectional	RRMS (29): F (15), nF (14), HC (15);<br>Mean age: 39.3 (F), 37.6 (nF)<br>Median EDSS: 1	MS 20(69%)<br>HC 9(60%)	Italy	Patients
Finke et al. [14]	T2W, DTI, T1, FLAIR, rs-fMRI	3T	Cross-sectional	RRMS (44)<br>HC (20)<br>Mean age: 45.9 (RRMS), 43.1 (HC)	MS 26(59%)<br>HC 11(55%)	Germany	Patients
Gilio et al. [15]	T1W, FLAIR, PD, FLAIR, Gd+ T1W	3T	Longitudinal	RRMS: 106 (35 had MRI);<br>EDSS(median[IQR]): 1.5 [1–2.125]	RRMS 106 (66%)	Italy	patients
Gold et al. [16]	T1W, T2W, FLAIR	3T	Cross-sectional	RRMS (29): D (21), nD (8)<br>HC (20)<br>Mean age: 37.5<br>Mean EDSS: 2.5	RRMS 25(86%)<br>HC 18(90%)	USA	Patients
Golde et al. [17]	BOLD, MP-RAGE, DTI	3T	Cross-sectional	RRMS (30), HC (34);<br>Age: RRMS 40.20±9.87; HC 39.57±8.36;<br>EDSS: 1.75 (0–4)	RRMS: 18(60%);<br>HC: 19(63%)	Germany	Patients
Gómez et at. [18]	T1W, T2*W	1.5T	Cross-sectional	RRMS (60), nF (28), F(32);<br>HC (18);<br>Mean age(SD)[range]:<br>HC 31.06(5.67)[22-44];<br>nF: 34.96(5.87)[20-44];<br>F: 37.72(5.90)[22-47];<br>EDSS mean(SD)[range]<br>nF: 1.96(1.20)[0-5];<br>F 3.20(1.68)[1-6]	HC: 8(44%);<br>nF: 18(64%);<br>F: 21(66%)	Spain	Patients
Hassan et al. [19]	T1W, T2W, FLAIR, DWI	1.5T	Cross-sectional	D-RRMS (20); nD-MS (10);<br>HC (10);<br>Mean age (range): 27 (21-36);<br>EDSS ≤ 5	MS: 24(80%)	Egypt	Patients
Hildebrandt et al. [20]	T1W	1.5T	Cross-sectional	RRMS (45)<br>Mean age: 38.9<br>Median EDSS: 2.6	RRMS 29(64%)	Germany	Patients
Hildebrandth and Eling [21]	T1W	1.5T	Longitudinal	40 RRMS: no increase in F (23), increase in F (17);<br>Mean age: 38.5 (CIF), 37.9 (NCIF)<br>EDSS: 2.3 (CIF), 3.2 (NCIF)	No increase in F 17(74%)<br>Increase in F (9(53%)	Germany	Patients
Huang et al. [22]	T1W, T2W, FLAIR, rs-fMRI	3T	Cross-sectional	RRMS (33)<br>HC (33)<br>Mean age: 41.8 (RRMS), 42.2 (HC)<br>EDSS: 1.97	RRMS 21(64%)<br>HC 21(64%)	China	Patients
Iancheva et al. [23]	T1, fMRI	3T	Cross sectional	RRMS (29): F (15), nF (14)<br>Mean age: 40.7 (F), 36.9 (nF)<br>Median EDSS:1	No data	Bulgaria	Patients

Jaeger et al. [24]	T1W, T2W, rs-fMRI	3T	Cross sectional	RRMS (70): F (39), nF (38)<br>Median age: 40 (F), 34.5 (nF), 36 (HC)<br>Median EDSS: 2.5 (F), 2 (nF)	F 32(82%)<br>nF 24(63%)<br>HC 26(63%)	Germany	Recruited from ongoing prospective studies
Kever et al. [25]	T1W, FLAIR, T2W	3T	Longitudinal	RRMS: 51;<br>EDSS(median(IQR)): 1.5 (4.5)	RRMS (80.4%)	USA	MEM CONNECT cohort
Khedr et al. [26]	PD, FLAIR, T1W, T2W, Gd+T1W	1.5T	Cross-sectional	43 RRMS patients with 40 ;<br>EDSS (mean±SD) F: 4.13±1.59;<br>nF: 2.42±1.17	RRMS:<br>F 24(77%)<br>nF 6(50%)	Egypt	Patients
Kopchak & Odintsova [27]	?	1.5T	Cross-sectional	RRMS: 106; A group: under 40 years (n=48); B group: —≥40 (n=58); EDSS score: 6.5—8 points (EXCLUSION)	RRMS 81(76%)	Ukraine	Patients
Lazzarotto et al. [28]	T1W, FLAIR, DIR	3T	Cross-sectional	RRMS (61)<br>HC (56)<br>Mean age:<br>37.9 (RRMS),<br>35.2 (HC)<br>EDSS: 1.77	RRMS 43(70%)<br>HC 34(61%)	Italy	Retrospective: Data were retrieved from the MRI and clinical databases of the MS center of Padua, screening all patients that came to our attention from April 2014 to May 2018
Morgante et al. [29]	T1 GD+, T2W, transcranial magnetic stimulation	1.5T	Cross sectional	RRMS (33): F (16), nF (17)<br>HC (12)<br>Mean age: 41 (F), 38 (nF),<br>EDSS: 1.8 (F), 1.6 (nF)	nF 13(77%)<br>F 9(56%)	Italy	Patients
Niepel et al. [30]	3D FLASH (for T1maps), T2	1.5T	Cross-sectional	RRMS (52[48 for FSS]; 34 for MRI, clinical): F (39[20 for MRI]), nF (23[11 for MRI]). HC (19)<br>Mean age: 39 (F), 33 (nF)<br>Median EDSS: 3 (F), 2 (nF)	RRMS 40(77%)<br>[37(77%) for FSS; 27(79%) for MRI, clinical]<br>HC 14(74%)	UK	Patients
Nigro et al. [31]	T1W, FLAIR, DTI	3T	Cross-sectional	RRMS (42): D (20), nD (22). HC (16)<br>Mean age: 37.1 (D), 31.5 (nD), 35.3 (HC)<br>EDSS: 2.3(nD), 2.8(D)	D (85%)<br>nD (60%)<br>HC (43%)	Italy	Patients
Nygaard et al. [32]	T1W, FLAIR	1.5T	Cross-sectional	RRMS (61)<br>HC (61);<br>EDSS: 0-4;<br>Mean age: RRMS 34.2, HC 33.5	RRMS 47(77%)<br>HC 47(77%)	Norway	Patients
Pardini et al. [33]	T1W, T2W, DTI	1.5T	Cross-sectional	RRMS (40): F (15), nF (25), HC (15);<br>Mean age: 41 (F), 36 (nF)<br>Mean EDSS: 1.6	RRMS 28(70%)<br>HC no data	Italy	Patients
Pardini et al. [34]	T1W, T2W, DTI	1.5T	Cross-sectional	RRMS (77): F (25), nF (52)<br>Mean age: 40.8<br>EDSS: 2	No data	Italy	Patients<br>(Reporting fatigue)
Pokryszko-Dragan et al. [35]	T1W, T2W, FLAIR, DWI, DTI, 3D-FSPGR GD+	1.5T	Cross-sectional	RRMS (50)<br>HC (27)<br>Mean age: 36.4 (RRMS), 36.3 (HC)<br>EDSS: 2.7	RRMS 37(74%)<br>HC 19(70%)	Poland	Patients

Pravatà et al. [36]	T1W, T2W, rs-fMRI	3T	Cross-sectional	RRMS (22): F (11), nF (11)<br>HC (12)<br>Mean age: 46.6 (F), 40 (nF), 41.4 (HC)<br>EDSS: 1.5 (nF), 2.5 (F)	HC 6(50%)<br>nF 4(57%)<br>F 4(57%)	Switzerland	Patients
Riccelli et al. [37]	T1W, FLAIR, fMRI	3T	Cross sectional	RRMS (77),<br>HC (20)<br>Mean age: 34 (RRMS), 36.4 (HC)<br>Median EDSS: 2	RRMS 46(60%)<br>HC 10(50%)	UK	Patients
Rocca et al. [38]	T2W, T1W, fMRI	1.5T	Cross sectional	RRMS (24): F (11), nF (13)<br>HC (46)<br>Mean age: 33.8 (F), 31.2 (nF)<br>Median EDSS: 1 (both groups)	RRMS 22(92%)	Italy	Patients
Rocca et al. [39]	T2W, T1W, fMRI	3T	Cross-sectional	RRMS (79): F (50), nF (29)<br>HC (26)<br>Mean age: 42.6 (F), 40 (nF), 39.2 (HC)<br>Median EDSS: 2 (F), 1.5 (nF)	HC 17(65%)<br>nF-MS 19(66%)<br>F-MS 33(66%)	Italy	Patients
Rojas et al. [40]	T1W, T2W, FLAIR, DTI	1.5T	Cross-sectional	RRMS (45): D (23), nD (22);<br>Mean age: 33.5 (D), 32.5 (nD), 32.3 (HC)<br>EDSS: 1.6 (nD), 2 (D)	HC 14(70%)<br>nD 14(63.6%)<br>D 19(82.6%)	Argentina	Patients
Romanello et al. [41]	T2W, rs-fMRI	3T	Cross-sectional	RRMS: 101; HC: 101;<br>EDSS (median(IQR)) RRMS 1.5 (1.5);<br>(EDSS ≤ 1, n = 36) 1 (1); (EDSS ≥ 2, n = 39) 2.5 (1)	RRMS 67(66%)<br>(EDSS ≤ 1) 20(56%);<br>(EDSS ≥ 2) 27(69%); HC 67(66%)	Germany	Patients
Ruiz-Rizzo et al. [42]	T1W, T2*W, FLAIR, BOLD fMRI	3T	Cross-sectional	RRMS: 104;<br>EDSS<3: 61 (58.7%); 3 – 7: 43 (41.3%)	RRMS 66(63.5%)	Germany	Patients
Saberi et al. [43]	T1W	1.5T	Cross sectional	RRMS (43)<br>EDSS ≤ 6<br>Age 21-59	RRMS 32 (74%)	Iran	Recruited from the Cross-Modal Research Initiative for MS and Optic Neuritis (CRIMSON)
Specogna et al. [44]	T1W, T2W, T1 GD+, FLAIR, fMRI: finger against thumb tapping	1.5T	Cross sectional	RRMS (24): F (12), nF (12)<br>HC (15)<br>Mean age: 39.8 (F), 38.7 (nF)<br>EDSS: 1.5	RRMS 20(83%)	Italy	Patients
Štecková et al. [45]	T1W	1.5T	Cross-sectional	RRMS (43)<br>HC (19)<br>Mean age: 35.2 (MS5), 43.5 (MS10)<br>EDSS: not stated	RRMS 29(67%)<br>CIS 12(63%)<br>MS5 11(73%)<br>MS10 6(67%)	Czech Republic	Patients
Svolgaard et al. [46]	T1W, T2W, FLAIR, fMRI	3T	Cross-sectional	RRMS (24): F (12), nF (12)<br>HC (15)<br>Mean age: 40.9 (F), 38.7 (nF)<br>EDSS: 1.5	MS 30(68%)<br>HC 16(64%)	Germany	No information
Svolgaard et al. [47]	T1W, T2W, FLAIR, fMRI	3T	Cross-sectional	RRMS: 44; HC: 25;<br>EDSS score ≤ 3.5	RRMS 30(68%)<br>HC 16(64%)	Denmark	Patients

Tellez et al. [48]	T2, proton magnetic resonance	1.5T	Cross sectional	RRMS (30): F (17), nF (13)<br>HC (21)<br>Mean age: 38.5 (F), 37.8 (nF)<br>Median EDSS: 2.5 (F), 1.5 (nF)	F 13(76.4%)<br>nF 10(78.1%)	Spain	Patients
Tijhuis et al. [49]	T1W, FLAIR, fMRI	3T	Longitudinal	RRMS: 35; HC: 19;<br>EDSS Baseline 3(1–6)<br>EDSS Follow up 3(1.50–7)	RRMS 20(57%)<br>HC 11(58%)	The Netherlands	Patients
Tomasevic et al. [50]	T1 GD+, T2, T1 FLAIR	1.5T	Cross-sectional	RRMS (20): F (11), nF (9)<br>Mean age: 38.5 (F); 35.9 (nF)<br>EDSS: 0-2	RRMS 13(65%)<br>nF 6(67%)<br>F 8(73%)	Italy	Patients
Wilting et al. [51]	T1W, FLAIR, DTI	3T	Cross-sectional	RRMS (79): F (38), nF (41)<br>Median age: 34.5 (F), 30 (nF)<br>Median EDSS: 2	nF 26(63%)<br>F 30(79%)<br>HC 18(45%)	Germany	Patients
Wu et al. [52]	T1W, T2W, rs-fMRI	3T	Cross-sectional	RRMS (22)<br>HC (22)<br>Mean age: 44.6 (RRMS), 40.1 (HC)<br>EDSS: 1.8	RRMS 13(59%)<br>HC 13(59%)	China	Patients
Wu et al. [53]	T2*W, T2W, T1W, rs-fMRI	3T	Cross-sectional	RRMS (41), HC (23);<br>Mean age(range): acute RRMS: 43.1 (15–61), remitting RRMS: 40.7 (21–66), HC 40.7 (26–58);<br>Mean EDSS (range):<br>acute RRMS 2.8 (1.5–4);<br>remitting RRMS 2.0 (0–3.5)	RRMS:<br>acute: 10(59%);<br>remitting 15(63%); HC 11(49%);	China	Patients
Yaldizli et al. [54]	T1 GD+, T1W, T2W, FLAIR	1.5T	Retrospective	RRMS (70): F (28), nF (42). HC (27)<br>Mean age: 40.4 (RRMS), 43.7 (HC)<br>EDSS: 2.8	Total RRMS 61(67.1%)<br>FSS<4 37(88.1%)<br>FSS≥24(85.7%)	Switzerland	Patients
Yaldizli et al. [55]	T1W, PD/T2W	1.5T	Cross-sectional	RRMS (103)<br>Mean age: 49<br>Median EDSS: 3	RRMS 96(65.8%)<br>RRMS 73(70.9%)<br>SPMS 18(51.4%)<br>PPMS 5(62.5%)<br>HC 17(63%)	Switzerland	Recruited from an ongoing prospective, non-interventional cohort study on the phenotype-genotype characterization of MS
Yarraguntla et al. [56]	T1W, DTI	3T	Retrospective longitudinal	RRMS (43): HF (15), MF (14), LF (14)<br>Mean age: 43 (HF), 39 (MF), 39 (LF)<br>EDSS: 3.6 (HF), 2.7 (MF), 2.35 LF)	No data	USA?	Retrospective longitudinal study from the Wayne State University School of Medicine MS Center
Yarraguntla et al. [57]	T2, FLAIR, MR spectroscopy	3T	Longitudinal, 1 year	RRMS (48): HF (16), MF (18), LF (14)<br>Mean age: 43 (HF), 39 (MF), 39 (LF)<br>Median EDSS: 3 (HF), 2.71 (MF), 2.42 (LF)	RRMS 33(69%)<br>HF 12(75%)<br>MF 14(78%)<br>LF 7(50%)	USA	Patients
Zellini et al. [58]	T1W, T2W	1.5T	Cross sectional	RRMS (44[40 for F tests, 36 intermediate count, 32 final count),<br>HC (13)<br>Median age: 39<br>Median EDSS: 2.5	RRMS 35(80%)<br>[29(81%)]<br>HC 9(69%)	UK	Patients

Zhou et al. [59]	T2W, T1W, DTI, rs-fMRI	3T	Cross-sectional	RRMS (24)<br>HC (24)	RRMS: 16(67%)<br>HC: 16(67%)	China	Patients
Zhou et al. [60]	T1W, T2W, DTI, rs-fMRI, FLAIR	3T	Cross sectional	RRMS (20)<br>HC (20)<br>Mean age: 39.4 (RRMS), 38.1 (HC)<br>EDSS: 1.67	RRMS 15(75%)<br>HC 15(75%)	China	Patients
BOLD: Blood-Oxygen Level Dependent, D/nD: [not]depression/depressed, DTI: Diffusion Tensor Imaging, EDSS: Expanded Disability Status Scale, F/nF: [not]fatigue/fatigued, FA: Fractional Anisotropy, FLAIR: Fluid-attenuated Inversion Recovery, fMRI: functional Magnetic Resonance Imaging, RRMS: Relapsing-remitting multiple sclerosis, rs-fMRI: Resting-state Functional Magnetic Resonance Imaging, SD: Standard Deviation, HC: healthy controls, GD+: Gadolinium Enhancing, SPMS: Secondary progressive MS, PPMS: Primary progressive MS, LF/MF/HF: low/medium/high fatigue, FSPGR: fast spoiled gradient echo, DLPFC: dorsolateral prefrontal cortex. *Converters: converted to confirmed (⩾2 years) EDSS score ⩾3 within a follow-up period ⩾3 years.							

References:

1. Alshehri A, Al-iedani O, Arm J, Gholizadeh N, Billiet T, Lea R, et al. Neural diffusion tensor imaging metrics correlate with clinical measures in people with relapsing-remitting MS. Neuroradiology Journal. 2022;35(5):592-9. doi: 10.1177/19714009211067400.
2. Altermatt A, Gaetano L, Magon S, Häring DA, Tomic D, Wuerfel J, et al. Clinical Correlations of Brain Lesion Location in Multiple Sclerosis: Voxel-Based Analysis of a Large Clinical Trial Dataset. Brain Topogr. 2018;31(5):886-94. Epub 20180529. doi: 10.1007/s10548-018-0652-9. PubMed PMID: 29845492.
3. Andreasen AK, Jakobsen J, Soerensen L, Andersen H, Petersen T, Bjarkam CR, et al. Regional brain atrophy in primary fatigued patients with multiple sclerosis. Neuroimage. 2010;50(2):608-15. Epub 20100106. doi: 10.1016/j.neuroimage.2009.12.118. PubMed PMID: 20060048.
4. Bauer C, Dyrby TB, Sellebjerg F, Madsen KS, Svolgaard O, Blinkenberg M, et al. Motor fatigue is associated with asymmetric connectivity properties of the corticospinal tract in multiple sclerosis. Neuroimage-Clinical. 2020;28. doi: 10.1016/j.nicl.2020.102393. PubMed PMID: WOS:000600619100036.
5. Beaudoin AM, Rheault F, Theaud G, Laberge F, Whittingstall K, Lamontagne A, et al. Modern Technology in Multi-Shell Diffusion MRI Reveals Diffuse White Matter Changes in Young Adults With Relapsing-Remitting Multiple Sclerosis. Frontiers in Neuroscience. 2021;15:13. doi: 10.3389/fnins.2021.665017. PubMed PMID: WOS:000687832800001.
6. Benesova Y, Niedermayerova I, Mechl M, Havlikova P. The relation between brain MRI lesions and depressive symptoms in multiple sclerosis. Bratisl Lek Listy. 2003;104(4-5):174-6. PubMed PMID: 14604264.
7. Bisecco A, Caiazzo G, d'Ambrosio A, Sacco R, Bonavita S, Docimo R, et al. Fatigue in multiple sclerosis: The contribution of occult white matter damage. Mult Scler. 2016;22(13):1676-84. Epub 20160204. doi: 10.1177/1352458516628331. PubMed PMID: 26846989.
8. Calabrese M, Rinaldi F, Grossi P, Mattisi I, Bernardi V, Favaretto A, et al. Basal ganglia and frontal/parietal cortical atrophy is associated with fatigue in relapsing-remitting multiple sclerosis. Mult Scler. 2010;16(10):1220-8. Epub 20100729. doi: 10.1177/1352458510376405. PubMed PMID: 20670981.
9. Carotenuto A, Wilson H, Giordano B, Caminiti SP, Chappell Z, Williams SCR, et al. Impaired connectivity within neuromodulatory networks in multiple sclerosis and clinical implications. J Neurol. 2020;267(7):2042-53. Epub 20200326. doi: 10.1007/s00415-020-09806-3. PubMed PMID: 32219555; PubMed Central PMCID: PMCPMC7320961.

10. Cavallari M, Palotai M, Glanz BI, Egorova S, Prieto JC, Healy BC, et al. Fatigue predicts disease worsening in relapsing-remitting multiple sclerosis patients. Multiple Sclerosis Journal. 2016;22(14):1841-9. doi: 10.1177/1352458516635874. PubMed PMID: WOS:000390576600013.
11. Codella M, Rocca MA, Colombo B, Martinelli-Boneschi F, Comi G, Filippi M. Cerebral grey matter pathology and fatigue in patients with multiple sclerosis: a preliminary study. J Neurol Sci. 2002;194(1):71-4. doi: 10.1016/s0022-510x(01)00682-7. PubMed PMID: 11809169.
12. Damasceno A, Damasceno BP, Cendes F. Atrophy of reward-related striatal structures in fatigued MS patients is independent of physical disability. Mult Scler. 2016;22(6):822-9. Epub 20150803. doi: 10.1177/1352458515599451. PubMed PMID: 26238465.
13. Filippi M, Rocca MA, Colombo B, Falini A, Codella M, Scotti G, et al. Functional magnetic resonance imaging correlates of fatigue in multiple sclerosis. Neuroimage. 2002;15(3):559-67. doi: 10.1006/nimg.2001.1011. PubMed PMID: 11848698.
14. Finke C, Schlichting J, Papazoglou S, Scheel M, Freing A, Soemmer C, et al. Altered basal ganglia functional connectivity in multiple sclerosis patients with fatigue. Mult Scler. 2015;21(7):925-34. Epub 20141112. doi: 10.1177/1352458514555784. PubMed PMID: 25392321.
15. Gilio L, Buttari F, Pavone L, Iezzi E, Galifi G, Dolcetti E, et al. Fatigue in Multiple Sclerosis Is Associated with Reduced Expression of Interleukin-10 and Worse Prospective Disease Activity. Biomedicines. 2022;10(9):13. doi: 10.3390/biomedicines10092058. PubMed PMID: WOS:000858486900001.
16. Gold SM, Kern KC, O'Connor MF, Montag MJ, Kim A, Yoo YS, et al. Smaller cornu ammonis 2-3/dentate gyrus volumes and elevated cortisol in multiple sclerosis patients with depressive symptoms. Biol Psychiatry. 2010;68(6):553-9. Epub 20100619. doi: 10.1016/j.biopsych.2010.04.025. PubMed PMID: 20646680; PubMed Central PMCID: PMCPMC3122328.
17. Golde S, Heine J, Pöttgen J, Mantwill M, Lau S, Wingenfeld K, et al. Distinct Functional Connectivity Signatures of Impaired Social Cognition in Multiple Sclerosis. Frontiers in Neurology. 2020;11. doi: 10.3389/fneur.2020.00507.
18. Cruz Gomez AJ, Campos NV, Belenguer A, Avila C, Forn C. Regional Brain Atrophy and Functional Connectivity Changes Related to Fatigue in Multiple Sclerosis. Plos One. 2013;8(10). doi: 10.1371/journal.pone.0077914. PubMed PMID: WOS:000326034500049.
19. Hassan TA, Elkholy SF, Mahmoud BE, ElSherbiny M. Multiple sclerosis and depressive manifestations: can diffusion tensor MR imaging help in the detection of microstructural white matter changes? Egyptian Journal of Radiology and Nuclear Medicine. 2019;50(1). doi: 10.1186/s43055-019-0033-8. PubMed PMID: WOS:000486165900001.
20. Hildebrandt H, Hahn HK, Kraus JA, Schulte-Herbrüggen A, Schwarze B, Schwendemann G. Memory performance in multiple sclerosis patients correlates with central brain atrophy. Mult Scler. 2006;12(4):428-36. doi: 10.1191/1352458506ms1286oa. PubMed PMID: 16900756.
21. Hildebrandt H, Eling P. A longitudinal study on fatigue, depression, and their relation to neurocognition in multiple sclerosis. J Clin Exp Neuropsychol. 2014;36(4):410-7. Epub 20140407. doi: 10.1080/13803395.2014.903900. PubMed PMID: 24702275.
22. Huang M, Zhou F, Wu L, Wang B, Wan H, Li F, et al. Synchronization within, and interactions between, the default mode and dorsal attention networks in relapsing-remitting multiple sclerosis. Neuropsychiatr Dis Treat. 2018;14:1241-52. Epub 20180514. doi: 10.2147/ndt.S155478. PubMed PMID: 29795982; PubMed Central PMCID: PMCPMC5957478.
23. Iancheva D, Trenova A, Mantarovau S, Terziyski K. Functional Magnetic Resonance Imaging Correlations Between Fatigue and Cognitive Performance in Patients With Relapsing Remitting Multiple Sclerosis. Frontiers in Psychiatry. 2019;10. doi: 10.3389/fpsyt.2019.00754. PubMed PMID: WOS:000496145700001.

24. Jaeger S, Paul F, Scheel M, Brandt A, Heine J, Pach D, et al. Multiple sclerosis-related fatigue: Altered resting-state functional connectivity of the ventral striatum and dorsolateral prefrontal cortex. Mult Scler. 2019;25(4):554-64. Epub 20180221. doi: 10.1177/1352458518758911. PubMed PMID: 29464981.
25. Kever A, Buyukturkoglu K, Levin SN, Riley CS, De Jager P, Leavitt VM. Associations of social network structure with cognition and amygdala volume in multiple sclerosis: An exploratory investigation. Multiple Sclerosis Journal. 2022;28(2):228-36. doi: 10.1177/13524585211018349. PubMed PMID: WOS:000656009600001.
26. Khedr EM, Desoky T, Gamea A, Ezzeldin MY, Zaki AF. Fatigue and brain atrophy in Egyptian patients with relapsing remitting multiple sclerosis. Multiple Sclerosis and Related Disorders. 2022;63:6. doi: 10.1016/j.msard.2022.103841. PubMed PMID: WOS:000832865100007.
27. Kopchak OO, Odintsova TA. Cognitive impairment and depression in patients with relapsing-remitting multiple sclerosis depending on age and neuroimaging findings. Egypt J Neurol Psychiatr Neurosurg. 2021;57(1):119. Epub 20210908. doi: 10.1186/s41983-021-00376-3. PubMed PMID: 34511865; PubMed Central PMCID: PMCPMC8424158.
28. Lazzarotto A, Margoni M, Franciotta S, Zywicki S, Riccardi A, Poggiali D, et al. Selective Cerebellar Atrophy Associates with Depression and Fatigue in the Early Phases of Relapse-Onset Multiple Sclerosis. Cerebellum. 2020;19(2):192-200. doi: 10.1007/s12311-019-01096-4. PubMed PMID: 31898280.
29. Morgante F, Dattola V, Crupi D, Russo M, Rizzo V, Ghilardi MF, et al. Is central fatigue in multiple sclerosis a disorder of movement preparation? J Neurol. 2011;258(2):263-72. Epub 20100922. doi: 10.1007/s00415-010-5742-x. PubMed PMID: 20859746.
30. Niepel G, Tench Ch R, Morgan PS, Evangelou N, Auer DP, Constantinescu CS. Deep gray matter and fatigue in MS: a T1 relaxation time study. J Neurol. 2006;253(7):896-902. Epub 20060313. doi: 10.1007/s00415-006-0128-9. PubMed PMID: 16525881.
31. Nigro S, Passamonti L, Riccelli R, Toschi N, Rocca F, Valentino P, et al. Structural 'connectomic' alterations in the limbic system of multiple sclerosis patients with major depression. Mult Scler. 2015;21(8):1003-12. Epub 20141222. doi: 10.1177/1352458514558474. PubMed PMID: 25533294.
32. Nygaard GO, Walhovd KB, Sowa P, Chepkoech JL, Bjørnerud A, Due-Tønnessen P, et al. Cortical thickness and surface area relate to specific symptoms in early relapsing-remitting multiple sclerosis. Mult Scler. 2015;21(4):402-14. Epub 20140819. doi: 10.1177/1352458514543811. PubMed PMID: 25139946.
33. Pardini M, Bonzano L, Mancardi GL, Roccatagliata L. Frontal networks play a role in fatigue perception in multiple sclerosis. Behav Neurosci. 2010;124(3):329-36. doi: 10.1037/a0019585. PubMed PMID: 20528076.
34. Pardini M, Bonzano L, Bergamino M, Bommarito G, Feraco P, Murugavel A, et al. Cingulum bundle alterations underlie subjective fatigue in multiple sclerosis. Mult Scler. 2015;21(4):442-7. Epub 20140821. doi: 10.1177/1352458514546791. PubMed PMID: 25145692.
35. Pokryszko-Dragan A, Banaszek A, Nowakowska-Kotas M, Jeżowska-Jurczyk K, Dziadkowiak E, Gruszka E, et al. Diffusion tensor imaging findings in the multiple sclerosis patients and their relationships to various aspects of disability. J Neurol Sci. 2018;391:127-33. Epub 20180613. doi: 10.1016/j.jns.2018.06.007. PubMed PMID: 30103962.
36. Pravatà E, Zecca C, Sestieri C, Caulo M, Riccitelli GC, Rocca MA, et al. Hyperconnectivity of the dorsolateral prefrontal cortex following mental effort in multiple sclerosis patients with cognitive fatigue. Mult Scler. 2016;22(13):1665-75. Epub 20160204. doi: 10.1177/1352458515625806. PubMed PMID: 26846988.

37. Riccelli R, Passamonti L, Cerasa A, Nigro S, Cavalli SM, Chiriaco C, et al. Individual differences in depression are associated with abnormal function of the limbic system in multiple sclerosis patients. Mult Scler. 2016;22(8):1094-105. Epub 20151009. doi: 10.1177/1352458515606987. PubMed PMID: 26453680.
38. Rocca MA, Gatti R, Agosta F, Broglia P, Rossi P, Riboldi E, et al. Influence of task complexity during coordinated hand and foot movements in MS patients with and without fatigue. A kinematic and functional MRI study. J Neurol. 2009;256(3):470-82. Epub 20090306. doi: 10.1007/s00415-009-0116-y. PubMed PMID: 19271107.
39. Rocca MA, Meani A, Riccitelli GC, Colombo B, Rodegher M, Falini A, et al. Abnormal adaptation over time of motor network recruitment in multiple sclerosis patients with fatigue. Mult Scler. 2016;22(9):1144-53. Epub 20151022. doi: 10.1177/1352458515614407. PubMed PMID: 26493126.
40. Rojas JI, Sanchez F, Patrucco L, Miguez J, Besada C, Cristiano E. Brain structural changes in patients in the early stages of multiple sclerosis with depression. Neurol Res. 2017;39(7):596-600. Epub 20170301. doi: 10.1080/01616412.2017.1298279. PubMed PMID: 28245725.
41. Romanello A, Krohn S, von Schwanenflug N, Chien C, Bellmann-Strobl J, Ruprecht K, et al. Functional connectivity dynamics reflect disability and multi-domain clinical impairment in patients with relapsing-remitting multiple sclerosis. Neuroimage Clin. 2022;36:103203. Epub 20220916. doi: 10.1016/j.nicl.2022.103203. PubMed PMID: 36179389; PubMed Central PMCID: PMCPMC9668632.
42. Ruiz-Rizzo AL, Bublak P, Kluckow S, Finke K, Gaser C, Schwab M, et al. Neural distinctiveness of fatigue and low sleep quality in multiple sclerosis. European Journal of Neurology. 2022;29(10):3017-27. doi: 10.1111/ene.15445.
43. Saberi A, Abdolalizadeh A, Mohammadi E, Nahayati MA, Bagheri H, Shekarchi B, et al. Thalamic shape abnormalities in patients with multiple sclerosis-related fatigue. Neuroreport. 2021;32(6):438-42. doi: 10.1097/wnr.0000000000001616. PubMed PMID: 33788816.
44. Specogna I, Casagrande F, Lorusso A, Catalan M, Gorian A, Zugna L, et al. Functional MRI during the execution of a motor task in patients with multiple sclerosis and fatigue. Radiol Med. 2012;117(8):1398-407. Epub 20120622. doi: 10.1007/s11547-012-0845-3. PubMed PMID: 22729506.
45. Štecková T, Hluštík P, Sládková V, Odstrčil F, Mareš J, Kaňovský P. Thalamic atrophy and cognitive impairment in clinically isolated syndrome and multiple sclerosis. J Neurol Sci. 2014;342(1-2):62-8. Epub 20140430. doi: 10.1016/j.jns.2014.04.026. PubMed PMID: 24819917.
46. Svolgaard O, Andersen KW, Bauer C, Madsen KH, Blinkenberg M, Selleberg F, et al. Cerebellar and premotor activity during a non-fatiguing grip task reflects motor fatigue in relapsing-remitting multiple sclerosis. PLoS One. 2018;13(10):e0201162. Epub 20181024. doi: 10.1371/journal.pone.0201162. PubMed PMID: 30356315; PubMed Central PMCID: PMCPMC6200185.
47. Svolgaard O, Andersen KW, Bauer C, Madsen KH, Blinkenberg M, Sellebjerg F, et al. Mapping grip-force related brain activity after a fatiguing motor task in multiple sclerosis. Neuroimage-Clinical. 2022;36:11. doi: 10.1016/j.nicl.2022.103147. PubMed PMID: WOS:000889292900001.
48. Téllez N, Alonso J, Río J, Tintoré M, Nos C, Montalban X, et al. The basal ganglia: a substrate for fatigue in multiple sclerosis. Neuroradiology. 2008;50(1):17-23. Epub 20071023. doi: 10.1007/s00234-007-0304-3. PubMed PMID: 17955232.
49. Tijhuis FB, Broeders TAA, Santos FAN, Schoonheim MM, Killestein J, Leurs CE, et al. Dynamic functional connectivity as a neural correlate of fatigue in multiple sclerosis. Neuroimage-Clinical. 2021;29:9. doi: 10.1016/j.nicl.2020.102556. PubMed PMID: WOS:000620121700041.
50. Tomasevic L, Zito G, Pasqualetti P, Filippi M, Landi D, Ghazaryan A, et al. Cortico-muscular coherence as an index of fatigue in multiple sclerosis. Mult Scler. 2013;19(3):334-43. Epub 20120703. doi: 10.1177/1352458512452921. PubMed PMID: 22760098.

51. Wilting J, Rolfsnes HO, Zimmermann H, Behrens M, Fleischer V, Zipp F, et al. Structural correlates for fatigue in early relapsing remitting multiple sclerosis. Eur Radiol. 2016;26(2):515-23. Epub 20150531. doi: 10.1007/s00330-015-3857-2. PubMed PMID: 26026721.
52. Wu L, Zhang Y, Zhou FQ, Gao L, He LC, Zeng XJ, et al. Altered intra- and interregional synchronization in relapsing-remitting multiple sclerosis: a resting-state fMRI study. Neuropsychiatric Disease and Treatment. 2016;12:853-62. doi: 10.2147/ndt.S98962. PubMed PMID: WOS:000374146300001.
53. Wu L, Huang M, Zhou F, Zeng X, Gong H. Distributed causality in resting-state network connectivity in the acute and remitting phases of RRMS. BMC Neuroscience. 2020;21(1). doi: 10.1186/s12868-020-00590-4.
54. Yaldizli Ö, Glassl S, Sturm D, Papadopoulou A, Gass A, Tettenborn B, et al. Fatigue and progression of corpus callosum atrophy in multiple sclerosis. J Neurol. 2011;258(12):2199-205. Epub 20110519. doi: 10.1007/s00415-011-6091-0. PubMed PMID: 21594686.
55. Yaldizli Ö, Penner IK, Yonekawa T, Naegelin Y, Kuhle J, Pardini M, et al. The association between olfactory bulb volume, cognitive dysfunction, physical disability and depression in multiple sclerosis. Eur J Neurol. 2016;23(3):510-9. Epub 20151119. doi: 10.1111/ene.12891. PubMed PMID: 26699999.
56. Yarraguntla K, Seraji-Bozorgzad N, Lichtman-Mikol S, Razmjou S, Bao F, Sriwastava S, et al. Multiple Sclerosis Fatigue: A Longitudinal Structural MRI and Diffusion Tensor Imaging Study. J Neuroimaging. 2018;28(6):650-5. Epub 20180723. doi: 10.1111/jon.12548. PubMed PMID: 30039613.
57. Yarraguntla K, Bao F, Lichtman-Mikol S, Razmjou S, Santiago-Martinez C, Seraji-Bozorgzad N, et al. Characterizing Fatigue-Related White Matter Changes in MS: A Proton Magnetic Resonance Spectroscopy Study. Brain Sci. 2019;9(5). Epub 20190527. doi: 10.3390/brainsci9050122. PubMed PMID: 31137831; PubMed Central PMCID: PMCPMC6562940.
58. Zellini F, Niepel G, Tench CR, Constantinescu CS. Hypothalamic involvement assessed by T1 relaxation time in patients with relapsing-remitting multiple sclerosis. Mult Scler. 2009;15(12):1442-9. Epub 20091207. doi: 10.1177/1352458509350306. PubMed PMID: 19995847.
59. Zhou F, Zhuang Y, Gong H, Wang B, Wang X, Chen Q, et al. Altered inter-subregion connectivity of the default mode network in relapsing remitting multiple sclerosis: A functional and structural connectivity study. PLoS ONE. 2014;9(7). doi: 10.1371/journal.pone.0101198.
60. Zhou F, Gong H, Chen Q, Wang B, Peng Y, Zhuang Y, et al. Intrinsic Functional Plasticity of the Thalamocortical System in Minimally Disabled Patients with Relapsing-Remitting Multiple Sclerosis. Front Hum Neurosci. 2016;10:2. Epub 20160125. doi: 10.3389/fnhum.2016.00002. PubMed PMID: 26834600; PubMed Central PMCID: PMCPMC4725198.
