# Supplementary material for: Brain connectivity changes underlying depression and fatigue in relapsing-remitting multiple sclerosis: A systematic review
Source: PLoS One. 2024 Mar 29;19(3):e0299634. doi: 10.1371/journal.pone.0299634 (PMC10980255; doi:10.1371/journal.pone.0299634)
Supplement: S8 Table — (PDF) [file pone.0299634.s011.pdf]

S8 Table. Negative findings explicitly reported in the included studies of this systematic review. As studies did not fully report on all observed null findings, this table serves as an approximate summary of negative findings.

MRI	Area	Depression	Overlap	Fatigue
Conventional MRI	Amygdala	Riccelli et al. [1]	Riccelli et al. [1]; Kever et al. [2]	Kever et al. [2]
	Hippocampus	Riccelli et al. [1]	-	-
	Basal ganglia volumes	-	-	Finke et al. [3]; Codella et al. [4]
	CA1, Subiculum, Entorhinal Cortex	Gold et al. [5]	-	-
	Caudate nucleus, superior ventral striatum	-	-	Jaeger et al. [6]
	Cerebral cortex of the frontal lobe	-	-	Codella et al. [4]
	Cerebral volume	-	-	Pardini et al. [7]
	corpus callosum index	Benesova et al. [8]	Yaldizli et al. [9]; Benesova et al. [8]	Yaldizli et al. [9]
	Cortical area	Nygaard et al. [10]	-	-
	Global atrophy	Gold et al. [5]	-	-
	Global brain parenchymal fraction	-	-	Cavallari et al. [11]; Andreasen et al. [12]
	Global lesion volume	Gold et al. [5]; Riccelli et al. [1]; Beaudoin et al. [13]; Romanello et al. [14]	Zellini et al. [15]; Niepel et al. [16]; Yaldizli et al. [9]; Calabrese et al. [17]; Gold et al. [5]; Filippi et al. [18]; Rocca et al. [19]; Rocca et al. [20]; Specogna et al. [21]; Svolgaard et al. [22]; Pravatà et al. [23]; Pardini et al. [7]; Wilting et al. [24]; Andreasen et al. [12]; Bisecco et al. [25]; Riccelli et al. [1]; Beaudoin et al. [13]; Alshehri et al. [26]; Romanello et al. [14]	ellini et al. [15]; Niepel et al. [16]; Yaldizli et al. [9]; Calabrese et al. [17]; Yaldizli et al. [9]; Filippi et al. [18]; Rocca et al. [19]; Rocca et al. [20]; Specogna et al. [21]; Svolgaard et al. [22]; Pravatà et al. [23]; Pardini et al. [7]; Wilting et al. [24]; Andreasen et al. [12]; Bisecco et al. [25]; Ruiz-Rizzo et al .[27]; Beaudoin et al. [13]; Alshehri et al. [26]; Romanello et al. [14]
	GM density, WM integrity	-	-	Finke et al. [3]

	Hypothalamic volume	-	-	Zellini et al. [15]
	Lesion occurrence	-	-	Bisecco et al. [25]
	NAWM	-	-	Andreasen et al. [12]
	Normalized brain volume	Riccelli et al. [1];	Bisecco et al. [25]; Riccelli et al. [1]; Rocca et al. [19]; Filippi et al. [18]; Rocca et al. [20]	Bisecco et al. [25]; Rocca et al. [19]; Filippi et al. [18]; Rocca et al. [20]
	Olfactory bulb volume	Yaldizli et al. [28]	-	-
	Putamen and caudate	-	-	Niepel et al. [16]
	Striatum	-	-	Jaeger et al. [6]
	Subgenual cingulate cortex	Riccelli et al. [1]	-	-
	T1 lesion burden	Rojas et al. [29]	-	-
	Temporal lobe	Benesova et al. [8]	-	-
	Thalamus volume	-	-	Saberi et al. [30]
	Total GM volume	Nigro et al. [31]	Nigro et al. [31]; Bisecco et al. [25]; Riccelli et al. [1]; Rocca et al. [19]	Bisecco et al. [25]; Rocca et al. [19]; Tijhuis et al. [32]
	Total intracranial volume	-	-	Saberi et al. [30]
	Volume fraction	-	Hildebrandt et al. [33]	-
	WM fraction	-	-	Wilting et al. [24]
	WM volume	Roj; as et al. [29]; Riccelli et al. [1]	Rojas et al. [29]; Bisecco et al. [25]; Riccelli et al. [1]; Rocca et al. [19]	Bisecco et al. [25]; Tijhuis et al. [32]; Rocca et al. [19]
**Structural connectivity**	FA, MD, RD, and AD	-	-	Bisecco et al. [25]
	DTI metrics in total brain WM	-	-	Alshehri et al. [26]
	DTI metrics in WML	-	-	Alshehri et al. [26]
	Frontal cortex	-	-	Wilting et al. [24]
	Global FA	Rojas et al. [29]	-	-
	Global FA, MD, PD, RD	-	-	Finke et al. [3]
	Global MD	-	-	Yarraguntla et al. [34]
	Thalamus	-	-	Bisecco et al. [25]

Functional connectivity	Caudate and putamen and the dlPFC	-	-	Jaeger et al. [6]
	Cortical clusters in the prefrontal, premotor, sensorimotor, parietal, occipital cortex, cerebellum, basal ganglia bilaterally, putamen as ROI	-	-	Svolgaard et al. [22]
	Hippocampus and the rest of the brain	-	-	Golde et al. [35]
AD: axial diffusivity; dlPFC: dorsolateral prefrontal cortex; DTI: diffusion tensor imaging; FA: fractional anisotropy; GM: gray matter; MD: mean diffusivity; MRI: magnetic resonance imaging; NAWM: normal appearing WM; RD: radial diffusivity; ROI: region of interest; WM: white matter; WML: white matter lesions.				

References:

1. Riccelli R, Passamonti L, Cerasa A, Nigro S, Cavalli SM, Chiriaco C, et al. Individual differences in depression are associated with abnormal function of the limbic system in multiple sclerosis patients. Mult Scler. 2016;22(8):1094-105. Epub 20151009. doi: 10.1177/1352458515606987. PubMed PMID: 26453680.
2. Kever A, Buyukturkoglu K, Levin SN, Riley CS, De Jager P, Leavitt VM. Associations of social network structure with cognition and amygdala volume in multiple sclerosis: An exploratory investigation. Multiple Sclerosis Journal. 2022;28(2):228-36. doi: 10.1177/13524585211018349. PubMed PMID: WOS:000656009600001.
3. Finke C, Schlichting J, Papazoglou S, Scheel M, Freing A, Soemmer C, et al. Altered basal ganglia functional connectivity in multiple sclerosis patients with fatigue. Mult Scler. 2015;21(7):925-34. Epub 20141112. doi: 10.1177/1352458514555784. PubMed PMID: 25392321.
4. Codella M, Rocca MA, Colombo B, Martinelli-Boneschi F, Comi G, Filippi M. Cerebral grey matter pathology and fatigue in patients with multiple sclerosis: a preliminary study. J Neurol Sci. 2002;194(1):71-4. doi: 10.1016/s0022-510x(01)00682-7. PubMed PMID: 11809169.
5. Gold SM, Kern KC, O'Connor MF, Montag MJ, Kim A, Yoo YS, et al. Smaller cornu ammonis 2-3/dentate gyrus volumes and elevated cortisol in multiple sclerosis patients with depressive symptoms. Biol Psychiatry. 2010;68(6):553-9. Epub 20100619. doi: 10.1016/j.biopsych.2010.04.025. PubMed PMID: 20646680; PubMed Central PMCID: PMCPMC3122328.
6. Jaeger S, Paul F, Scheel M, Brandt A, Heine J, Pach D, et al. Multiple sclerosis-related fatigue: Altered resting-state functional connectivity of the ventral striatum and dorsolateral prefrontal cortex. Mult Scler. 2019;25(4):554-64. Epub 20180221. doi: 10.1177/1352458518758911. PubMed PMID: 29464981.

7. Pardini M, Bonzano L, Mancardi GL, Roccatagliata L. Frontal networks play a role in fatigue perception in multiple sclerosis. Behav Neurosci. 2010;124(3):329-36. doi: 10.1037/a0019585. PubMed PMID: 20528076.
8. Benesova Y, Niedermayerova I, Mechl M, Havlikova P. The relation between brain MRI lesions and depressive symptoms in multiple sclerosis. Bratisl Lek Listy. 2003;104(4-5):174-6. PubMed PMID: 14604264.
9. Yaldizli Ö, Glassl S, Sturm D, Papadopoulou A, Gass A, Tettenborn B, et al. Fatigue and progression of corpus callosum atrophy in multiple sclerosis. J Neurol. 2011;258(12):2199-205. Epub 20110519. doi: 10.1007/s00415-011-6091-0. PubMed PMID: 21594686.
10. Nygaard GO, Walhovd KB, Sowa P, Chepkoech JL, Bjørnerud A, Due-Tønnessen P, et al. Cortical thickness and surface area relate to specific symptoms in early relapsing-remitting multiple sclerosis. Mult Scler. 2015;21(4):402-14. Epub 20140819. doi: 10.1177/1352458514543811. PubMed PMID: 25139946.
11. Cavallari M, Palotai M, Glanz BI, Egorova S, Prieto JC, Healy BC, et al. Fatigue predicts disease worsening in relapsing-remitting multiple sclerosis patients. Multiple Sclerosis Journal. 2016;22(14):1841-9. doi: 10.1177/1352458516635874. PubMed PMID: WOS:000390576600013.
12. Andreasen AK, Jakobsen J, Soerensen L, Andersen H, Petersen T, Bjarkam CR, et al. Regional brain atrophy in primary fatigued patients with multiple sclerosis. Neuroimage. 2010;50(2):608-15. Epub 20100106. doi: 10.1016/j.neuroimage.2009.12.118. PubMed PMID: 20060048.
13. Beaudoin AM, Rheault F, Theaud G, Laberge F, Whittingstall K, Lamontagne A, et al. Modern Technology in Multi-Shell Diffusion MRI Reveals Diffuse White Matter Changes in Young Adults With Relapsing-Remitting Multiple Sclerosis. Frontiers in Neuroscience. 2021;15:13. doi: 10.3389/fnins.2021.665017. PubMed PMID: WOS:000687832800001.
14. Romanello A, Krohn S, von Schwanenflug N, Chien C, Bellmann-Strobl J, Ruprecht K, et al. Functional connectivity dynamics reflect disability and multi-domain clinical impairment in patients with relapsing-remitting multiple sclerosis. Neuroimage Clin. 2022;36:103203. Epub 20220916. doi: 10.1016/j.nicl.2022.103203. PubMed PMID: 36179389; PubMed Central PMCID: PMCPMC9668632.
15. Zellini F, Niepel G, Tench CR, Constantinescu CS. Hypothalamic involvement assessed by T1 relaxation time in patients with relapsing-remitting multiple sclerosis. Mult Scler. 2009;15(12):1442-9. Epub 20091207. doi: 10.1177/1352458509350306. PubMed PMID: 19995847.
16. Niepel G, Tench Ch R, Morgan PS, Evangelou N, Auer DP, Constantinescu CS. Deep gray matter and fatigue in MS: a T1 relaxation time study. J Neurol. 2006;253(7):896-902. Epub 20060313. doi: 10.1007/s00415-006-0128-9. PubMed PMID: 16525881.
17. Calabrese M, Rinaldi F, Grossi P, Mattisi I, Bernardi V, Favaretto A, et al. Basal ganglia and frontal/parietal cortical atrophy is associated with fatigue in relapsing-remitting multiple sclerosis. Mult Scler. 2010;16(10):1220-8. Epub 20100729. doi: 10.1177/1352458510376405. PubMed PMID: 20670981.
18. Filippi M, Rocca MA, Colombo B, Falini A, Codella M, Scotti G, et al. Functional magnetic resonance imaging correlates of fatigue in multiple sclerosis. Neuroimage. 2002;15(3):559-67. doi: 10.1006/nimg.2001.1011. PubMed PMID: 11848698.
19. Rocca MA, Meani A, Riccitelli GC, Colombo B, Rodegher M, Falini A, et al. Abnormal adaptation over time of motor network recruitment in multiple sclerosis patients with fatigue. Mult Scler. 2016;22(9):1144-53. Epub 20151022. doi: 10.1177/1352458515614407. PubMed PMID: 26493126.
20. Rocca MA, Gatti R, Agosta F, Broglia P, Rossi P, Riboldi E, et al. Influence of task complexity during coordinated hand and foot movements in MS patients with and without fatigue. A kinematic and functional MRI study. J Neurol. 2009;256(3):470-82. Epub 20090306. doi: 10.1007/s00415-009-0116-y. PubMed PMID: 19271107.

21. Specogna I, Casagrande F, Lorusso A, Catalan M, Gorian A, Zugna L, et al. Functional MRI during the execution of a motor task in patients with multiple sclerosis and fatigue. Radiol Med. 2012;117(8):1398-407. Epub 20120622. doi: 10.1007/s11547-012-0845-3. PubMed PMID: 22729506.
22. Svolgaard O, Andersen KW, Bauer C, Madsen KH, Blinkenberg M, Selleberg F, et al. Cerebellar and premotor activity during a non-fatiguing grip task reflects motor fatigue in relapsing-remitting multiple sclerosis. PLoS One. 2018;13(10):e0201162. Epub 20181024. doi: 10.1371/journal.pone.0201162. PubMed PMID: 30356315; PubMed Central PMCID: PMCPMC6200185.
23. Pravatà E, Zecca C, Sestieri C, Caulo M, Riccitelli GC, Rocca MA, et al. Hyperconnectivity of the dorsolateral prefrontal cortex following mental effort in multiple sclerosis patients with cognitive fatigue. Mult Scler. 2016;22(13):1665-75. Epub 20160204. doi: 10.1177/1352458515625806. PubMed PMID: 26846988.
24. Wilting J, Rolfsnes HO, Zimmermann H, Behrens M, Fleischer V, Zipp F, et al. Structural correlates for fatigue in early relapsing remitting multiple sclerosis. Eur Radiol. 2016;26(2):515-23. Epub 20150531. doi: 10.1007/s00330-015-3857-2. PubMed PMID: 26026721.
25. Bisecco A, Caiazzo G, d'Ambrosio A, Sacco R, Bonavita S, Docimo R, et al. Fatigue in multiple sclerosis: The contribution of occult white matter damage. Mult Scler. 2016;22(13):1676-84. Epub 20160204. doi: 10.1177/1352458516628331. PubMed PMID: 26846989.
26. Alshehri A, Al-iedani O, Arm J, Gholizadeh N, Billiet T, Lea R, et al. Neural diffusion tensor imaging metrics correlate with clinical measures in people with relapsing-remitting MS. Neuroradiology Journal. 2022;35(5):592-9. doi: 10.1177/19714009211067400.
27. Ruiz-Rizzo AL, Bublak P, Kluckow S, Finke K, Gaser C, Schwab M, et al. Neural distinctiveness of fatigue and low sleep quality in multiple sclerosis. European Journal of Neurology. 2022;29(10):3017-27. doi: 10.1111/ene.15445.
28. Yaldizli Ö, Penner IK, Yonekawa T, Naegelin Y, Kuhle J, Pardini M, et al. The association between olfactory bulb volume, cognitive dysfunction, physical disability and depression in multiple sclerosis. Eur J Neurol. 2016;23(3):510-9. Epub 20151119. doi: 10.1111/ene.12891. PubMed PMID: 26699999.
29. Rojas JI, Sanchez F, Patrucco L, Miguez J, Besada C, Cristiano E. Brain structural changes in patients in the early stages of multiple sclerosis with depression. Neurol Res. 2017;39(7):596-600. Epub 20170301. doi: 10.1080/01616412.2017.1298279. PubMed PMID: 28245725.
30. Saberi A, Abdolalizadeh A, Mohammadi E, Nahayati MA, Bagheri H, Shekarchi B, et al. Thalamic shape abnormalities in patients with multiple sclerosis-related fatigue. Neuroreport. 2021;32(6):438-42. doi: 10.1097/wnr.0000000000001616. PubMed PMID: 33788816.
31. Nigro S, Passamonti L, Riccelli R, Toschi N, Rocca F, Valentino P, et al. Structural 'connectomic' alterations in the limbic system of multiple sclerosis patients with major depression. Mult Scler. 2015;21(8):1003-12. Epub 20141222. doi: 10.1177/1352458514558474. PubMed PMID: 25533294.
32. Tijhuis FB, Broeders TAA, Santos FAN, Schoonheim MM, Killestein J, Leurs CE, et al. Dynamic functional connectivity as a neural correlate of fatigue in multiple sclerosis. Neuroimage-Clinical. 2021;29:9. doi: 10.1016/j.nicl.2020.102556. PubMed PMID: WOS:000620121700041.
33. Hildebrandt H, Hahn HK, Kraus JA, Schulte-Herbrüggen A, Schwarze B, Schwendemann G. Memory performance in multiple sclerosis patients correlates with central brain atrophy. Mult Scler. 2006;12(4):428-36. doi: 10.1191/1352458506ms1286oa. PubMed PMID: 16900756.
34. Yarraguntla K, Seraji-Bozorgzad N, Lichtman-Mikol S, Razmjou S, Bao F, Sriwastava S, et al. Multiple Sclerosis Fatigue: A Longitudinal Structural MRI and Diffusion Tensor Imaging Study. J Neuroimaging. 2018;28(6):650-5. Epub 20180723. doi: 10.1111/jon.12548. PubMed PMID: 30039613.
35. Golde S, Heine J, Pöttgen J, Mantwill M, Lau S, Wingenfeld K, et al. Distinct Functional Connectivity Signatures of Impaired Social Cognition in Multiple Sclerosis. Frontiers in Neurology. 2020;11. doi: 10.3389/fneur.2020.00507.
