# Supplementary material for: Brain connectivity changes underlying depression and fatigue in relapsing-remitting multiple sclerosis: A systematic review
Source: PLoS One. 2024 Mar 29;19(3):e0299634. doi: 10.1371/journal.pone.0299634 (PMC10980255; doi:10.1371/journal.pone.0299634)
Supplement: S9 Table — (PDF) [file pone.0299634.s012.pdf]

R. Zivadinov; J. Sepcic; D. Nasuelli; R. De Masi; L. Monti Bragadin; M. A. Tommasi; S. Zambito-Marsala; R. Moretti; A. Bratina; M. Ukmar; R. S. Pozzi-Mucelli; A. Grop; G. Cazzato; M. Zorzon	2001	A longitudinal study of brain atrophy and cognitive disturbances in the early phase of relapsing-remitting multiple sclerosis	10.1136/jnnp.70.6.773
M. L. Polliack; Y. Barak; A. Achiron	2001	Late-onset multiple sclerosis	10.1046/j.1532-5415.2001.49038.x
R. Zivadinov; L. M. Bragadin; D. Nasuelli; R. De Masi; M. Ukmar; R. S. Pozzi-Mucelli; R. Antonello; G. Cazzato; M. Zorzon	2001	Magnetic resonance imaging techniques as predictors of cognitive impairment in multiple sclerosis	
K. Morgen; R. Martin; R. D. Stone; J. Grafman; N. Kadom; H. F. McFarland; A. Marques	2001	FLAIR and magnetization transfer imaging of patients with post-treatment Lyme disease syndrome	10.1212/wnl.57.11.1980
D. I. Günal; M. Güleryüz; N. Avşar; S. Aktan	2001	Multiple sclerosis fatigue and relationship to depression and physical disability	
G. Giovannoni; A. J. Thompson; D. H. Miller; E. J. Thompson	2001	Fatigue is not associated with raised inflammatory markers in multiple sclerosis	10.1212/wnl.57.4.676
R. Bakshi; S. Ariyaratana; R. H. B. Benedict; L. Jacobs	2001	Fluid-attenuated inversion recovery magnetic resonance imaging detects cortical and juxtacortical multiple sclerosis lesions	10.1001/archneur.58.5.742
M. Filippi; M. A. Rocca; B. Colombo; A. Falini; M. Codella; G. Scotti; G. Comi	2002	Functional magnetic resonance imaging correlates of fatigue in multiple sclerosis	10.1006/nimg.2001.1011
M. Codella; M. A. Rocca; B. Colombo; F. Martinelli-Boneschi; G. Comi; M. Filippi	2002	Cerebral grey matter pathology and fatigue in patients with multiple sclerosis: A preliminary study	10.1016/S0022-510X(01)00682-7
V. Janardhan; R. Bakshi	2002	Quality of life in patients with multiple sclerosis: The impact of fatigue and depression	10.1016/s0022-510x(02)00312-x
L. Durelli; E. Verdun; P. Barbero; M. Bergui; E. Versino; A. Ghezzi; E. Montanari; M. Zaffaroni	2002	Every-other-day interferon beta-1b versus once-weekly interferon beta-1a for multiple sclerosis: results of a 2-year prospective randomised multicentre study (INCOMIN)	10.1016/s0140-6736(02)08430-1
M. Zorzon; R. Zivadinov; L. Locatelli; B. Stival; D. Nasuelli; A. Bratina; A. Bosco; M. A. Tommasi; R. S. Pozzi Mucelli; M. Ukmar; G. Cazzato	2003	Correlation of sexual dysfunction and brain magnetic resonance imaging in multiple sclerosis	10.1191/1352458503ms881sr
R. Zivadinov; M. Zorzon; L. Locatelli; B. Stival; F. Monti; D. Nasuelli; M. A. Tommasi; A. Bratina; G. Cazzato	2003	Sexual dysfunction in multiple sclerosis: a MRI, neurophysiological and urodynamic study	10.1016/s0022-510x(03)00025-x
Y. Benesova; I. Niedermayerova; M. Mechl; P. Havlikova	2003	The relation between brain MRI lesions and depressive symptoms in multiple sclerosis	

L. Locatelli; R. Zivadinov; A. Grop; M. Zorzon	2004	Frontal parenchymal atrophy measures in multiple sclerosis	10.1191/1352458504ms1093oa
C. J. Archibald; X. Wei; J. N. Scott; C. J. Wallace; Y. Zhang; L. M. Metz; J. R. Mitchell	2004	Posterior fossa lesion volume and slowed information processing in multiple sclerosis	10.1093/brain/awh167
A. T. White; J. H. Petajan	2004	Physiological measures of therapeutic response to interferon beta-1a treatment in remitting-relapsing MS	10.1016/j.clinph.2004.05.002
A. Perretti; P. Balbi; G. Orefice; L. Trojano; L. Marcantonio; V. Brescia-Morra; S. Ascione; F. Manganelli; G. Conte; L. Santoro	2004	Post-exercise facilitation and depression of motor evoked potentials to transcranial magnetic stimulation: a study in multiple sclerosis	10.1016/j.clinph.2004.03.028
C. Mainero; M. Inghilleri; P. Pantano; A. Conte; D. Lenzi; V. Frasca; L. Bozzao; C. Pozzilli	2004	Enhanced brain motor activity in patients with MS after a single dose of 3,4-diaminopyridine	10.1212/01.wnl.0000129263.14219.a8
B. Bielekova; N. Richert; T. Howard; G. Blevins; S. Markovic-Plese; J. McCartin; J. Würfel; J. Ohayon; T. A. Waldmann; H. F. McFarland; R. Martin	2004	Humanized anti-CD25 (daclizumab) inhibits disease activity in multiple sclerosis patients failing to respond to interferon β	10.1073/pnas.0402653101
D. R. Smith; B. Weinstock-Guttman; J. A. Cohen; X. Wei; C. Gutmann; R. Bakshi; M. Olek; L. Stone; S. Greenberg; D. Stuart; J. Orav; W. Stuart; H. Weiner	2005	A randomized blinded trial of combination therapy with cyclophosphamide in patients with active multiple sclerosis on interferon beta	10.1191/1352458505ms1210oa
J. Río; X. Montalban	2005	Interferon-β1b in the treatment of multiple sclerosis	10.1517/14656566.6.16.2877
A. Ostberg; F. Pittas; B. Taylor	2005	Use of low-dose mitozantrone to treat aggressive multiple sclerosis: a single-centre open-label study using patient self-assessment and clinical measures of multiple sclerosis status	10.1111/j.1445-5994.2005.00862.x
J. Oger; G. Francis; P. Chang	2005	Prospective assessment of changing from placebo to IFN beta-1a in relapsing MS: The PRISMS study	10.1016/j.jns.2005.05.014
B. K. Kleinschmidt-DeMasters; K. L. Tyler	2005	Progressive multifocal leukoencephalopathy complicating treatment with natalizumab and interferon beta-1a for multiple sclerosis	10.1056/NEJMoa051782
D. R. Jeffery; N. Chepuri; D. Durden; J. Burdette	2005	A pilot trial of combination therapy with mitoxantrone and interferon beta-Ib using monthly gadolinium-enhanced magnetic resonance imaging	10.1191/1352458505ms1154oa
D. A. Woo; M. J. Olek; E. M. Frohman	2006	Diagnosis and management of multiple sclerosis: case studies	10.1016/j.ncl.2006.01.002

G. Niepel; R. Tench Ch; P. S. Morgan; N. Evangelou; D. P. Auer; C. S. Constantinescu	2006	Deep gray matter and fatigue in MS: a T1 relaxation time study	10.1007/s00415-006-0128-9
H. Hildebrandt; H. K. Hahn; J. A. Kraus; A. Schulte-Herbrüggen; B. Schwarze; G. Schwendemann	2006	Memory performance in multiple sclerosis patients correlates with central brain atrophy	10.1191/1352458506ms1286oa
S. Cader; A. Cifelli; Y. Abu-Omar; J. Palace; P. M. Matthews	2006	Reduced brain functional reserve and altered functional connectivity in patients with multiple sclerosis	10.1093/brain/awh670
M. C. Bonnet; M. S. A. Deloire; E. Salort; V. Dousset; K. G. Petry; B. Brochet	2006	Evidence of cognitive compensation associated with educational level in early relapsing-remitting multiple sclerosis	10.1016/j.jns.2006.08.002
C. H. Polman; P. W. O'Connor; E. Havrdova; M. Hutchinson; L. Kappos; D. H. Miller; J. T. Phillips; F. D. Lublin; G. Giovannoni; A. Wajgt; M. Toal; F. Lynn; M. A. Panzara; A. W. Sandrock	2006	A randomized, placebo-controlled trial of natalizumab for relapsing multiple sclerosis	10.1056/NEJMoa044397
M. Filippi; J. S. Wolinsky; G. Comi	2006	Effects of oral glatiramer acetate on clinical and MRI-monitored disease activity in patients with relapsing multiple sclerosis: A multicentre, double-blind, randomised, placebo-controlled study	10.1016/S1474-4422(06)70327-1
C. Caon; M. Din; W. Ching; A. Tselis; R. Lisak; O. Khan	2006	Clinical course after change of immunomodulating therapy in relapsing - Remitting multiple sclerosis	10.1111/j.1468-1331.2006.01273.x
C. Bensa; C. Bertogliati; S. Chanalet; G. Malandain; P. Bedoucha; C. Lebrun	2006	Early detection of cognitive impairment in relapsing-remitting multiple sclerosis: Functional-anatomical correlations and longitudinal follow-up	10.1016/s0035-3787(06)75135-9
B. A. Parmenter; R. Zivadinov; L. Kerenyi; R. Gavett; B. Weinstock-Guttman; M. G. Dwyer; N. Garg; F. Munschauer; R. H. B. Benedict	2007	Validity of the Wisconsin card sorting and Delis-Kaplan Executive Function System (DKEFS) sorting tests in multiple sclerosis	10.1080/13803390600672163
B. A. Parmenter; J. L. Shucard; D. W. Shucard	2007	Information processing deficits in multiple sclerosis: A matter of complexity	10.1017/s1355617707070580
M. Inglese; S. J. Park; G. Johnson; J. S. Babb; L. Miles; H. Jaggi; J. Herbert; R. I. Grossman	2007	Deep gray matter perfusion in multiple sclerosis - Dynamic susceptibility contrast perfusion magnetic resonance imaging at 3 T	10.1001/archneur.64.2.196

G. Tedeschi; D. Dinacci; L. Lavorgna; A. Prinster; G. Savettieri; A. Quattrone; P. Livrea; C. Messina; A. Reggio; G. Servillo; V. Bresciamorra; G. Orefice; M. Paciello; A. Brunetti; A. Paolillo; G. Coniglio; S. Bonavita; A. Di Costanzo; A. Bellacosa; P. Valentino; M. Quarantelli; F. Patti; G. Salemi; E. Cammarata; I. Simone; M. Salvatore; V. Bonavita; B. Alfano	2007	Correlation between fatigue and brain atrophy and lesion load in multiple sclerosis patients independent of disability	10.1016/j.jns.2007.07.004
M. A. Rocca; F. Agosta; B. Colombo; D. M. Mezzapesa; A. Falini; G. Comi; M. Filippi	2007	fMRI changes in relapsing-remitting multiple sclerosis patients complaining of fatigue after IFNβ-1a injection	10.1002/hbm.20279
T. Matsuoka; T. Matsushita; Y. Kawano; M. Osoegawa; H. Ochi; T. Ishizu; M. Minohara; H. Kikuchi; F. Mihara; Y. Ohyagi; J. I. Kira	2007	Heterogeneity of aquaporin-4 autoimmunity and spinal cord lesions in multiple sclerosis in Japanese	10.1093/brain/awm027
K. P. Johnson	2007	Control of multiple sclerosis relapses with immunomodulating agents	10.1016/j.jns.2007.01.060
H. Hildebrandt; M. Lanz; H. K. Hahn; E. Hoffmann; B. Schwarze; G. Schwendemann; J. A. Kraus	2007	Cognitive training in MS: Effects and relation to brain atrophy	
J. A. Cohen; M. Rovaris; A. D. Goodman; D. Ladkani; D. Wynn; M. Filippi	2007	Randomized, double-blind, dose-comparison study of glatiramer acetate in relapsing-remitting MS	10.1212/01.wnl.0000257109.61671.06
A. Bar-Or; T. Vollmer; J. Antel; D. L. Arnold; C. A. Bodner; D. Campagnolo; J. Gianettoni; F. Jalili; N. Kachuck; Y. Lapierre; M. Niino; J. Oger; M. Price; S. Rhodes; W. H. Robinson; F. D. Shi; P. J. Utz; F. Valone; L. Weiner; L. Steinman; H. Garren	2007	Induction of antigen-specific tolerance in multiple sclerosis after immunization with DNA encoding myelin basic protein in a randomized, placebo-controlled phase 1/2 trial	10.1001/archneur.64.10.nct70002
M. P. Amato; N. De Stefano	2007	Longitudinal follow-up of benign" multiple sclerosis at 20 years"	10.1212/01.wnl.0000281903.32474.15
N. Téllez; J. Alonso; J. Río; M. Tintoré; C. Nos; X. Montalban; A. Rovira	2008	The basal ganglia: A substrate for fatigue in multiple sclerosis	10.1007/s00234-007-0304-3
M. Summers; J. Swanton; K. Fernando; C. Dalton; D. H. Miller; L. Cipolotti; M. A. Ron	2008	Cognitive impairment in multiple sclerosis can be predicted by imaging early in the disease	10.1136/jnnp.2007.138685

C. Lebrun; C. Bensa; M. Debouverie; J. De Seze; S. Wiertlievski; B. Brochet; P. Clavelou; D. Brassat; P. Labauge; E. Roullet	2008	Unexpected multiple sclerosis: Follow-up of 30 patients with magnetic resonance imaging and clinical conversion profile	10.1136/jnnp.2006.108274
B. Kis; B. Rumberg; P. Berlit	2008	Clinical characteristics of patients with late-onset multiple sclerosis	10.1007/s00415-008-0778-x
M. C. Tartaglia; S. Narayanan; D. L. Arnold	2008	Mental fatigue alters the pattern and increases the volume of cerebral activation required for a motor task in multiple sclerosis patients with fatigue	10.1111/j.1468-1331.2008.02090.x
J. P. Mostert; F. Admiraal-Behloul; J. M. Hoogduin; J. Luyendijk; D. J. Heersema; M. A. van Buchem; J. De Keyser	2008	Effects of fluoxetine on disease activity in relapsing multiple sclerosis: a double-blind, placebo-controlled, exploratory study	10.1136/jnnp.2007.139345
A. Minagar; J. S. Alexander; R. N. Schwendimann; R. E. Kelley; E. Gonzalez-Toledo; J. J. Jimenez; L. Mauro; W. Jy; S. J. Smith	2008	Combination therapy with interferon beta-1a and doxycycline in multiple sclerosis: An open-label trial	10.1001/archneurol.2007.41
D. D. Mikol; F. Barkhof; P. Chang; P. K. Coyle; D. R. Jeffery; S. R. Schwid; B. Stubinski; B. M. Uitdehaag	2008	Comparison of subcutaneous interferon beta-1a with glatiramer acetate in patients with relapsing multiple sclerosis (the REbif vs Glatiramer Acetate in Relapsing MS Disease [REGARD] study): a multicentre, randomised, parallel, open-label trial	10.1016/S1474-4422(08)70200-X
M. Koch; M. Uyttenboogaart; A. van Harten; M. Heerings; J. De Keyser	2008	Fatigue, depression and progression in multiple sclerosis	10.1177/1352458508088937
S. Knudsen; P. J. Jennum; K. Korsholm; S. P. Sheikh; S. Gammeltoft; J. L. Frederiksen	2008	Normal levels of cerebrospinal fluid hypocretin-1 and daytime sleepiness during attacks of relapsing-remitting multiple sclerosis and monosymptomatic optic neuritis	10.1177/1352458508088939
S. L. Hauser; E. Waubant; D. L. Arnold; T. Vollmer; J. Antel; R. J. Fox; A. Bar-Or; M. Panzara; N. Sarkar; S. Agarwal; A. Langer-Gould; C. H. Smith	2008	B-cell depletion with rituximab in relapsing-remitting multiple sclerosis	10.1056/NEJMoa0706383
E. Portaccio; B. Goretti; V. Zipoli; B. Nacmias; M. L. Stromillo; M. L. Bartolozzi; G. Siracusa; L. Guidi; A. Federico; S. Sorbi; N. De Stefano; M. P. Amato	2009	APOE-epsilon4 is not associated with cognitive impairment in relapsing-remitting multiple sclerosis	10.1177/1352458509348512
F. Zellini; G. Niepel; C. R. Tench; C. S. Constantinescu	2009	Hypothalamic involvement assessed by T1 relaxation time in patients with relapsing-remitting multiple sclerosis	10.1177/1352458509350306

J. Sepulcre; J. C. Masdeu; J. Goñi; G. Arrondo; N. Vélez de Mendizábal; B. Bejarano; P. Villoslada	2009	Fatigue in multiple sclerosis is associated with the disruption of frontal and parietal pathways	10.1177/1352458508098373
M. A. Rocca; R. Gatti; F. Agosta; P. Broglia; P. Rossi; E. Riboldi; M. Corti; G. Comi; M. Filippi	2009	Influence of task complexity during coordinated hand and foot movements in MS patients with and without fatigue. A kinematic and functional MRI study	10.1007/s00415-009-0116-y
E. Portaccio; B. Goretti; V. Zipoli; B. Nacmias; M. L. Stromillo; M. L. Bartolozzi; G. Siracusa; L. Guidi; A. Federico; S. Sorbi; N. De Stefano; M. Pia Amato	2009	APOE-ε4 is not associated with cognitive impairment in relapsing - Remitting multiple sclerosis	10.1177/1352458509348512
F. Patti; M. P. Amato; M. Trojano; S. Bastianello; M. R. Tola; B. Goretti; L. Caniatti; E. Di Monte; P. Ferrazza; V. Brescia Morra; S. Lo Fermo; O. Picconi; G. Luccichenti; R. Vecchio; D. Maimone; S. Messina; C. Gasperini; V. Orefice; C. Florio; E. Portaccio; V. Zipoli; A. Bertolotto; P. Bramanti; E. Sessa; D. Centonze; S. Cottone; G. Salemi; M. Falcini; P. Gallo; P. Perini; G. L. Gigli; G. Giuliani; L. M. Grimaldi; L. Murri; A. Lugaresi; F. Monaco; E. Montanari; L. Motti; S. Neri; M. Paciello; L. Provinciali; M. Ragno; G. Rosati; S. Ruggieri; M. R. Tola; P. Tonali; A. P. Batocchi; M. F. De Caro; A. Ghezzi; M. Zaffaroni; P. Zolo; M. Zorzon; M. Signorino; E. Scarpini; L. Durelli; A. Carolei; M. Todaro; D. Spitaleri; A. Tartaglione	2009	Cognitive impairment and its relation with disease measures in mildly disabled patients with relapsing-remitting multiple sclerosis: Baseline results from the Cognitive Impairment in Multiple Sclerosis (COGIMUS) study	10.1177/1352458509105544
L. Passamonti; A. Cerasa; M. Liguori; M. C. Gioia; P. Valentino; R. Nisticò; A. Quattrone; F. Fera	2009	Neurobiological mechanisms underlying emotional processing in relapsing-remitting multiple sclerosis	10.1093/brain/awp095
R. A. Rudick; A. Pace; M. R. S. Rani; R. Hyde; M. Panzara; S. Appachi; J. Shrock; S. L. Maurer; P. A. Calabresi; C. Confavreux; S. L. Galetta; F. D. Lublin; E. W. Radue; R. M. Ransohoff	2009	Effect of statins on clinical and molecular responses to intramuscular interferon beta-1a	10.1212/WNL.0b013e3181a92b96
S. D. Roosendaal; B. Moraal; P. J. W. Pouwels; H. Vrenken; J. A. Castelijns; F. Barkhof; J. J. G. Geurts	2009	Accumulation of cortical lesions in MS: relation with cognitive impairment	10.1177/1352458509102907

N. Putzki; O. Yaldizli; B. Tettenborn; H. C. Diener	2009	Multiple sclerosis associated fatigue during natalizumab treatment	10.1016/j.jns.2009.06.004
N. Putzki; K. Kollia; S. Woods; E. Igwe; H. C. Diener; V. Limmroth	2009	Natalizumab is effective as second line therapy in the treatment of relapsing remitting multiple sclerosis	10.1111/j.1468-1331.2008.02519.x
L. M. Metz; D. Li; A. Traboulsee; M. L. Myles; P. Duquette; J. Godin; M. Constantin; V. W. Yong; M. M. C. Yeung; D. G. Patry; R. K. Zabad; P. E. Stenerson	2009	Glatiramer acetate in combination with minocycline in patients with relapsing-remitting multiple sclerosis: Results of a Canadian, multicenter, double-blind, placebo-controlled trial	10.1177/1352458509106779
M. Markianos; G. Koutsis; M. E. Evangelopoulos; D. Mandellos; G. Karahalios; C. Sfagos	2009	Relationship of CSF neurotransmitter metabolite levels to disease severity and disability in multiple sclerosis	10.1111/j.1471-4159.2008.05750.x
A. Conte; D. Lenzi; V. Frasca; F. Gilio; E. Giacomelli; M. Gabriele; C. M. Bettolo; E. Iacovelli; P. Pantano; C. Pozzilli; M. Inghilleri	2009	Intracortical excitability in patients with relapsing-remitting and secondary progressive multiple sclerosis	10.1007/s00415-009-5047-0
T. K. Yoldas; H. D. Keklikoglu; Ö. Zengin; E. B. Solak; S. Keskin	2010	Relation of Serum Uric Acid Level with Cognitive Functions and Number of Plaques in Patients with Relapsing-Remitting Multiple Sclerosis	10.4274/npa.y5720
M. Pardini; L. Bonzano; G. L. Mancardi; L. Roccatagliata	2010	Frontal Networks Play a Role in Fatigue Perception in Multiple Sclerosis	10.1037/a0019585
K. Konstantopoulos; M. Vikelis; J. A. Seikel; D. D. Mitsikostas	2010	The existence of phonatory instability in multiple sclerosis: An acoustic and electroglottographic study	10.1007/s10072-009-0170-3
H. D. Keklikoğlu; T. K. Yoldaş; O. Zengin; E. B. Solak; S. Keskin	2010	Cognitive impairment in patients with early relapsing-remitting multiple sclerosis	
S. M. Gold; K. C. Kern; M. F. O'Connor; M. J. Montag; A. Kim; Y. S. Yoo; B. S. Giesser; N. L. Sicotte	2010	Smaller cornu ammonis 23/dentate gyrus volumes and elevated cortisol in multiple sclerosis patients with depressive symptoms	10.1016/j.biopsych.2010.04.025
M. Calabrese; F. Rinaldi; P. Grossi; I. Mattisi; V. Bernardi; A. Favaretto; P. Perini; P. Gallo	2010	Basal ganglia and frontal/parietal cortical atrophy is associated with fatigue in relapsing-remitting multiple sclerosis	10.1177/1352458510376405
A. K. Andreasen; J. Jakobsen; L. Soerensen; H. Andersen; T. Petersen; C. R. Bjarkam; J. Ahdidan	2010	Regional brain atrophy in primary fatigued patients with multiple sclerosis	10.1016/j.neuroimage.2009.12.118
M. P. Amato; E. Portaccio; B. Goretti; V. Zipoli; A. Iudice; D. D. Pina; G. Malentacchi; S. Sabatini; P. Annunziata; M. Falcini; M. Mazzoni; M. Mortilla; C. Fonda; N. De Stefano	2010	Relevance of cognitive deterioration in early relapsing-remitting MS: A 3-year follow-up study	10.1177/1352458510380089

M. Togha; S. A. Karvigh; M. Nabavi; N. B. Moghadam; M. H. Harirchian; M. A. Sahraian; A. Enzevaei; A. Nourian; H. Ghanaati; K. Firouznia; A. Jannati; M. Shekiba	2010	Simvastatin treatment in patients with relapsing-remitting multiple sclerosis receiving interferon beta 1a: A double-blind randomized controlled trial	10.1177/1352458510369147
J. Somerfield; G. A. Hill-Cawthorne; A. Lin; M. S. Zandi; C. McCarthy; J. L. Jones; M. Willcox; D. Shaw; S. A. J. Thompson; A. S. Compston; G. Hale; H. Waldmann; A. J. Coles	2010	A novel strategy to reduce the immunogenicity of biological therapies	10.4049/jimmunol.1000422
G. M. Remington; K. Treadaway; T. Frohman; A. Salter; O. Stuve; M. K. Racke; K. Hawker; F. Agosta; M. P. Sormani; M. Filippi; E. M. Frohman	2010	A one-year prospective, randomized, placebo-controlled, quadruple-blinded, phase II safety pilot trial of combination therapy with interferon beta-1a and mycophenolate mofetil in early relapsing-remitting multiple sclerosis (TIME MS)	10.1177/1756285609355851
A. T. Reder; G. C. Ebers; A. Traboulsee; D. Li; D. Langdon; D. S. Goodin; T. Bogumil; K. Beckmann; A. Konieczny	2010	Cross-sectional study assessing long-term safety of interferon-β-1b for relapsing-remitting MS	10.1212/WNL.0b013e3181e240d0
F. Patti; M. Amato; S. Bastianello; L. Caniatti; E. Di Monte; P. Ferrazza; B. Goretti; P. Gallo; V. Brescia Morra; S. Lo Fermo; O. Picconi; M. Tola; M. Trojano	2010	Effects of immunomodulatory treatment with subcutaneous interferon beta-1a oncognitive decline in mildly disabled patients with relapsing-remitting multiple sclerosis	10.1177/1352458509350309
L. Kappos; E. W. Radue; P. O'Connor; C. Polman; R. Hohlfeld; P. Calabresi; K. Selmaj; C. Agoropoulou; M. Leyk; L. Zhang-Auberson; P. Burtin	2010	A placebo-controlled trial of oral fingolimod in relapsing multiple sclerosis	10.1056/NEJMoa0909494
S. Gupta; R. Varadarajulu; R. K. Ganjoo	2010	Beta-interferons in multiple sclerosis: A single center experience in India	10.4103/0972-2327.64624
E. M. Frohman; G. Cutter; H. Gao; A. Salter; G. Remington; A. Conger; K. Treadaway; T. C. Frohman; A. Bates; O. Stuve; B. M. Greenberg; H. Rossman; B. Weinstock Guttman; E. Lindzen; J. E. Durfee; E. Carl; M. G. Dwyer; A. Shah; J. L. Cox; M. K. Racke; R. Zivadinov	2010	A randomized, blinded, parallel-group, pilot trial of mycophenolate mofetil (CellCept) compared with interferon beta-1a (Avonex) in patients with relapsing-remitting multiple sclerosis	10.1177/1756285609353354
J. A. Cohen; F. Barkhof; G. Comi; H. P. Hartung; B. O. Khatri; X. Montalban; J. Pelletier; R. Capra; P. Gallo; G. Izquierdo; K. Tiel-Wilck; A. De Vera; J. Jin; T. Stites; S. Wu; S. Aradhye; L. Kappos	2010	Oral fingolimod or intramuscular interferon for relapsing multiple sclerosis	10.1056/NEJMoa0907839

D. Bîcu; A. Bondari; F. Trifan	2010	Features of diagnosis and treatment in familial multiple sclerosis	
F. Barkhof; H. E. Hulst; J. Drulović; B. M. J. Uitdehaag; K. Matsuda; R. Landin	2010	Ibudilast in relapsing-remitting multiple sclerosis: A neuroprotectant?	10.1212/WNL.0b013e3181d7d651
E. Arroyo; C. Grau; C. Ramo; J. Parra; O. Sánchez-Soliño	2010	Global adherence project to disease-modifying therapies in patients with relapsing multiple sclerosis: 2-year interim results	10.1016/j.nrl.2010.01.009
O. Yaldizli; S. Glassl; D. Sturm; A. Papadopoulou; A. Gass; B. Tettenborn; N. Putzki	2011	Fatigue and progression of corpus callosum atrophy in multiple sclerosis	10.1007/s00415-011-6091-0
F. Morgante; V. Dattola; D. Crupi; M. Russo; V. Rizzo; M. F. Ghilardi; C. Terranova; P. Girlanda; A. Quartarone	2011	Is central fatigue in multiple sclerosis a disorder of movement preparation?	10.1007/s00415-010-5742-x
M. Jehna; C. Langkammer; M. Wallner-Blazek; C. Neuper; M. Loitfelder; S. Ropele; S. Fuchs; M. Khalil; A. Pluta-Fuerst; F. Fazekas; C. Enzinger	2011	Cognitively preserved MS patients demonstrate functional differences in processing neutral and emotional faces	10.1007/s11682-011-9128-1
B. K. Tsang; R. Macdonell	2011	Multiple sclerosis: Diagnosis, management and prognosis	
M. B. Rietberg; E. E. H. Van Wegen; B. M. J. Uitdehaag; G. Kwakkel	2011	The association between perceived fatigue and actual level of physical activity in multiple sclerosis	10.1177/1352458511407102
M. Postal; L. T. L. Costallat; S. Appenzeller	2011	Neuropsychiatric manifestations in systemic lupus erythematosus: Epidemiology, pathophysiology and management	10.2165/11591670-000000000-00000
M. A. F. Nada; S. M. A. El-Mawella; H. A. Bayoumy; M. N. I. El Sirafy	2011	Personality trait and Coping strategies in multiple sclerosis: Neuropsychological and radiological correlation	
A. Miravalle; R. Jensen; R. P. Kinkel	2011	Immune reconstitution inflammatory syndrome in patients with multiple sclerosis following cessation of natalizumab therapy	10.1001/archneurol.2010.257
G. Kiy; P. Lehmann; H. K. Hahn; P. Eling; A. Kastrup; H. Hildebrandt	2011	Decreased hippocampal volume, indirectly measured, is associated with depressive symptoms and consolidation deficits in multiple sclerosis	10.1177/1352458511403530
B. Khatri; F. Barkhof; G. Comi; H. P. Hartung; L. Kappos; X. Montalban; J. Pelletier; T. Stites; S. Wu; F. Holdbrook; L. Zhang-Auberson; G. Francis; J. A. Cohen	2011	Comparison of fingolimod with interferon beta-1a in relapsing-remitting multiple sclerosis: A randomised extension of the TRANSFORMS study	10.1016/S1474-4422(11)70099-0
D. R. Jeffery; C. E. Markowitz; A. T. Reder; B. Weinstock-Guttman; K. Tobias	2011	Fingolimod for the treatment of relapsing multiple sclerosis	10.1586/ern.10.193

A. Javed; B. Soliven	2011	Fingolimod: A novel oral immunomodulatory treatment for multiple sclerosis	10.2217/fnl.11.15
S. Horowski; U. K. Zettl; R. Benecke; U. Walter	2011	Sonographic basal ganglia alterations are related to non-motor symptoms in multiple sclerosis	10.1007/s00415-010-5707-0
L. Grau-López; S. Sierra; E. Martínez-Cáceres; C. Ramo-Tello	2011	Analysis of the pain in multiple sclerosis patients	10.1016/j.nrl.2010.07.014
B. A. C. Cree; W. H. Stuart; C. S. Tornatore; D. R. Jeffery; A. L. Pace; C. H. Cha	2011	Efficacy of Natalizumab therapy in patients of African descent with relapsing multiple sclerosis: Analysis of AFFIRM and SENTINEL data	10.1001/archneurol.2011.45
S. Belachew; R. Phan-Ba; E. Bartholomé; V. Delvaux; I. Hansen; P. Calay; K. E. Hafsi; G. Moonen; L. Tshibanda; M. Vokaer	2011	Natalizumab induces a rapid improvement of disability status and ambulation after failure of previous therapy in relapsing-remitting multiple sclerosis	10.1111/j.1468-1331.2010.03112.x
I. Specogna; F. Casagrande; A. Lorusso; M. Catalan; A. Gorian; L. Zugna; R. Longo; M. Zorzon; M. Naccarato; G. Pizzolato; M. Ukmar; M. A. Cova	2012	Functional MRI during the execution of a motor task in patients with multiple sclerosis and fatigue	10.1007/s11547-012-0845-3
M. A. Rocca; M. Absinta; P. Valsasina; M. Copetti; D. Caputo; G. Comi; M. Filippi	2012	Abnormal cervical cord function contributes to fatigue in multiple sclerosis	10.1177/1352458512440516
S. Vucic; T. Burke; K. Lenton; S. Ramanathan; L. Gomes; C. Yannikas; M. C. Kiernan	2012	Cortical dysfunction underlies disability in multiple sclerosis	10.1177/1352458511424308
D. M. Sobieraj; C. I. Coleman	2012	Dimethyl fiimarate: A fumaric acid ester under investigation for the treatment of relapsing-remitting multiple sclerosis	
T. Saida; Y. Itoyama; K. Tashiro; J. I. Kira; Q. Hao	2012	Intramuscular interferon beta-1a is effective in Japanese patients with relapsing-remitting multiple sclerosis: A pre-treatment versus treatment comparison study of gadolinium-enhanced MRI brain lesions	10.1177/1352458512442261
E. Z. Papadaki; V. C. Mastorodemos; E. Z. Amanakis; K. C. Tsekouras; A. E. Papadakis; N. D. Tsavalas; P. G. Simos; A. H. Karantanas; A. Plaitakis; T. G. Maris	2012	White matter and deep gray matter hemodynamic changes in multiple sclerosis patients with clinically isolated syndrome	10.1002/mrm.24194
S. Nafissi; A. Azimi; A. Amini-Harandi; S. Salami; M. A. Shahkarami; R. Heshmat	2012	Comparing efficacy and side effects of a weekly intramuscular biogeneric/biosimilar interferon beta-1a with Avonex in relapsing remitting multiple sclerosis: A double blind randomized clinical trial	10.1016/j.clineuro.2012.02.039

D. C. Mohr; J. Lovera; T. Brown; B. Cohen; T. Neylan; R. Henry; J. Siddique; L. Jin; D. Daikh; D. Pelletier	2012	A randomized trial of stress management for the prevention of new brain lesions in MS	10.1212/WNL.0b013e3182616ff9
D. H. Miller; T. Weber; R. Grove; C. Wardell; J. Horrigan; O. Graff; G. Atkinson; P. Dua; T. Yousry; D. MacManus; X. Montalban	2012	Firategrast for relapsing remitting multiple sclerosis: A phase 2, randomised, double-blind, placebo-controlled trial	10.1016/S1474-4422(11)70299-X
T. Gabelić; I. Adamec; A. Mrden; M. Radoš; V. V. Brinar; M. Habek	2012	Psychotic reaction as a manifestation of multiple sclerosis relapse treated with plasma exchange	10.1007/s10072-011-0712-3
K. R. Edwards; W. A. Goodman; C. Y. Ma	2012	Improvement of neuropsychological function in cognitively impaired multiple sclerosis patients treated with natalizumab: A preliminary study	10.7224/1537-2073-14.2.100
J. C. Brooks	2012	Assessing spinal cord function in multiple sclerosis with functional neuroimaging: insights and limitations	10.1177/1352458512450357
A. Baumgartner; O. Stich; S. Rauer	2012	Clinical and radiological disease reactivation after cessation of long-term therapy with Natalizumab	10.3109/00207454.2011.622452
M. P. Amato; B. Hakiki; B. Goretti; F. Rossi; M. L. Stromillo; A. Giorgio; M. Roscio; A. Ghezzi; L. Guidi; M. L. Bartolozzi; E. Portaccio; N. De Stefano	2012	Association of MRI metrics and cognitive impairment in radiologically isolated syndromes	10.1212/WNL.0b013e31824528c9
A. S. Abd El-Ghaffa; M. A. Aidaros; A. A. M. Abdel Ghani; A. M. Zaitoun; E. S. M. Abd El-Ghany; M. M. Badr	2012	Clinical, immunological and radiological correlations in multiple sclerosis patients	
L. Tomasevic; G. Zito; P. Pasqualetti; M. Filippi; D. Landi; A. Ghazaryan; D. Lupoi; C. Porcaro; F. Bagnato; P. Rossini; F. Tecchio	2013	Cortico-muscular coherence as an index of fatigue in multiple sclerosis	10.1177/1352458512452921
E. Szabadi	2013	Functional neuroanatomy of the central noradrenergic system	10.1177/0269881113490326
C. E. Schwartz; B. R. Quaranto; B. C. Healy; R. H. Benedict; T. L. Vollmer	2013	Cognitive Reserve and Symptom Experience in Multiple Sclerosis: A Buffer to Disability Progression Over Time?	10.1016/j.apmr.2013.05.009
A. Papadopoulou; N. Müller-Lenke; Y. Naegelin; G. Kalt; K. Bendfeldt; P. Kuster; M. Stoecklin; A. Gass; T. Sprenger; E. W. Radue; L. Kappos; I. K. Penner	2013	Contribution of cortical and white matter lesions to cognitive impairment in multiple sclerosis	10.1177/1352458513475490
C. Fazekas; M. Khalil; C. Enzinger; F. Matzer; S. Fuchs; F. Fazekas	2013	No impact of adult attachment and temperament on clinical variability in patients with clinically isolated syndrome and early multiple sclerosis	10.1016/j.clineuro.2012.05.022

N. Derache; B. Grassiot; F. Mézenge; A. Emmanuelle Dugué; B. Desgranges; J. M. Constans; G. L. Defer	2013	Fatigue is associated with metabolic and density alterations of cortical and deep gray matter in Relapsing-Remitting-Multiple Sclerosis patients at the earlier stage of the disease: A PET/MR study	10.1016/j.msard.2013.03.005
A. J. Cruz Gómez; N. Ventura Campos; A. Belenguer; C. Ávila; C. Forn	2013	Regional Brain Atrophy and Functional Connectivity Changes Related to Fatigue in Multiple Sclerosis	10.1371/journal.pone.0077914
M. Yildiz; B. Tettenborn; S. Borgwardt	2013	Trajectory of fatigue severity in natalizumab treated multiple sclerosis patients	10.1016/j.clineuro.2012.08.039
S. Vanotti; E. V. Cores; B. Eizaguirre; L. Melamud; R. Rey; A. Villa	2013	Cognitive performance of neuromyelitis optica patients: comparison with multiple sclerosis	10.1590/0004-282x20130038
K. Selmaj; D. K. B. Li; H. P. Hartung; B. Hemmer; L. Kappos; M. S. Freedman; O. Stüve; P. Rieckmann; X. Montalban; T. Ziemssen; L. Z. Auberson; H. Pohlmann; F. Mercier; F. Dahlke; E. Wallström	2013	Siponimod for patients with relapsing-remitting multiple sclerosis (BOLD): An adaptive, dose-ranging, randomised, phase 2 study	10.1016/S1474-4422(13)70102-9
C. E. Markowitz	2013	Multiple Sclerosis Update	
M. Lacy; M. Hauser; N. Pliskin; S. Assuras; M. O. Valentine; A. Reder	2013	The effects of long-term interferon-beta-1b treatment on cognitive functioning in multiple sclerosis: A 16-year longitudinal study	10.1177/1352458513485981
S. Inaloo; S. Haghbin	2013	Multiple sclerosis in children	
M. J. Gil Moreno; M. Cerezo García; R. Marasescu; A. Pinel González; L. López Álvarez; Y. Aladro Benito	2013	Neuropsychological syndromes in multiple sclerosis	10.7334/psicothema2012.308
F. Fazekas; O. Bajenaru; T. Berger; T. H. Fabjan; A. H. Ledinek; G. Jakab; S. Komoly; T. Kobys; J. Kraus; E. Kurča; T. Kyriakides; L. Lisý; I. Milanov; T. Nehrych; S. Moskovko; P. Panayiotou; S. S. Jazbec; L. Sokolova; R. Taláb; L. Traykov; P. Turčáni; K. Vass; N. Vella; N. Voloshyná; E. Havrdová	2013	How does fingolimod (gilenya®) fit in the treatment algorithm for highly active relapsing-remitting multiple sclerosis?	10.3389/fneur.2013.00010
R. H. Benedict; B. Weinstock-Guttmam; K. Marr; V. Valnarov; C. Kennedy; E. Carl; C. Brooks; D. Hojnacki; R. Zivadinov	2013	Chronic cerebrospinal venous insufficiency is not associated with cognitive impairment in multiple sclerosis	10.1186/1741-7015-11-167

H. Beckerman; L. J. M. Blikman; M. Heine; A. Malekzadeh; C. E. Teunissen; J. B. J. Bussmann; G. Kwakkel; J. van Meeteren; V. de Groot; M. Looijmans; S. A. Sanches; J. Dekker; E. H. Collette; B. W. van Oosten; M. A. Blankenstein; I. C. J. M. Eijssen; M. Rietberg; I. G. L. van de Port; E. Lindeman; H. J. Stam; R. Q. Hintzen; H. G. A. Hacking; S. T. F. M. Frequin; E. Hoogervorst; B. A. de Jong; J. H. Knoop; G. Bleijenberg; F. A. J. de Laat; G. J. Aarts	2013	The effectiveness of aerobic training, cognitive behavioural therapy, and energy conservation management in treating MS-related fatigue: The design of the TREFAMS-ACE programme	10.1186/1745-6215-14-250
R. Alroughani; J. Al Hashel; A. Thussu; S. F. Ahmed	2013	Use of natalizumab in patients with active relapsing-remitting multiple sclerosis in Kuwait	10.1159/000351568
G. Zito; E. Luders; L. Tomasevic; D. Lupoi; A. W. Toga; P. M. Thompson; P. M. Rossini; M. M. Filippi; F. Tecchio	2014	INTER-HEMISPHERIC FUNCTIONAL CONNECTIVITY CHANGES WITH CORPUS CALLOSUM MORPHOLOGY IN MULTIPLE SCLEROSIS	10.1016/j.neuroscience.2014.01.039
F. Zhou; Y. Zhuang; H. Gong; B. Wang; X. Wang; Q. Chen; L. Wu; H. Wan	2014	Altered inter-subregion connectivity of the default mode network in relapsing remitting multiple sclerosis: A functional and structural connectivity study	10.1371/journal.pone.0101198
M. A. Wojtowicz; Y. Ishigami; E. L. Mazerolle; J. D. Fisk	2014	Stability of intraindividual variability as a marker of neurologic dysfunction in relapsing remitting multiple sclerosis	10.1080/13803395.2014.903898
A. E. Williams; J. T. Vietri; G. Isherwood; A. Flor	2014	Symptoms and Association with Health Outcomes in Relapsing-Remitting Multiple Sclerosis: Results of a US Patient Survey	10.1155/2014/203183
T. Štecková; P. Hluštík; V. Sládková; F. Odstrčil; J. Mareš; P. Kaňovský	2014	Thalamic atrophy and cognitive impairment in clinically isolated syndrome and multiple sclerosis	10.1016/j.jns.2014.04.026
Y. Shen; L. Bai; Y. Gao; F. Cui; Z. Tan; Y. Tao; C. Sun; L. Zhou	2014	Depressive symptoms in multiple sclerosis from an in vivo study with TBSS	10.1155/2014/148465
S. A. Mohamed; O. El-Deib	2014	Depressive symptoms as a predictor of outcome in patients with multiple sclerosis	10.1097/01.XME.0000438127.40735.0c
L. Hofstetter; Y. Naegelin; L. Filli; P. Kuster; S. Traud; R. Smieskova; N. Mueller-Lenke; L. Kappos; A. Gass; T. Sprenger; I. K. Penner; T. E. Nichols; H. Vrenken; F. Barkhof; C. Polman; E. W. Radue; S. J. Borgwardt; K. Bendfeldt	2014	Progression in disability and regional grey matter atrophy in relapsing-remitting multiple sclerosis	10.1177/1352458513493034
H. Hildebrandt; P. Eling	2014	A longitudinal study on fatigue, depression, and their relation to neurocognition in multiple sclerosis	10.1080/13803395.2014.903900

M. N. Burns; E. Nawacki; M. J. Kwasny; D. Pelletier; D. C. Mohr	2014	Do positive or negative stressful events predict the development of new brain lesions in people with multiple sclerosis?	10.1017/S0033291713000755
M. Yildiz; B. Tettenborn; E. W. Radue; K. Bendfeldt; S. Borgwardt	2014	Association of cognitive impairment and lesion volumes in multiple sclerosis - A MRI study	10.1016/j.clineuro.2014.09.019
O. Yaldizli; I. K. Penner; K. Frontzek; Y. Naegelin; M. Amann; A. Papadopoulou; T. Sprenger; J. Kuhle; P. Calabrese; E. W. Radü; L. Kappos; A. Gass	2014	The relationship between total and regional corpus callosum atrophy, cognitive impairment and fatigue in multiple sclerosis patients	10.1177/1352458513496880
L. Wu; F. Q. Zhou; Y. Zhang; H. F. Guan; H. M. Kuang; H. H. Gong	2014	Voxel-based resting state fMRI observation on changes of degree centrality of whole brain in patients with relapsing remitting multiple sclerosis	
J. F. Sumowski; V. M. Leavitt	2014	Body temperature is elevated and linked to fatigue in relapsing-remitting multiple sclerosis, even without heat exposure	10.1016/j.apmr.2014.02.004
M. B. Rietberg; E. E. H. Van Wegen; I. C. J. M. Eyssen; G. Kwakkel	2014	Effects of multidisciplinary rehabilitation on chronic fatigue in multiple sclerosis: A randomized controlled trial	10.1371/journal.pone.0107710
L. Parisi; M. A. Rocca; F. Mattioli; M. Copetti; R. Capra; P. Valsasina; C. Stampatori; M. Filippi	2014	Changes of brain resting state functional connectivity predict the persistence of cognitive rehabilitation effects in patients with multiple sclerosis	10.1177/1352458513505692
M. Pardini; M. Bergamino; G. Bommarito; L. Bonzano; G. L. Mancardi; L. Roccatagliata	2014	Structural Correlates of Subjective and Objective Memory Performance in Multiple Sclerosis	10.1002/hipo.22237
F. D. Lublin; J. D. Bowen; J. Huddlestone; M. Kremenchutzky; A. Carpenter; J. R. Corboy; M. S. Freedman; L. Krupp; C. Paulo; R. J. Hariri; S. A. Fischkoff	2014	Human placenta-derived cells (PDA-001) for the treatment of adults with multiple sclerosis: a randomized, placebo-controlled, multiple-dose study	10.1016/j.msard.2014.08.002
M. López-Ruiz; J. L. Ruiz-Sandoval; N. S. Barroso-Rodríguez; C. G. Cantú-Brito; J. A. Violante-Villanueva; A. Molina-Pérez; J. Revilla-Beltri	2014	Open label, extension-of-PRO-3209 trial to assess efficacy and safety of Probioglat® (glatiramer acetate) in Mexican patients with relapsing-remitting multiple sclerosis. Interim report of the first 12 months of treatment (Study PRO-4109)	
P. K. Coyle; B. A. Cohen; T. Leist; C. Markowitz; M. Oleen-Burkey; M. Schwartz; M. J. Tullman; H. Zwibel	2014	Therapy optimization in multiple sclerosis: A prospective observational study of therapy compliance and outcomes	10.1186/1471-2377-14-49
P. K. Coyle	2014	Current evaluation of alemtuzumab in multiple sclerosis	10.1517/14712598.2014.866084

P. A. Calabresi; B. C. Kieseier; D. L. Arnold; L. J. Balcer; A. Boyko; J. Pelletier; S. Liu; Y. Zhu; A. Seddighzadeh; S. Hung; A. Deykin	2014	Pegylated interferon beta-1a for relapsing-remitting multiple sclerosis (ADVANCE): A randomised, phase 3, double-blind study	10.1016/S1474-4422(14)70068-7
G. Bonnier; A. Roche; D. Romascano; S. Simioni; D. Meskaldji; D. Rotzinger; Y. C. Lin; G. Menegaz; M. Schluep; R. Du Pasquier; T. J. Sumpf; J. Frahm; J. P. Thiran; G. Krueger; C. Granziera	2014	Advanced MRI unravels the nature of tissue alterations in early multiple sclerosis	10.1002/acn3.68
M. Stangel; I. K. Penner; B. A. Kallmann; C. Lukas; B. C. Kieseier	2015	Towards the implementation of 'no evidence of disease activity' in multiple sclerosis treatment: The multiple sclerosis decision model	10.1177/1756285614560733
K. Pierzchala; M. Adamczyk-Sowa; P. Dobrakowski; K. Kubicka-Baczyk; N. Niedziela; P. Sowa	2015	Demographic characteristics of MS patients in Poland's upper Silesia region	10.3109/00207454.2014.937002
M. Pardini; L. Bonzano; M. Bergamino; G. Bommarito; P. Feraco; A. Murugavel; M. Bove; G. Brichetto; A. Uccelli; G. Mancardi; L. Roccatagliata	2015	Cingulum bundle alterations underlie subjective fatigue in multiple sclerosis	10.1177/1352458514546791
G. O. Nygaard; K. B. Walhovd; P. Sowa; J. L. Chepkoech; A. Bjørnerud; P. Due-Tønnessen; N. I. Landrø; S. Damangir; G. Spulber; A. B. Storsve; M. K. Beyer; A. M. Fjell; E. G. Celius; H. F. Harbo	2015	Cortical thickness and surface area relate to specific symptoms in early relapsing-remitting multiple sclerosis	10.1177/1352458514543811
S. Nigro; L. Passamonti; R. Riccelli; N. Toschi; F. Rocca; P. Valentino; R. Nisticò; F. Fera; A. Quattrone	2015	Structural connectomic alterations in the limbic system of multiple sclerosis patients with major depression	10.1177/1352458514558474
V. M. Leavitt; E. De Meo; G. Riccitelli; M. A. Rocca; G. Comi; M. Filippi; J. F. Sumowski	2015	Elevated body temperature is linked to fatigue in an Italian sample of relapsing-remitting multiple sclerosis patients	10.1007/s00415-015-7863-8
K. C. Kern; S. M. Gold; B. Lee; M. Montag; J. Horsfall; M. F. O'Connor; N. L. Sicotte	2015	Thalamic-hippocampal-prefrontal disruption in relapsing-remitting multiple sclerosis	10.1016/j.nicl.2014.12.015
C. Finke; J. Schlichting; S. Papazoglou; M. Scheel; A. Freing; C. Soemmer; L. M. Pech; A. Pajkert; C. Pfüller; J. T. Wuerfel; C. J. Ploner; F. Paul; A. U. Brandt	2015	Altered basal ganglia functional connectivity in multiple sclerosis patients with fatigue	10.1177/1352458514555784
A. Ernst; V. Noblet; E. Denkova; F. Blanc; J. de Seze; D. Gounot; L. Manning	2015	Functional cerebral changes in multiple sclerosis patients during an autobiographical memory test	10.1080/09658211.2014.955805

L. Debernard; T. R. Melzer; S. Alla; J. Eagle; S. Van Stockum; C. Graham; J. R. Osborne; J. C. Dalrymple-Alford; D. H. Miller; D. F. Mason	2015	Deep grey matter MRI abnormalities and cognitive function in relapsing-remitting multiple sclerosis	10.1016/j.pscychresns.2015.10.004
G. Bonnier; A. Roche; D. Romascano; S. Simioni; D. E. Meskaldji; D. Rotzinger; Y. C. Lin; G. Menegaz; M. Schluep; R. Du Pasquier; T. J. Sumpf; J. Frahm; J. P. Thiran; G. Krueger; C. Granziera	2015	Multicontrast MRI Quantification of Focal Inflammation and Degeneration in Multiple Sclerosis	10.1155/2015/569123
V. Biberacher; C. C. Boucard; P. Schmidt; C. Engl; D. Buck; A. Berthele; M. M. Hoshi; C. Zimmer; B. Hemmer; M. Mühlau	2015	Atrophy and structural variability of the upper cervical cord in early multiple sclerosis	10.1177/1352458514546514
S. Tauhid; R. X. Chu; R. Sasane; B. I. Glanz; M. Neema; J. R. Miller; G. Kim; J. E. Signorovitch; B. C. Healy; T. Chitnis; H. L. Weiner; R. Bakshi	2015	Brain MRI lesions and atrophy are associated with employment status in patients with multiple sclerosis	10.1007/s00415-015-7853-x
M. Sundgren; Å. Wahlin; L. Maurex; T. Brismar	2015	Event related potential and response time give evidence for a physiological reserve in cognitive functioning in relapsing-remitting multiple sclerosis	10.1016/j.jns.2015.06.025
G. O. Nygaard; E. G. Celius; S. A. De Rodez Benavent; P. Sowa; M. W. Gustavsen; A. M. Fjell; N. I. Landrø; K. B. Walhovd; H. F. Harbo; O. Aktas	2015	A longitudinal study of disability, cognition and gray matter atrophy in early multiple sclerosis patients according to evidence of disease activity	10.1371/journal.pone.0135974
D. Nunnari; M. C. De Cola; G. D'Aleo; C. Rifici; M. Russo; E. Sessa; P. Bramanti; S. Marino	2015	Impact of Depression, Fatigue, and Global Measure of Cortical Volume on Cognitive Impairment in Multiple Sclerosis	10.1155/2015/519785
M. Muto; M. Mori; Y. Sato; A. Uzawa; S. Masuda; T. Uchida; S. Kuwabara	2015	Current symptomatology in multiple sclerosis and neuromyelitis optica	10.1111/ene.12566
A. Malekzadeh; W. Van De Geer-Peeters; V. De Groot; C. Elisabeth Teunissen; H. Beckerman; T. A. Study Group	2015	Fatigue in patients with multiple sclerosis: Is it related to pro- and anti-inflammatory cytokines?	10.1155/2015/758314
L. Kappos; P. O'Connor; E. W. Radue; C. Polman; R. Hohlfeld; K. Selmaj; S. Ritter; R. Schlosshauer; P. Von Rosenstiel; L. Zhang-Auberson; G. Francis	2015	Long-term effects of fingolimod in multiple sclerosis	10.1212/WNL.0000000000001462
K. Hanken; P. Eling; A. Kastrup; J. Klein; H. Hildebrandt	2015	Integrity of hypothalamic fibers and cognitive fatigue in multiple sclerosis	10.1016/j.msard.2014.11.006
A. Damasceno; B. P. Damasceno; F. Cendes	2015	Subclinical MRI disease activity influences cognitive performance in MS patients	10.1016/j.msard.2015.01.006

T. Alleman; B. van Oosten	2015	Multiple sclerosis in general practice	10.1007/s12445-015-0137-y
M. M. Allam; M. M. El-Hamady; A. A. Morad; M. K. Faheem; B. A. E. Morsi	2015	Some neuropsychiatric and MRI manifestations in patients with multiple sclerosis	10.1097/01.XME.0000458885.02819.92
F. Zhou; H. Gong; Q. Chen; B. Wang; Y. Peng; Y. Zhuang; C. S. Zee	2016	Intrinsic functional plasticity of the thalamocortical system in minimally disabled patients with relapsing-remitting multiple sclerosis	10.3389/fnhum.2016.00002
F. Yousuf; G. Kim; S. Tauhid; B. I. Glanz; R. Chu; S. Tummala; B. C. Healy; R. Bakshi	2016	The contribution of cortical lesions to a composite MRI scale of disease severity in multiple sclerosis	10.3389/fneur.2016.00099
M. Yildiz; F. Brugger; N. Kästle; B. Tettenborn	2016	Neurocognitive impairment is associated with corpus callosum atrophy in multiple sclerosis	10.1016/j.npbr.2016.03.001
Ö. Yaldizli; I. K. Penner; T. Yonekawa; Y. Naegelin; J. Kuhle; M. Pardini; D. T. Chard; C. Stippich; J. I. Kira; K. Bendfeldt; M. Amann; E. W. Radue; L. Kappos; T. Sprenger	2016	The association between olfactory bulb volume, cognitive dysfunction, physical disability and depression in multiple sclerosis	10.1111/ene.12891
L. Wu; Y. Zhang; F. Zhou; L. Gao; L. He; X. Zeng; H. Gong	2016	Altered intra- and interregional synchronization in relapsing-remitting multiple sclerosis: a resting-state fMRI study	10.2147/ndt.S98962
J. Wilting; H. O. Rolfsnes; H. Zimmermann; M. Behrens; V. Fleischer; F. Zipp; A. Gröger	2016	Structural correlates for fatigue in early relapsing remitting multiple sclerosis	10.1007/s00330-015-3857-2
S. Sevim	2016	Relapses in multiple sclerosis: Definition, pathophysiology, features, imitators, and treatment	10.4274/tnd.75318
M. A. Rocca; A. Meani; G. C. Riccitelli; B. Colombo; M. Rodegher; A. Falini; G. Comi; M. Filippi	2016	Abnormal adaptation over time of motor network recruitment in multiple sclerosis patients with fatigue	10.1177/1352458515614407
R. Riccelli; L. Passamonti; A. Cerasa; S. Nigro; S. M. Cavalli; C. Chiriaco; P. Valentino; R. Nisticò; A. Quattrone	2016	Individual differences in depression are associated with abnormal function of the limbic system in multiple sclerosis patients	10.1177/1352458515606987
E. Pravatà; C. Zecca; C. Sestieri; M. Caulo; G. C. Riccitelli; M. A. Rocca; M. Filippi; A. Cianfoni; C. Gobbi	2016	Hyperconnectivity of the dorsolateral prefrontal cortex following mental effort in multiple sclerosis patients with cognitive fatigue	10.1177/1352458515625806
B. Nourbakhsh; C. Azevedo; J. Nunan-Saah; A. H. Maghzi; R. Spain; D. Pelletier; E. Waubant	2016	Longitudinal associations between brain structural changes and fatigue in early MS	10.1016/j.msard.2015.10.006
S. P. Hojjat; C. G. Cantrell; T. J. Carroll; R. Vitorino; A. Feinstein; L. Zhang; S. P. Symons; S. A. Morrow; L. Lee; P. O'Connor; R. I. Aviv	2016	Perfusion reduction in the absence of structural differences in cognitively impaired versus unimpaired RRMS patients	10.1177/1352458516628656

M. Gschwind; M. Hardmeier; D. Van De Ville; M. I. Tomescu; I. K. Penner; Y. Naegelin; P. Fuhr; C. M. Michel; M. Seeck	2016	Fluctuations of spontaneous EEG topographies predict disease state in relapsing-remitting multiple sclerosis	10.1016/j.nicl.2016.08.008
A. Ernst; M. Sourty; D. Roquet; V. Noblet; D. Gounot; F. Blanc; J. De Seze; L. Manning	2016	Functional and structural cerebral changes in key brain regions after a facilitation programme for episodic future thought in relapsing-remitting multiple sclerosis patients	10.1016/j.bandc.2016.03.007
A. Damasceno; B. P. Damasceno; F. Cendes	2016	Atrophy of reward-related striatal structures in fatigued MS patients is independent of physical disability	10.1177/1352458515599451
M. Cavallari; M. Palotai; B. I. Glanz; S. Egorova; J. C. Prieto; B. C. Healy; T. Chitnis; C. R. G. Guttmann	2016	Fatigue predicts disease worsening in relapsing-remitting multiple sclerosis patients	10.1177/1352458516635874
N. Bergsland; R. Zivadinov; M. G. Dwyer; B. Weinstock-Guttman; R. H. B. Benedict	2016	Localized atrophy of the thalamus and slowed cognitive processing speed in MS patients	10.1177/1352458515616204
A. Bisecco; G. Caiazzo; A. D'Ambrosio; R. Sacco; S. Bonavita; R. Docimo; M. Cirillo; E. Pagani; M. Filippi; F. Esposito; G. Tedeschi; A. Gallo	2016	Fatigue in multiple sclerosis: The contribution of occult white matter damage	10.1177/1352458516628331
F. Zhou; Y. Zhuang; H. Gong; J. Zhan; M. Grossman; Z. Wang	2016	Resting state brain entropy alterations in relapsing remitting multiple sclerosis	10.1371/journal.pone.0146080
W. H. Zaini; F. Giuliani; C. Beaulieu; S. Kalra; C. Hanstock	2016	Fatigue in Multiple Sclerosis: Assessing Pontine Involvement Using Proton MR Spectroscopic Imaging	10.1371/journal.pone.0149622
H. Stuke; K. Hanken; J. Hirsch; J. Klein; F. Wittig; A. Kastrup; H. Hildebrandt	2016	Cross-Sectional and Longitudinal Relationships between Depressive Symptoms and Brain Atrophy in MS Patients	10.3389/fnhum.2016.00622
P. S. Sørensen; F. Sellebjerg; J. Lycke; M. Färkkilä; A. Créange; C. G. Lund; M. Schluep; J. L. Frederiksen; E. Stenager; C. Pfleger; E. Garde; E. Kinnunen; K. Marhardt	2016	Minocycline added to subcutaneous interferon β-1a in multiple sclerosis: Randomized RECYCLINE study	10.1111/ene.12953
R. Riera; G. J. Porfírio; M. R. Torloni	2016	Alemtuzumab for multiple sclerosis	10.1002/14651858.CD011203.pub2
M. D. Ludwig; A. P. Turel; I. S. Zagon; P. J. McLaughlin	2016	Long-term treatment with low dose naltrexone maintains stable health in patients with multiple sclerosis	10.1177/2055217316672242
Y. Lu; J. Zhao; Q. Zhan	2016	Effect of interferon-β1α therapy on multiple sclerosis based on gadolinium-enhancing or active T2 magnetic resonance imaging outcomes: a meta-analysis	10.1080/01616412.2016.1214417

M. A. Horsfield; M. A. Rocca; E. Pagani; L. Storelli; P. Preziosa; R. Messina; F. Camesasca; M. Copetti; M. Filippi	2016	Estimating Brain Lesion Volume Change in Multiple Sclerosis by Subtraction of Magnetic Resonance Images	10.1111/jon.12344
D. M. Harrison; X. Li; H. Liu; C. K. Jones; B. Caffo; P. A. Calabresi; P. van Zijl	2016	Lesion Heterogeneity on High-Field Susceptibility MRI Is Associated with Multiple Sclerosis Severity	10.3174/ajnr.A4726
S. J. Greenberg; R. Zivadinov; P. Lee-Kwen; J. Sharma; M. Planter; M. Umhauer; N. Glenister; R. Bakshi	2016	Fludarabine add-on therapy in interferon-beta-treated patients with multiple sclerosis experiencing breakthrough disease	10.1177/1756285615626049
A. Damasceno; B. P. Damasceno; F. Cendes	2016	No evidence of disease activity in multiple sclerosis: Implications on cognition and brain atrophy	10.1177/1352458515604383
R. Conforti; M. Cirillo; A. Sardaro; G. Caiazzo; A. Negro; A. Paccone; R. Sacco; M. Sparaco; A. Gallo; L. Lavorgna; G. Tedeschi; S. Cirillo	2016	Dilated perivascular spaces and fatigue: is there a link? Magnetic resonance retrospective 3Tesla study	10.1007/s00234-016-1711-0
G. Bsteh; R. Ehling; A. Lutterotti; H. Hegen; F. Di Pauli; M. Auer; F. Deisenhammer; M. Reindl; T. Berger	2016	Long Term Clinical Prognostic Factors in Relapsing-Remitting Multiple Sclerosis: Insights from a 10-Year Observational Study	10.1371/journal.pone.0158978
J. C. Bertoglio; M. Baumgartner; R. Palma; E. Ciampi; C. Carcamo; D. D. Cáceres; G. Acosta-Jamett; J. L. Hancke; R. A. Burgos	2016	Andrographis paniculata decreases fatigue in patients with relapsing-remitting multiple sclerosis: A 12-month double-blind placebo-controlled pilot study	10.1186/s12883-016-0595-2
M. Russo; A. Calamuneri; A. Cacciola; L. Bonanno; A. Naro; V. Dattola; E. Sessa; M. Buccafusca; D. Milardi; P. Bramanti; R. S. Calabrò; G. Anastasi; A. Quartarone	2017	Neural correlates of fatigue in multiple sclerosis: A combined neurophysiological and neuroimaging approach (R1)	10.12871/00039829201735
S. Rossi; V. Studer; C. Motta; S. Polidoro; J. Perugini; G. Macchiarulo; A. M. Giovannetti; L. Pareja-Gutierrez; A. Calò; I. Colonna; R. Furlan; G. Martino; D. Centonze	2017	Neuroinflammation drives anxiety and depression in relapsing-remitting multiple sclerosis	10.1212/WNL.00000000000044 11
J. I. Rojas; F. Sanchez; L. Patrucco; J. Miguez; C. Besada; E. Cristiano	2017	Brain structural changes in patients in the early stages of multiple sclerosis with depression	10.1080/01616412.2017.129827 9
R. Righart; V. Biberacher; L. E. Jonkman; R. Klaver; P. Schmidt; D. Buck; A. Berthele; J. S. Kirschke; C. Zimmer; B. Hemmer; J. J. G. Geurts; M. Mühlau	2017	Cortical pathology in multiple sclerosis detected by the T1/T2-weighted ratio from routine magnetic resonance imaging	10.1002/ana.25020
F. Al-Hussain; N. Al-Salloum; N. Alazwary; J. Saeedi; S. Howaidi; A. Daif	2017	Depression, anxiety and stress severities in multiple sclerosis patients using injectable versus oral treatments	10.2217/cer-2016-0087

M. J. Fartaria; K. O'Brien; A. Sorega; G. Bonnier; A. Roche; P. Falkovskiy; G. Krueger; T. Kober; M. B. Cuadra; C. Granziera	2017	An Ultra-High Field Study of Cerebellar Pathology in Early Relapsing-Remitting Multiple Sclerosis Using MP2RAGE	10.1097/rli.0000000000000338
F. Vecchio; F. Miraglia; C. Porcaro; C. Cottone; A. Cancelli; P. M. Rossini; F. Tecchio	2017	Electroencephalography-Derived Sensory and Motor Network Topology in Multiple Sclerosis Fatigue	10.1177/1545968316656055
L. E. van den Akker; H. Beckerman; E. H. Collette; J. W. R. Twisk; G. Bleijenberg; J. Dekker; H. Knoop; V. de Groot	2017	Cognitive behavioral therapy positively affects fatigue in patients with multiple sclerosis: Results of a randomized controlled trial	10.1177/1352458517709361
S. H. Kim; K. Kwak; J. W. Hyun; A. Joung; S. H. Lee; Y. H. Choi; J. M. Lee; H. J. Kim	2017	Diffusion tensor imaging of normal-appearing white matter in patients with neuromyelitis optica spectrum disorder and multiple sclerosis	10.1111/ene.13321
S. J. Khoury; J. Rochon; L. Ding; M. Byron; K. Ryker; P. Tosta; W. Gao; M. S. Freedman; D. L. Arnold; P. H. Sayre; D. E. Smilek	2017	ACCLAIM: A randomized trial of abatacept (CTLA4-Ig) for relapsing-remitting multiple sclerosis	10.1177/1352458516662727
E. Kantorová; H. Poláček; M. Bittšanský; E. Baranovičová; P. Hnilicová; D. Čierny; Š. Sivák; V. Nosáľ; K. Zeleňák; E. Kurča	2017	Hypothalamic damage in multiple sclerosis correlates with disease activity, disability, depression, and fatigue	10.1080/01616412.2016.1275460
I. T. Hagström; R. Schneider; B. Bellenberg; A. Salmen; F. Weiler; O. Köster; R. Gold; C. Lukas	2017	Relevance of early cervical cord volume loss in the disease evolution of clinically isolated syndrome and early multiple sclerosis: a 2-year follow-up study	10.1007/s00415-017-8537-5
J. Gäartner; W. Brück; A. Weddige; H. Hummel; C. Norenberg; J. P. Bugge	2017	Interferon beta-1b in treatment-naïve paediatric patients with relapsing–remitting multiple sclerosis: Two-year results from the BETAPAEDIC study	10.1177/2055217317747623
A. M. Elkady; D. Cobzas; H. F. Sun; G. Blevins; A. H. Wilman	2017	Progressive Iron Accumulation Across Multiple Sclerosis Phenotypes Revealed by Sparse Classification of Deep Gray Matter	10.1002/jmri.25682
L. De Giglio; F. Marinelli; V. T. Barletta; V. A. Pagano; F. De Angelis; F. Fanelli; N. Petsas; P. Pantano; V. Tomassini; C. Pozzilli	2017	Effect on Cognition of Estroprogestins Combined with Interferon Beta in Multiple Sclerosis: Analysis of Secondary Outcomes from a Randomised Controlled Trial	10.1007/s40263-016-0401-0
S. Dániel; B. Tamás; D. Szekeres; F. Judit; Z. T. Kincses; R. Csilla; K. Matyas; K. Krisztián; J. Matolcsi; D. Zboznovits; B. Zita; L. Éva; V. László; B. Krisztina	2017	Prevalence of of cognitive impairment among Hungarian patients with relapsing-remitting multiple sclerosis and clinically isolated syndrome	10.1016/j.msard.2017.06.017
D. S. Conway; N. R. Thompson; J. A. Cohen	2017	Influence of hypertension, diabetes, hyperlipidemia, and obstructive lung disease on multiple sclerosis disease course	10.1177/1352458516650512

L. J. M. Blikman; J. van Meeteren; J. W. R. Twisk; F. A. J. de Laat; V. de Groot; H. Beckerman; H. J. Stam; J. B. J. Bussmann	2017	Effectiveness of energy conservation management on fatigue and participation in multiple sclerosis: A randomized controlled trial	10.1177/1352458517702751
E. Bernitsas; K. Yarraguntla; F. Bao; R. Sood; C. Santiago-Martinez; R. Govindan; O. Khan; N. Seraji-Bozorgzad	2017	Structural and neuronal integrity measures of fatigue severity in multiple sclerosis	10.3390/brainsci7080102
K. Yarraguntla; N. Seraji-Bozorgzad; S. Lichtman-Mikol; S. Razmjou; F. Bao; S. Sriwastava; C. Santiago-Martinez; O. Khan; E. Bernitsas	2018	Multiple Sclerosis Fatigue: A Longitudinal Structural MRI and Diffusion Tensor Imaging Study	10.1111/jon.12548
O. Svolgaard; K. W. Andersen; C. Bauer; K. H. Madsen; M. Blinkenberg; F. Selleberg; H. R. Siebner	2018	Cerebellar and premotor activity during a non-fatiguing grip task reflects motor fatigue in relapsing-remitting multiple sclerosis	10.1371/journal.pone.0201162
P. Puz; A. Lasek-Bal; A. Steposz; K. Bartoszek	2018	Effect of comorbidities on the course of multiple sclerosis	10.1016/j.clineuro.2018.02.014
A. Pokryszko-Dragan; A. Banaszek; M. Nowakowska-Kotas; K. Jeżowska-Jurczyk; E. Dziadkowiak; E. Gruszka; M. Zagrajek; M. Bilińska; S. Budrewicz; M. Sąsiadek; J. Bladowska	2018	Diffusion tensor imaging findings in the multiple sclerosis patients and their relationships to various aspects of disability	10.1016/j.jns.2018.06.007
C. Lebrun; O. H. Kantarci; A. Siva; D. Pelletier; D. T. Okuda	2018	Anomalies Characteristic of Central Nervous System Demyelination: Radiologically Isolated Syndrome	10.1016/j.ncl.2017.08.004
D. Iancheva; A. G. Trenova; K. Terziyski; S. Kandilarova; S. Mantarova	2018	Translational validity of PASAT and the effect of fatigue and mood in patients with relapsing remitting MS: A functional MRI study	10.1111/jep.12913
M. Huang; F. Zhou; L. Wu; B. Wang; H. Wan; F. Li; X. Zeng; H. Gong	2018	Synchronization within, and interactions between, the default mode and dorsal attention networks in relapsing-remitting multiple sclerosis	10.2147/NDT.S155478
A. Harel; D. Sperling; M. Petracca; A. Ntranos; I. Katz-Sand; S. Krieger; F. Lublin; Z. Wang; Y. Liu; M. Inglese	2018	Brain microstructural injury occurs in patients with RRMS despite €no evidence of disease activity'	10.1136/jnnp-2017-317606
A. Ernst; M. Sourty; D. Roquet; V. Noblet; D. Gounot; F. Blanc; J. de Seze; L. Manning	2018	Benefits from an autobiographical memory facilitation programme in relapsing-remitting multiple sclerosis patients: a clinical and neuroimaging study	10.1080/09602011.2016.1240697
L. De Meijer; D. Merlo; O. Skibina; E. J. Grobbee; J. Gale; J. Haartsen; P. Maruff; D. Darby; H. Butzkueven; A. Van der Walt	2018	Monitoring cognitive change in multiple sclerosis using a computerized cognitive battery	10.1177/2055217318815513

S. Barone; M. E. Caligiuri; P. Valentino; A. Cherubini; C. Chiriaco; A. Granata; E. Filippelli; T. Tallarico; R. Nisticò; A. Quattrone	2018	Multimodal assessment of normal-appearing corpus callosum is a useful marker of disability in relapsing-remitting multiple sclerosis: an MRI cluster analysis study	10.1007/s00415-018-8980-y
A. Altermatt; L. Gaetano; S. Magon; D. A. Häring; D. Tomic; J. Wuerfel; E. W. Radue; L. Kappos; T. Sprenger	2018	Clinical Correlations of Brain Lesion Location in Multiple Sclerosis: Voxel-Based Analysis of a Large Clinical Trial Dataset	10.1007/s10548-018-0652-9
R. Zivadinov; J. Hagemeier; N. Bergsland; E. Tavazzi; B. Weinstock-Guttman	2018	Effect of dimethyl fumarate on gray and white matter pathology in subjects with relapsing multiple sclerosis: a longitudinal study	10.1111/ene.13562
A. Tacchino; C. Saiote; G. Brichetto; G. Bommarito; L. Roccatagliata; C. Cordano; M. A. Battaglia; G. L. Mancardi; M. Inglese	2018	Motor Imagery as a Function of Disease Severity in Multiple Sclerosis: An fMRI Study	10.3389/fnhum.2017.00628
M. O. Shatskova; G. A. Stashuk; V. Y. Lijdvoy; S. V. Kotov	2018	Cognitive dysfunction and the progression of neurodegenerative process in patients with multiple sclerosis	10.17116/jnevro201811808229
F. Schweser; A. Martins; J. Hagemeier; F. C. Lin; J. Hanspach; B. Weinstock-Guttman; S. Hametner; N. Bergsland; M. G. Dwyer; R. Zivadinov	2018	Mapping of thalamic magnetic susceptibility in multiple sclerosis indicates decreasing iron with disease duration: A proposed mechanistic relationship between inflammation and oligodendrocyte vitality	10.1016/j.neuroimage.2017.10.063
I. Rorsman; C. Petersen; P. C. Nilsson	2018	Cognitive functioning following one-year natalizumab treatment: A non-randomized clinical trial	10.1111/ane.12833
A. Raji; A. C. Ostwaldt; R. Opfer; P. Suppa; L. Spies; G. Winkler	2018	MRI-Based Brain Volumetry at a Single Time Point Complements Clinical Evaluation of Patients With Multiple Sclerosis in an Outpatient Setting	10.3389/fneur.2018.00545
K. A. Meijer; A. J. C. Eijlers; J. J. G. Geurts; M. M. Schoonheim	2018	Staging of cortical and deep grey matter functional connectivity changes in multiple sclerosis	10.1136/jnnp-2017-316329
S. J. Lin; I. Vavasour; B. Kosaka; D. K. B. Li; A. Traboulsee; A. MacKay; M. J. McKeown	2018	Education, and the balance between dynamic and stationary functional connectivity jointly support executive functions in relapsing-remitting multiple sclerosis	10.1002/hbm.24343
L. Kadrnozkova; M. Vaneckova; L. Sobisek; B. Benova; K. Kucerova; J. Motyl; M. Andelova; K. Novotna; J. L. Preiningerova; J. Krasensky; E. Havrdova; D. Horakova; T. Uher	2018	Combining clinical and magnetic resonance imaging markers enhances prediction of 12-year employment status in multiple sclerosis patients	10.1016/j.jns.2018.02.045
M. Hoseinipourasl; M. Zandkarimi; J. Abdolmohammadi; K. Sharifi; S. Miraki	2018	Evaluation the FLAIR sensitivity and DWI post-inject in comparison with delayed enhancement T1w for better detection of active MS lesions	10.31661/jbpe.v0i0.967

J. R. Evana; S. B. Bozkurta; N. C. Thomasa; F. Bagnatoa	2018	Alemtuzumab for the treatment of multiple sclerosis	10.1080/14712598.2018.1425388
E. Curti; S. Graziuso; E. Tsantes; G. Crisi; F. Granella	2018	Correlation between cortical lesions and cognitive impairment in multiple sclerosis	10.1002/brb3.955
T. Chitnis; D. L. Arnold; B. Banwell; W. Brück; A. Ghezzi; G. Giovannoni; B. Greenberg; L. Krupp; K. Rostásy; M. Tardieu; E. Waubant; J. S. Wolinsky; A. Bar-Or; T. Stites; Y. Chen; N. Putzki; M. Merschhemke; J. Gärtner	2018	Trial of fingolimod versus interferon beta-1a in pediatric multiple sclerosis	10.1056/NEJMoa1800149
A. Cancelli; C. Cottone; A. Giordani; S. Migliore; D. Lupoi; C. Porcaro; M. Mirabella; P. M. Rossini; M. M. Filippi; F. Tecchio	2018	Personalized, bilateral whole-body somatosensory cortex stimulation to relieve fatigue in multiple sclerosis	10.1177/1352458517720528
M. Cambron; T. Reynders; J. Debruyne; H. Reyngoudt; A. Ribbens; E. Achten; G. Laureys	2018	Targeting phosphocreatine metabolism in relapsing–remitting multiple sclerosis: evaluation with brain MRI, 1H and 31P MRS, and clinical and cognitive testing	10.1007/s00415-018-9039-9
L. J. M. Blikman; J. Van Meeteren; D. Rizopoulos; V. De Groot; H. Beckerman; H. J. Stam; J. B. J. Bussmann; A. Malekzadeh; L. E. Van Den Akker; M. Looijmans; S. A. Sanches; J. Dekker; E. H. Collette; B. W. Van Oosten; C. E. Teunissen; M. A. Blankenstein; I. C. J. M. Eijssen; M. Rietberg; M. Heine; O. Verschuren; G. Kwakkel; J. M. A. Visser-Meily; I. G. L. Van De Port; E. Lindeman; L. J. M. Blikman; J. Van Meeteren; J. B. J. Bussmann; H. J. Stam; R. Q. Hintzen; H. G. A. Hacking; E. L. Hoogervorst; S. T. F. M. Frequin; H. Knoop; B. A. De Jong; G. Bleijenberg; F. A. J. De Laat; M. C. Verhulsdonck; E. Van Munster; C. J. Oosterwijk; G. J. Aarts; T.-A. s. group	2018	Physical behaviour is weakly associated with physical fatigue in persons with multiple sclerosis-related fatigue	10.2340/16501977-2375
A. Bisecco; F. D. Nardo; R. Docimo; G. Caiazzo; A. d'Ambrosio; S. Bonavita; R. Capuano; L. Sinisi; M. Cirillo; F. Esposito; G. Tedeschi; A. Gallo	2018	Fatigue in multiple sclerosis: The contribution of resting-state functional connectivity reorganization	10.1177/1352458517730932

A. Baumgartner; L. Frings; F. Schiller; O. Stich; M. Mix; K. Egger; G. Schluh; S. Rauer; P. T. Meyer	2018	Regional neuronal activity in patients with relapsing remitting multiple sclerosis	10.1111/ane.13012
L. Baquet; H. Hasselmann; S. Patra; J. P. Stellmann; E. Vettorazzi; A. K. Engel; S. C. Rosenkranz; J. Poettgen; S. M. Gold; K. H. Schulz; C. Heesen	2018	Short-term interval aerobic exercise training does not improve memory functioning in relapsing-remitting multiple sclerosis— A randomized controlled trial	10.7717/peerj.6037
A. K. Artemiadis; M. C. Anagnostouli; I. G. Zalonis; K. G. Chairopoulos; N. I. Triantafyllou	2018	Structural MRI correlates of cognitive event-related potentials in multiple sclerosis	10.1097/WNP.0000000000000473
A. Artemiadis; M. Anagnostouli; I. Zalonis; K. Chairopoulos; N. Triantafyllou	2018	Structural MRI correlates of cognitive function in multiple sclerosis	10.1016/j.msard.2018.02.003
K. Yarraguntla; F. Bao; S. Lichtman-Mikol; S. Razmjou; C. Santiago-Martinez; N. Seraji-Bozorgzad; S. Sriwastava; E. Bernitsas	2019	Characterizing fatigue-related white matter changes in ms: A proton magnetic resonance spectroscopy study	10.3390/brainsci9050122
S. Jaeger; F. Paul; M. Scheel; A. Brandt; J. Heine; D. Pach; C. M. Witt; J. Bellmann-Strobl; C. Finke	2019	Multiple sclerosis-related fatigue: Altered resting-state functional connectivity of the ventral striatum and dorsolateral prefrontal cortex	10.1177/1352458518758911
D. Iancheva; A. Trenova; S. Mantarova; K. Terziyski	2019	Functional magnetic resonance imaging correlations between fatigue and cognitive performance in patients with relapsing remitting multiple sclerosis	10.3389/fpsyt.2019.00754
T. A. Hassan; S. F. Elkholy; B. E. Mahmoud; M. ElSherbiny	2019	Multiple sclerosis and depressive manifestations: can diffusion tensor MR imaging help in the detection of microstructural white matter changes?	10.1186/s43055-019-0033-8
I. Håkansson; L. Johansson; C. Dahle; M. Vrethem; J. Ernerudh	2019	Fatigue scores correlate with other self-assessment data, but not with clinical and biomarker parameters, in CIS and RRMS	10.1016/j.msard.2019.101424
S. Vukusic; D. Brassat; J. de Seze; G. Izquierdo; A. Lysandropoulos; W. Moll; L. Vanopdenbosch; M. J. Arque; M. Kertous; P. Rufi; C. Oreja-Guevara	2019	Single-arm study to assess comprehensive infusion guidance for the prevention and management of the infusion associated reactions (IARs) in relapsing-remitting multiple sclerosis (RRMS) patients treated with alemtuzumab (EMERALD)	10.1016/j.msard.2019.01.019
P. Stefancin; S. T. Govindarajan; L. Krupp; L. Charvet; T. Q. Duong	2019	Resting-state functional connectivity networks associated with fatigue in multiple sclerosis with early age onset	10.1016/j.msard.2019.03.020
L. N. Prakhova; A. G. Ilves; Z. I. Savintseva; N. M. Kuznetsova; K. S. Rubanik; G. V. Kataeva	2019	Regional cerebral hypoperfusion as a cause of symptoms and progression of multiple sclerosis	10.20538/1682-0363-2019-4-102-109

L. Pfaff; J. Lamy; V. Noblet; D. Gounot; J. B. Chanson; J. de Seze; F. Blanc	2019	Emotional disturbances in multiple sclerosis: A neuropsychological and fMRI study	10.1016/j.cortex.2019.02.017
L. Patrucco; E. Cristiano; F. Sánchez; J. Miguez; J. I. Rojas	2019	Real-World Effectiveness and Safety of Fingolimod in Patients with Relapsing Remitting Multiple Sclerosis: A Prospective Analysis in Buenos Aires, Argentina	10.1097/WNF.0000000000000358
I. M. Nauta; L. J. Balk; J. M. Sonder; H. E. Hulst; B. M. J. Uitdehaag; L. Fasotti; B. A. de Jong	2019	The clinical value of the patient-reported multiple sclerosis neuropsychological screening questionnaire	10.1177/1352458518777295
M. Mäurer; K. Tiel-Wilck; E. Oehm; N. Richter; M. Springer; P. Oschmann; A. Manzel; S. Hieke-Schulz; V. Zingler; J. A. Kandenwein; T. Ziemssen; R. A. Linker	2019	Reasons to switch: a noninterventional study evaluating immunotherapy switches in a large German multicentre cohort of patients with relapsing-remitting multiple sclerosis	10.1177/1756286419892077
M. A. Kotb; A. M. Kama; N. M. Aldossary; M. A. Bedewi	2019	Effect of vitamin D replacement on depression in multiple sclerosis patients	10.1016/j.msard.2019.01.029
G. K. Chaseling; D. R. Allen; S. Vucic; M. Barnett; E. Frohman; S. L. Davis; O. Jay	2019	Core temperature is not elevated at rest in people with relapsing-remitting multiple sclerosis	10.1016/j.msard.2019.01.013
A. Carotenuto; T. Costabile; M. Moccia; F. Falco; M. R. Scalà; C. V. Russo; F. Sacca; A. De Rosa; R. Lanzillo; V. B. Morra	2019	Olfactory function and cognition in relapsing-remitting and secondary-progressive multiple sclerosis	10.1016/j.msard.2018.09.024
R. W. Browne; D. Jakimovski; N. Ziliotto; J. Kuhle; F. Bernardi; B. Weinstock-Guttman; R. Zivadinov; M. Ramanathan	2019	High-density lipoprotein cholesterol is associated with multiple sclerosis fatigue: A fatigue-metabolism nexus?	10.1016/j.jacl.2019.06.003
J. N. Brenton; B. Banwell; A. G. Christina Bergqvist; D. Lehner-Gulotta; L. Gampper; E. Leytham; R. Coleman; M. D. Goldman	2019	Pilot study of a ketogenic diet in relapsing-remitting MS	10.1212/NXI.0000000000000565

H. E. M. Braakhuis; M. A. M. Berger; G. A. Van Der Stok; J. Van Meeteren; V. De Groot; H. Beckerman; J. B. J. Bussmann; A. Malekzadeh; L. E. Van Den Akker; M. Looijmans; S. A. Sanches; J. Dekker; E. H. Collette; B. W. Van Oosten; C. E. Teunissen; M. A. Blankenstein; I. C. J. M. Eijssen; M. Rietberg; M. Heine; O. Verschuren; G. Kwakkel; J. M. A. Visser-Meily; I. G. L. Van De Port; E. Lindeman; L. J. M. Blikman; J. Van Meeteren; J. B. J. Bussmann; H. J. Stam; R. Q. Hintzen; H. G. A. Hacking; E. L. Hoogervorst; S. T. F. M. Frequin; H. Knoop; B. A. De Jong; G. Bleijenberg; F. A. J. De Laat; M. C. Verhulsdonck; E. T. L. Van Munster; C. J. Oosterwijk; G. J. Aarts	2019	Three distinct physical behavior types in fatigued patients with multiple sclerosis	10.1186/s12984-019-0573-1
A. Bisecco; M. Altieri; G. Santangelo; F. Di Nardo; R. Docimo; G. Caiazzo; R. Capuano; S. Pappacena; A. d'Ambrosio; S. Bonavita; F. Trojsi; M. Cirillo; F. Esposito; G. Tedeschi; A. Gallo	2019	Resting-State Functional Correlates of Social Cognition in Multiple Sclerosis: An Explorative Study	10.3389/fnbeh.2019.00276
A. K. Andreasen; P. Iversen; L. Marstrand; V. Siersma; H. R. Siebner; F. Sellebjerg	2019	Structural and cognitive correlates of fatigue in progressive multiple sclerosis	10.1080/01616412.2018.1547813
V. Martinovic; I. Nikolic; S. Mesaros; J. Drulovic	2020	Bilateral horizontal gaze palsy in benign multiple sclerosis	10.1007/s13760-018-01064-5
L. Wu; M. Huang; F. Zhou; X. Zeng; H. Gong	2020	Distributed causality in resting-state network connectivity in the acute and remitting phases of RRMS	10.1186/s12868-020-00590-4
A. C. Vogel; H. Schmidt; S. Loud; R. McBurney; F. J. Mateen	2020	Impact of the COVID-19 pandemic on the health care of >1,000 People living with multiple sclerosis: A cross-sectional study	10.1016/j.msard.2020.102512
A. Salter; K. Kowalec; K. C. Fitzgerald; G. Cutter; R. A. Marrie	2020	Comorbidity is associated with disease activity in MS: Findings from the CombiRx trial	10.1212/WNL.0000000000010024
K. Okada; S. Kakeda; M. Tahara	2020	Olfactory identification associates with cognitive function and the third ventricle width in patients with relapsing-remitting multiple sclerosis	10.1016/j.msard.2019.101507
A. Lazzarotto; M. Margoni; S. Franciotta; S. Zywicki; A. Riccardi; D. Poggiali; M. Anglani; P. Gallo	2020	Selective Cerebellar Atrophy Associates with Depression and Fatigue in the Early Phases of Relapse-Onset Multiple Sclerosis	10.1007/s12311-019-01096-4

H. Joly; N. Capet; L. Mondot; M. Cohen; C. Suply; S. Bresch; C. Lebrun-Frenay	2020	Thalamic atrophy correlates with dysfunctional impulsivity in multiple sclerosis	10.1016/j.msard.2020.102374
S. Golde; J. Heine; J. Pöttgen; M. Mantwill; S. Lau; K. Wingenfeld; C. Otte; I. K. Penner; A. K. Engel; C. Heesen; J. P. Stellmann; I. Dziobek; C. Finke; S. M. Gold	2020	Distinct Functional Connectivity Signatures of Impaired Social Cognition in Multiple Sclerosis	10.3389/fneur.2020.00507
A. Damasceno; L. R. Pimentel-Silva; B. P. Damasceno; F. Cendes	2020	Cognitive trajectories in relapsing–remitting multiple sclerosis: A longitudinal 6-year study	10.1177/1352458519878685
A. Conte; C. Giannì; D. Belvisi; A. Cortese; N. Petsas; M. Tartaglia; P. Cimino; E. Millefiorini; A. Berardelli; P. Pantano	2020	Deep grey matter involvement and altered sensory gating in multiple sclerosis	10.1177/1352458519845287
S. Collorone; N. Cawley; F. Grussu; F. Prados; F. Tona; A. Calvi; B. Kanber; T. Schneider; L. Kipp; H. Zhang; D. C. Alexander; A. J. Thompson; A. Toosy; C. A. M. G. Wheeler-Kingshott; O. Ciccarelli	2020	Reduced neurite density in the brain and cervical spinal cord in relapsing–remitting multiple sclerosis: A NODDI study	10.1177/1352458519885107
A. Carotenuto; H. Wilson; B. Giordano; S. P. Caminiti; Z. Chappell; S. C. R. Williams; A. Hammers; E. Silber; P. Brex; M. Politis	2020	Impaired connectivity within neuromodulatory networks in multiple sclerosis and clinical implications	10.1007/s00415-020-09806-3
C. Bauer; T. B. Dyrby; F. Sellebjerg; K. S. Madsen; O. Svolgaard; M. Blinkenberg; H. R. Siebner; K. W. Andersen	2020	Motor fatigue is associated with asymmetric connectivity properties of the corticospinal tract in multiple sclerosis	10.1016/j.nicl.2020.102393
J. R. Abbatemarco; D. Ontaneda; K. Nakamura; S. Husak; Z. Wang; E. Alshehri; R. A. Bermel; D. S. Conway	2020	Comorbidity effect on processing speed test and MRI measures in multiple sclerosis patients	10.1016/j.msard.2020.102593
A. Andravizou; V. Siokas; A. Artemiadis; C. Bakirtzis; A. M. Aloizou; N. Grigoriadis; M. H. Kosmidis; G. Nasios; L. Messinis; G. Hadjigeorgiou; E. Dardiotis; E. Peristeri	2020	Clinically reliable cognitive decline in relapsing remitting multiple sclerosis: Is it the tip of the iceberg?	10.1080/01616412.2020.1761175
P. Zuber; C. Tsagkas; A. Papadopoulou; L. Gaetano; M. Huerbin; E. Geiter; A. Altermatt; K. Parmar; T. Ettlin; C. Schuster-Amft; Z. Suica; H. Alrasheed; J. Wuerfel; J. Kesselring; L. Kappos; T. Sprenger; S. Magon	2020	Efficacy of inpatient personalized multidisciplinary rehabilitation in multiple sclerosis: behavioural and functional imaging results	10.1007/s00415-020-09768-6

L. Zhao; A. Ng; Q. Chen; B. Lam; J. Abrigo; C. Au; V. C. T. Mok; A. Wong; A. Y. Lau	2020	Impaired cognition is related to microstructural integrity in relapsing remitting multiple sclerosis	10.1002/acn3.51100
A. van Wijnen; F. Petrov; M. Maiworm; S. Frisch; C. Foerch; E. Hattingen; H. Steinmetz; J. C. Klein; R. Deichmann; M. Wagner; R. M. Gracien	2020	Cortical quantitative MRI parameters are related to the cognitive status in patients with relapsing-remitting multiple sclerosis	10.1007/s00330-019-06437-9
D. Valdés Cabrera; R. Stobbe; P. Smyth; F. Giuliani; D. Emery; C. Beaulieu	2020	Diffusion tensor imaging tractography reveals altered fornix in all diagnostic subtypes of multiple sclerosis	10.1002/brb3.1514
T. Singhal; S. Cicero; H. Pan; K. Carter; S. Dubey; R. Chu; B. Glanz; S. Hurwitz; S. Tauhid; M. A. Park; M. Kijewski; E. Stern; R. Bakshi; D. Silbersweig; H. L. Weiner	2020	Regional microglial activation in the substantia nigra is linked with fatigue in MS	10.1212/nxi.0000000000000854
E. Schmidt; C. Schinke; M. Rullmann; J. Luthardt; G. A. Becker; S. Haars; M. Stoppe; D. Lobsien; K. T. Hoffmann; O. Sabri; S. Hesse; F. Then Bergh	2020	Changes of central noradrenaline transporter availability in immunotherapy-naïve multiple sclerosis patients	10.1038/s41598-020-70732-5
R. Sacco; S. Emming; C. Gobbi; C. Zecca; S. Monticelli	2020	Rebound of disease activity after fingolimod withdrawal: Immunological and gene expression profiling	10.1016/j.msard.2020.101927
E. Ruiu; R. Dubbioso; K. H. Madsen; O. Svolgaard; E. Raffin; K. W. Andersen; A. N. Karabano; H. R. Siebner	2020	Probing Context-Dependent Modulations of Ipsilateral Premotor-Motor Connectivity in Relapsing-Remitting Multiple Sclerosis	10.3389/fneur.2020.00193
L. Prosperini; A. Cortese; M. Lucchini; L. Boffa; G. Borriello; M. C. Buscarinu; F. Capone; D. Centonze; C. De Fino; D. De Pascalis; R. Fantozzi; E. Ferraro; M. Filippi; S. Galgani; C. Gasperini; S. Haggiag; D. Landi; G. Marfia; G. Mataluni; E. Millefiorini; M. Mirabella; F. Monteleone; V. Nociti; S. Pontecorvo; S. Romano; S. Ruggieri; M. Salvetti; C. Tortorella; S. Zannino; G. Di Battista	2020	Exit strategies for “needle fatigue” in multiple sclerosis: a propensity score-matched comparison study	10.1007/s00415-019-09625-1
O. Mirmosayyeb; S. Brand; M. Barzegar; A. Afshari-Safavi; N. Nehzat; V. Shaygannejad; D. S. Bahmani	2020	Clinical characteristics and disability progression of early-and late-onset multiple sclerosis compared to adult-onset multiple sclerosis	10.3390/jcm9051326
R. Meli; L. Roccatagliata; E. Capello; N. Bruschi; A. Uccelli; G. Mancardi; M. Inglese; M. Pardini	2020	Ecological impact of isolated cognitive relapses in MS	10.1177/1352458518813722
V. C. Mastorodemos; S. G. Ioannidis; E. Z. Papadaki; P. D. Mitsias	2020	Posterior Reversible Encephalopathy Syndrome, Multiple Sclerosis and interferon therapy: Association, co-incidence or convoluted interplay?	10.1016/j.msard.2020.102356

O. Marchesi; C. Vizzino; A. Meani; L. Conti; G. C. Riccitelli; P. Preziosa; M. Filippi; M. A. Rocca	2020	Fatigue in multiple sclerosis patients with different clinical phenotypes: a clinical and magnetic resonance imaging study	10.1111/ene.14471
A. Malekzadeh; I. Bader; J. van Dieteren; A. C. Heijboer; H. Beckerman; J. W. R. Twisk; V. de Groot; C. E. Teunissen; T.-A. S. Group	2020	Diurnal Cortisol Secretion Is Not Related to Multiple Sclerosis-Related Fatigue	10.3389/fneur.2019.01363
N. C. Landmeyer; I. Dzionsko; L. Brockhoff; H. Wiendl; G. Domes; J. Bölte; J. Krämer; S. G. Meuth; A. Johnen	2020	The Agony of Choice? Preserved Affective Decision Making in Early Multiple Sclerosis	10.3389/fneur.2020.00914
T. P. Labbe; M. Zurita; C. Montalba; E. L. Ciampi; J. P. Cruz; M. Vasquez; S. Uribe; N. Crossley; C. Cárcamo	2020	Social cognition in Multiple Sclerosis is associated to changes in brain connectivity: A resting-state fMRI study	10.1016/j.msard.2020.102333
L. Jeung; L. M. G. Smits; E. L. J. Hoogervorst; B. W. van Oosten; S. T. F. M. Frequin	2020	A tumefactive demyelinating lesion in a person with MS after five years of fingolimod	10.1016/j.msard.2020.101978
M. Houniet-De Gier; H. Beckerman; K. Van Vliet; H. Knoop; V. De Groot	2020	Testing non-inferiority of blended versus face-to-face cognitive behavioural therapy for severe fatigue in patients with multiple sclerosis and the effectiveness of blended booster sessions aimed at improving long-term outcome following both therapies: Study protocol for two observer-blinded randomized clinical trials	10.1186/s13063-019-3825-2
G. Gonzalez-Escamilla; D. Ciolac; S. De Santis; A. Radetz; V. Fleischer; A. Droby; A. Roebroeck; S. G. Meuth; M. Muthuraman; S. Groppa	2020	Gray matter network reorganization in multiple sclerosis from 7-Tesla and 3-Tesla MRI data	10.1002/acn3.51029
B. Goitia; D. Bruno; S. Abrevaya; L. Sedeño; A. Ibáñez; F. Manes; M. Sigman; V. Sinay; T. Torralva; J. Duncan; M. Roca	2020	The relationship between executive functions and fluid intelligence in multiple sclerosis	10.1371/journal.pone.0231868
N. E. Fritz; E. M. Edwards; J. Keller; A. Eloyan; P. A. Calabresi; K. M. Zackowski	2020	Combining magnetization transfer ratio mri and quantitative measures of walking improves the identification of fallers in MS	10.3390/brainsci10110822
O. Fernández; G. Izquierdo; E. Aguera; C. Ramo; M. Hernandez; D. Silva; R. Walker; H. Butzkueven; C. Wang; M. Barnett	2020	Comparison of first-line and second-line use of fingolimod in relapsing MS: The open-label EARLIMS study	10.1177/2055217320957358

S. Engel; C. Graetz; A. Salmen; M. Muthuraman; G. Toenges; B. Ambrosius; A. Bayas; A. Berthele; C. Heesen; L. Klotz; T. Kümpfel; R. A. Linker; S. G. Meuth; F. Paul; M. Stangel; B. Tackenberg; F. Then Bergh; H. Tumani; F. Weber; B. Wildemann; U. K. Zettl; G. Antony; S. Bittner; S. Groppa; B. Hemmer; H. Wiendl; R. Gold; F. Zipp; C. M. Lill; F. Luessi	2020	Is APOE ε4 associated with cognitive performance in early MS?	10.1212/nxi.0000000000000728
A. Damasceno; L. R. Pimentel-Silva; B. P. Damasceno; F. Cendes	2020	Exploring the performance of outcome measures in MS for predicting cognitive and clinical progression in the following years	10.1016/j.msard.2020.102513
L. Couloume; L. Barbin; E. Leray; S. Wiertlewski; E. Le Page; A. Kerbrat; S. Ory; D. Le Port; G. Edan; D. A. Laplaud; L. Michel	2020	High-dose biotin in progressive multiple sclerosis: A prospective study of 178 patients in routine clinical practice	10.1177/1352458519894713
D. S. Conway; C. M. Hersh; H. C. Harris; L. Hua	2020	Duration of natalizumab therapy and reasons for discontinuation in a multiple sclerosis population	10.1177/2055217320902488
G. Comi; M. S. Freedman; J. E. Meca-Lallana; P. Vermersch; B. J. Kim; A. Parajeles; K. R. Edwards; R. Gold; H. Korideck; J. Chavin; E. M. Poole; P. K. Coyle	2020	Prior treatment status: Impact on the efficacy and safety of teriflunomide in multiple sclerosis	10.1186/s12883-020-01937-4
H. Butzkueven; S. Licata; D. Jeffery; D. L. Arnold; M. Filippi; J. J. G. Geurts; S. Santra; N. Campbell; P. R. Ho	2020	Natalizumab versus fingolimod for patients with active relapsing-remitting multiple sclerosis: results from REVEAL, a prospective, randomised head-to-head study	10.1136/bmjopen-2020-038861
H. Beckerman; I. C. Eijssen; J. van Meeteren; M. C. Verhulsdonck; V. de Groot	2020	Fatigue Profiles in Patients with Multiple Sclerosis are Based on Severity of Fatigue and not on Dimensions of Fatigue	10.1038/s41598-020-61076-1
F. Barrero; J. Mallada-Frechin; M. L. Martínez-Ginés; M. E. Marzo; V. Meca-Lallana; G. Izquierdo; J. R. Ara; C. Oreja-Guevara; J. Meca-Lallana; L. Forero; I. Sánchez-Vera; M. J. Moreno	2020	Spanish real-world experience with fingolimod in relapsing-remitting multiple sclerosis patients: MS NEXT study	10.1371/journal.pone.0230846
R. Arroyo; D. P. Bury; J. D. Guo; D. H. Margolin; M. Melanson; N. Daizadeh; D. Cella	2020	Impact of alemtuzumab on health-related quality of life over 6 years in CARE-MS II trial extension patients with relapsing-remitting multiple sclerosis	10.1177/1352458519849796
H. Algahtani; B. Shirah; Y. Al Malik; I. Meftah	2020	Fingolimod for Relapsing-Remitting Multiple Sclerosis: The Experience from Saudi Arabia	10.1097/WNF.0000000000000378

M. Barešić; M. Reihl Crnogaj; I. Zadro; B. Anić	2021	Demyelinating disease (multiple sclerosis) in a patient with psoriatic arthritis treated with adalimumab: a case-based review	10.1007/s00296-021-04995-0
A. Zanghì; E. D'Amico; S. Lo Fermo; F. Patti	2021	Exploring polypharmacy phenomenon in newly diagnosed relapsing–remitting multiple sclerosis: a cohort ambispective single-centre study	10.1177/2040622320983121
Y. Yalachkov; V. Anschuetz; J. Jakob; M. A. Schaller-Paule; J. H. Schaefer; A. Reilaender; L. Friedauer; M. Behrens; C. Foerch	2021	C-Reactive Protein Levels and Gadolinium-Enhancing Lesions Are Associated With the Degree of Depressive Symptoms in Newly Diagnosed Multiple Sclerosis	10.3389/fneur.2021.719088
A. Trufanov; G. Bisaga; D. Skulyabin; A. Temniy; M. Poplyak; O. Chakchir; A. Efimtsev; T. Dmitriy; M. Odinak; I. Litvinenko	2021	Thalamic nuclei degeneration in multiple sclerosis	10.1016/j.jocn.2021.05.043
F. B. Tijhuis; T. A. A. Broeders; F. A. N. Santos; M. M. Schoonheim; J. Killestein; C. E. Leurs; Q. van Geest; M. D. Steenwijk; J. J. G. Geurts; H. E. Hulst; L. Douw	2021	Dynamic functional connectivity as a neural correlate of fatigue in multiple sclerosis	10.1016/j.nicl.2020.102556
F. Stascheit; L. Li; K. Mai; K. Baum; E. Siebert; K. Ruprecht	2021	Delayed onset hypophysitis after therapy with daclizumab for multiple sclerosis – A report of two cases	10.1016/j.jneuroim.2020.577469
J. M. Soares; R. Conde; R. Magalhaes; P. Marques; R. Magalhaes; L. Gomes; O. F. Gonçalves; M. Arantes; A. Sampaio	2021	Alterations in functional connectivity are associated with white matter lesions and information processing efficiency in multiple sclerosis	10.1007/s11682-020-00264-z
G. Santangelo; M. D. Corte; M. Sparaco; G. Miele; F. Garramone; M. Cropano; S. Esposito; L. Lavorgna; A. Gallo; G. Tedeschi; S. Bonavita	2021	Coping strategies in relapsing-remitting multiple sclerosis non-depressed patients and their associations with disease activity	10.1007/s13760-019-01212-5
A. Saberi; A. Abdolalizadeh; E. Mohammadi; M. A. Nahayati; H. Bagheri; B. Shekarchi; J. Kargar	2021	Thalamic shape abnormalities in patients with multiple sclerosis-related fatigue	10.1097/wnr.0000000000001616
J. I. Rojas; L. Patrucco; R. Alonso; O. Garcea; N. Deri; E. Carnero Contentti; P. A. Lopez; J. P. Pettinicchi; A. Caride; E. Cristiano	2021	Diagnostic uncertainty during the transition to secondary progressive multiple sclerosis: Multicenter study in Argentina	10.1177/1352458520924586
M. A. Rocca; P. Valsasina; B. Colombo; V. Martinelli; M. Filippi	2021	Cortico-subcortical functional connectivity modifications in fatigued multiple sclerosis patients treated with fampridine and amantadine	10.1111/ene.14867

M. Palotai; M. Wallack; G. Kujbus; A. Dalnoki; C. Guttmann	2021	Usability of a mobile app for real-time assessment of fatigue and related symptoms in patients with multiple sclerosis: Observational study	10.2196/19564
S. Ooi; T. Kalincik; P. Perucca; M. Monif	2021	The prevalence of epileptic seizures in multiple sclerosis in a large tertiary hospital in Australia	10.1177/2055217321989767
K. Ogisu; M. Niino; Y. Miyazaki; S. Kikuchi	2021	Optimal indicator for histogram analysis of fractional anisotropy for normal-appearing white matter in multiple sclerosis	10.54029/2021PNK
P. Newland; L. Chen; P. Sun; J. Zempel	2021	Neurophysiological Correlates of Fatigue in Multiple Sclerosis	10.1016/j.nurpra.2021.02.012
R. Manca; M. Mitolo; I. Wilkinson; D. Paling; B. Sharrack; A. Venneri	2021	A network-based cognitive training induces cognitive improvements and neuroplastic changes in patients with relapsing-remitting multiple sclerosis: An exploratory case-control study	10.4103/1673-5374.300450
T. P. Labbe; C. Montalba; M. Zurita; E. L. Ciampi; J. P. Cruz; M. Vasquez; S. Uribe; N. Crossley; C. Cárcamo	2021	Regional brain atrophy is related to social cognition impairment in multiple sclerosis	10.1590/0004-282X-ANP-2020-0162
O. O. Kopchak; T. A. Odintsova; O. R. Pulyk	2021	COGNITIVE FUNCTIONS IN MULTIPLE SCLEROSIS PATIENTS DEPENDING ON THE DIFFERENT RISK FACTORS PRESENCE	10.36740/wlek202110115
O. O. Kopchak; T. A. Odintsova	2021	Cognitive impairment and depression in patients with relapsing-remitting multiple sclerosis depending on age and neuroimaging findings	10.1186/s41983-021-00376-3
E. M. Khedr; N. Abo-Elfetoh; E. Deaf; H. M. Hassan; M. T. Amin; R. K. Soliman; A. A. Attia; A. A. Zarzour; M. Zain; A. Mohamed-Hussein; M. K. Hashem; S. M. Hassany; A. Aly; A. Shoyb; M. Saber	2021	Surveillance study of acute neurological manifestations among 439 egyptian patients with COVID-19 in assiut and Aswan University Hospitals	10.1159/000513647
B. C. Healy; B. I. Glanz; E. Swallow; J. Signorovitch; K. Hagan; D. Silva; C. Pelletier; T. Chitnis; H. Weiner	2021	Confirmed disability progression provides limited predictive information regarding future disease progression in multiple sclerosis	10.1177/2055217321999070
J. Deverdun; A. Coget; X. Ayrignac; C. Carra-Dalliere; A. Krainik; A. Metzger; P. Labauge; N. Menjot de Champfleur; E. Le Bars	2021	Cerebral Vasoreactivity as an Indirect MRI Marker of White Matter Tracts Alterations in Multiple Sclerosis	10.1007/s10548-021-00819-3
T. Carandini; M. Mancini; I. Bogdan; C. L. Rae; A. W. Barritt; M. Clerico; A. Sethi; N. Harrison; W. Rashid; E. Scarpini; D. Galimberti; M. Bozzali; M. Cercignani	2021	In vivo evidence of functional disconnection between brainstem monoaminergic nuclei and brain networks in multiple sclerosis	10.1016/j.msard.2021.103224

R. H. B. Benedict; J. Pol; F. Yasin; D. Hojnacki; C. Kolb; S. Eckert; B. Tacca; A. Drake; C. Wojcik; S. A. Morrow; D. Jakimovski; T. A. Fuchs; M. G. Dwyer; R. Zivadinov; B. Weinstock-Guttman	2021	Recovery of cognitive function after relapse in multiple sclerosis	10.1177/1352458519898108
A. M. Beaudoin; F. Rheault; G. Theaud; F. Laberge; K. Whittingstall; A. Lamontagne; M. Descoteaux	2021	Modern Technology in Multi-Shell Diffusion MRI Reveals Diffuse White Matter Changes in Young Adults With Relapsing-Remitting Multiple Sclerosis	10.3389/fnins.2021.665017
J. Arm; O. Al-Iedani; K. Ribbons; R. Lea; J. Lechner-Scott; S. Ramadan	2021	Biochemical Correlations with Fatigue in Multiple Sclerosis Detected by MR 2D Localized Correlated Spectroscopy	10.1111/jon.12836
S. Ziccardi; M. Pitteri; H. M. Genova; M. Calabrese	2021	Social Cognition in Multiple Sclerosis: A 3-Year Follow-Up MRI and Behavioral Study	10.3390/diagnostics11030484
X. S. Zhao; T. Yang; F. Cheng; S. Yang; W. L. Zhu; S. W. Li; Y. P. Fan	2021	Abnormalcortical thickness in relapsing-remitting multiple sclerosis, correlations with cognition impairment, and effect of modified Bushenyisui decoction(SIC) on cognitive function of multiple sclerosis	10.19852/j.cnki.jtcm.20200730.001
C. Vinciguerra; A. Giorgio; J. Zhang; V. Nardone; R. T. Brocci; L. Pastò; C. Niccolai; M. L. Stromillo; M. Mortilla; M. P. Amato; N. De Stefano	2021	Peak width of skeletonized mean diffusivity (PSMD) and cognitive functions in relapsing-remitting multiple sclerosis	10.1007/s11682-020-00394-4
S. Shahrampour; J. Heholt; A. Wang; F. Vedaei; F. B. Mohamed; M. Alizadeh; Z. Wang; G. Zabrecky; N. Wintering; A. J. Bazzan; T. P. Leist; D. A. Monti; A. B. Newberg	2021	N-acetyl cysteine administration affects cerebral blood flow as measured by arterial spin labeling MRI in patients with multiple sclerosis	10.1016/j.heliyon.2021.e07615
K. W. Selmaj; J. A. Cohen; G. Comi; A. Bar-Or; D. L. Arnold; L. Steinman; H. P. Hartung; X. Montalban; E. K. Havrdova; B. A. C. Cree; N. Minton; J. K. Sheffield; N. Ding; L. Kappos	2021	Ozanimod in relapsing multiple sclerosis: Pooled safety results from the clinical development program	10.1016/j.msard.2021.102844
R. C. Sánchez; A. D. de la Fe; A. P. Suarez; D. Grass; T. M. Vega; A. S. Canal; D. Siniscalco; M. de los Angeles Robinson Agramonte	2021	Interferon beta 1a (Rebif®) in relapsing remitting multiple sclerosis	10.1002/ddr.21798
N. Razazian; P. Ahmadi; M. Rezaei; N. Fakhri	2021	One-year effectiveness and side effects of fingolimod in multiple sclerosis patients	

C. D. Mayo; L. Harrison; K. Attwell-Pope; L. Stuart-Hill; J. R. Gawryluk	2021	A pilot study of the impact of an exercise intervention on brain structure, cognition, and psychosocial symptoms in individuals with relapsing-remitting multiple sclerosis	10.1186/s40814-021-00806-2
E. Lozano-Soto; A. J. Cruz-Gómez; R. Gutiérrez; M. González; F. Sanmartino; R. Rashid-Lopez; R. Espinosa-Rosso; L. Forero; J. J. González-Rosa	2021	Predicting Neuropsychological Impairment in Relapsing Remitting Multiple Sclerosis: The Role of Clinical Measures, Treatment, and Neuropsychiatry Symptoms	10.1093/arclin/acaa088
J. Kaplan; T. Miller; M. Baker; B. Due; E. Zhao	2021	Repository corticotropin injection improves quality metrics in an observational study of multiple sclerosis relapse	10.2217/nmt-2021-0030
Y. He; Y. Zhu; F. Zhou	2021	Cerebellar-cerebral resting-state functional connectivity alterations in relapsing-remitting multiple sclerosis	10.3760/cma.j.cn112149-20201102-01198
T. A. Hassan; S. F. Elkholy; H. S. Shehata; N. M. Shalaby; A. N. Elmazny; M. N. Sadek; B. E. Mahmoud; M. M. Elsherbiny	2021	Fractional anisotropy measurements of the left dorsolateral prefrontal cortex for therapeutic response assessment after repetitive transcranial magnetic stimulation (rTMS) in relapsing remitting multiple sclerosis patients suffering from depression	10.1186/s43055-020-00404-x
R. Capra; V. B. Morra; M. Mirabella; C. Gasperini; C. Scandellari; R. Totaro; N. De Rossi; S. Masera; V. Zipoli; F. Patti	2021	Natalizumab is associated with early improvement of working ability in relapsing-remitting multiple sclerosis patients: WANT observational study results	10.1007/s10072-020-04838-z
J. Arm; G. Oeltzschner; O. Al-iedani; R. Lea; J. Lechner-Scott; S. Ramadan	2021	Altered in vivo brain GABA and glutamate levels are associated with multiple sclerosis central fatigue	10.1016/j.ejrad.2021.109610
T. Akdag; A. U. Uca; M. Altas; F. O. Odabas; F. Aktas	2021	Level of kisspeptin-10 in patients with multiple sclerosis and the association between third ventricle diameter size and vitamin D level	10.1556/2060.2021.00179
M. Waliszewska-Prosół; M. Nowakowska-Kotas; B. Misiak; J. Chojdak-Łukasiewicz; S. Budrewicz; A. Pokryszko-Dragan	2022	Allostatic load index in patients with multiple sclerosis: A case-control study	10.1016/j.psyneuen.2022.105788
O. Svolgaard; K. Winther Andersen; C. Bauer; K. Hougaard Madsen; M. Blinkenberg; F. Sellebjerg; H. Roman Siebner	2022	Mapping grip-force related brain activity after a fatiguing motor task in multiple sclerosis	10.1016/j.nicl.2022.103147
E. Saruhan; M. Korkmaz; B. Altiparmak; K. Tosun; G. Kutlu	2022	COMPARISON OF OREXIN-A AND NEUROFILAMENT LIGHT CHAIN LEVELS IN PATIENTS WITH RELAPSING-REMITTING MULTIPLE SCLEROSIS: A PILOT STUDY	10.18071/isz.75.0223
A. L. Ruiz-Rizzo; P. Bublak; S. Kluckow; K. Finke; C. Gaser; M. Schwab; D. Güllmar; H. J. Müller; O. Witte; S. Rupprecht	2022	Neural distinctiveness of fatigue and low sleep quality in multiple sclerosis	10.1111/ene.15445

A. Romanello; S. Krohn; N. von Schwanenflug; C. Chien; J. Bellmann-Strobl; K. Ruprecht; F. Paul; C. Finke	2022	Functional connectivity dynamics reflect disability and multi-domain clinical impairment in patients with relapsing-remitting multiple sclerosis	10.1016/j.nicl.2022.103203
Z. Parray; M. H. Zargar; R. Asimi; W. R. Dar; A. Yaqoob; A. Raina; H. Ganie; M. Wani; Z. A. Shah	2022	Interleukin 32 gene promoter polymorphism: A genetic risk factor for multiple sclerosis in Kashmiri population	10.1016/j.gene.2022.146261
S. R. Nath; P. Grewal; T. Cho; Y. Mao-Draayer	2022	Familial multiple sclerosis in patients with Von Hippel-Lindau disease	10.1186/s12883-022-02604-6
F. Nabizadeh; M. Balabandian; M. R. Rostami; M. Owji; M. A. Sahraian; M. Bidadian; F. Ghadiri; N. Rezaeimanesh; A. N. Moghadasi	2022	Association of cognitive impairment and quality of life in patients with multiple sclerosis: A cross-sectional study	10.18502/cjn.v21i3.11106
K. Makowiecki; N. Stevens; C. L. Cullen; A. Zarghami; P. T. Nguyen; L. Johnson; J. Rodger; M. R. Hinder; M. Barnett; K. M. Young; B. V. Taylor	2022	Safety of low-intensity repetitive transcranial magneTic brAin stimUlation foR people living with mUltiple Sclerosis (TAURUS): study protocol for a randomised controlled trial	10.1186/s13063-022-06526-z
I. Koubiyr; C. Dulau-Metras; M. Deloire; J. Charré-Morin; A. Saubusse; B. Brochet; A. Ruet	2022	Amygdala network reorganization mediates the theory of mind performances in multiple sclerosis	10.1002/jnr.24986
E. M. Khedr; T. Desoky; A. Gamea; M. Y. Ezzeldin; A. F. Zaki	2022	Fatigue and brain atrophy in Egyptian patients with relapsing remitting multiple sclerosis	10.1016/j.msard.2022.103841
A. Kever; K. Buyukturkoglu; S. N. Levin; C. S. Riley; P. De Jager; V. M. Leavitt	2022	Associations of social network structure with cognition and amygdala volume in multiple sclerosis: An exploratory investigation	10.1177/13524585211018349
E. Kantorová; P. Hnilicová; W. Bogner; M. Grendár; J. Grossmann; S. Kováčová; E. Hečková; B. Strasser; D. Čierny; K. Zeleňák; E. Kurča	2022	Neurocognitive performance in relapsing-remitting multiple sclerosis patients is associated with metabolic abnormalities of the thalamus but not the hippocampus–GABA-edited 1H MRS study	10.1080/01616412.2021.1956282
P. Glasner; A. Sabisz; M. Chylińska; J. Komendziński; A. Wyszomirski; B. Karaszewski	2022	Retinal nerve fiber and ganglion cell complex layer thicknesses mirror brain atrophy in patients with relapsing-remitting multiple sclerosis	10.3233/RNN-211176
L. Gilio; D. Fresegna; A. Gentile; L. Guadalupi; K. Sanna; F. De Vito; S. Balletta; S. Caioli; F. R. Rizzo; A. Musella; E. Iezzi; A. Moscatelli; G. Galifi; R. Fantozzi; P. Bellantonio; R. Furlan; A. Finardi; V. Vanni; E. Dolcetti; A. Bruno; F. Buttari; G. Mandolesi; D. Centonze; M. Stampanoni Bassi	2022	Preventive exercise attenuates IL-2-driven mood disorders in multiple sclerosis	10.1016/j.nbd.2022.105817

L. Gilio; F. Buttari; L. Pavone; E. Iezzi; G. Galifi; E. Dolcetti; F. Azzolini; A. Bruno; A. Borrelli; M. Storto; R. Furlan; A. Finardi; T. Pekmezovic; J. Drulovic; G. Mandolesi; D. Fresegna; V. Vanni; D. Centonze; M. Stampanoni Bassi	2022	Fatigue in Multiple Sclerosis Is Associated with Reduced Expression of Interleukin-10 and Worse Prospective Disease Activity	10.3390/biomedicines10092058
M. Fleischer; H. Schuh; N. M. Bickmann; T. Hagenacker; K. Krüger; T. Skripuletz; M. Fiedler; C. Kleinschnitz; R. Pul; J. Skuljec	2022	Anti-EBNA1 IgG titre is not associated with fatigue in multiple sclerosis patients	10.5603/PJNNS.a2022.0043
M. Chylińska; B. Karaszewski; J. Komendziński; A. Wyszomirski; A. Sabisz; M. Halas; E. Szurowska	2022	Skeletonized mean diffusivity and neuropsychological performance in relapsing-remitting multiple sclerosis	10.1002/brb3.2591
A. Carotenuto; P. Valsasina; P. Preziosa; D. Mistri; M. Filippi; M. A. Rocca	2022	Monoaminergic network abnormalities: A marker for multiple sclerosis-related fatigue and depression	10.1136/jnnp-2022-330109
A. Alshehri; O. Al-iedani; J. Arm; N. Gholizadeh; T. Billiet; R. Lea; J. Lechner-Scott; S. Ramadan	2022	Neural diffusion tensor imaging metrics correlate with clinical measures in people with relapsing-remitting MS	10.1177/19714009211067400
O. Akmaz; A. Koskderelioglu; N. Eskut; B. Sahan; T. Kusbeci	2022	Restless legs syndrome in multiple sclerosis is related to retinal thinning	10.1016/j.pdpdt.2022.103169
E. A. Høgestøl; S. Ghezzo; G. O. Nygaard; T. Espeseth; P. Sowa; M. K. Beyer; H. F. Harbo; L. T. Westlye; H. E. Hulst; D. Alnæs	2022	Functional connectivity in multiple sclerosis modelled as connectome stability: A 5-year follow-up study	10.1177/13524585211030212
R. Zhou; H. Li; H. Yang; F. Jiang; H. Cai; J. Li; S. Chen; L. Fang; J. Yin; Q. Zeng	2022	Serological markers exploration and real-word effectiveness and safety of teriflunomide in south Chinese patients with multiple sclerosis	10.1016/j.msard.2021.103446
B. Xiang; J. Wen; R. E. Schmidt; A. L. Sukstanskii; D. Mamah; D. A. Yablonskiy; A. H. Cross	2022	Evaluating brain damage in multiple sclerosis with simultaneous multi-angular-relaxometry of tissue	10.1002/acn3.51621
L. Wu; Z. Zhang; X. Liang; Y. Zhu; H. Gong; F. Zhou	2022	Evaluation of brain glymphatic system function in patients with multiple sclerosis based on diffusion tensor imaging	10.3760/cma.j.cn115354-20220803-00544
M. Wnuk; L. Drabik; M. Marona; J. Szaleniec; A. Bryll; P. Karcz; J. Kolasinska; M. Kolasinska; M. Ziekiewicz; J. Skladzien; T. Popiela; A. Slowik	2022	Olfactory Dysfunction in Patients With Relapsing-Remitting Multiple Sclerosis Treated With Disease-Modifying Therapies	10.1177/0145561320973777
C. A. F. Román; G. R. Wylie; J. DeLuca; B. Yao	2022	Associations of White Matter and Basal Ganglia Microstructure to Cognitive Fatigue Rate in Multiple Sclerosis	10.3389/fneur.2022.911012

L. Pontieri; M. Blinkenberg; S. Bramow; V. Papp; P. V. Rasmussen; M. Kant; J. Schäfer; H. K. Mathiesen; M. B. Jensen; G. Sirakov; J. M. Berg; T. I. Kopp; H. Joensen; F. Sellebjerg; M. Magyari	2022	Ocrelizumab treatment in multiple sclerosis: A Danish population-based cohort study	10.1111/ene.15142
T. Odintsova; O. Kopchak	2022	Predicting cognitive impairment and psycho-emotional disorders in Multiple Sclerosis patients according to MRI findings	10.37897/RJN.2022.4.11
M. Margoni; P. Preziosa; P. Tortorella; M. Filippi; M. A. Rocca	2022	Does Ocrelizumab Limit Multiple Sclerosis Progression? Current Evidence from Clinical, MRI, and Fluid Biomarkers	10.1007/s13311-022-01252-5
D. K. Karathanasis; A. Rapti; A. Nezos; C. Skarlis; C. Kilidireas; C. P. Mavragani; M. E. Evangelopoulos	2022	Differentiating central nervous system demyelinating disorders: The role of clinical, laboratory, imaging characteristics and peripheral blood type I interferon activity	10.3389/fphar.2022.898049
S. A. Glasmacher; P. K. Kearns; Z. Hassan; P. Connick; S. Tauber; K. Reetz; P. Foley; S. Chandran; M. S. C. Future	2022	The influence of disease-modifying therapy on hidden disability burden in people with newly diagnosed relapsing-remitting multiple sclerosis	10.1016/j.msard.2022.103837

J. F. Foley; G. Defer; L. Z. Ryerson; J. A. Cohen; D. L. Arnold; H. Butzkueven; G. Cutter; G. Giovannoni; J. Killestein; H. Wiendl; K. Smirnakis; S. Xiao; G. Kong; R. Kuhelj; N. Campbell; A. van der Walt; C. Dwyer; K. Buzzard; J. Spies; J. Parratt; V. van Pesch; B. Willekens; G. Perrotta; E. Bartholomé; F. Grand'Maison; F. Jacques; P. Giacomini; R. Vosoughi; J. M. Girard; J. de Seze; C. Lebrun Frenay; A. Ruet; D. A. Laplaud; G. Reifschneider; B. Wagner; S. Rauer; R. Pul; M. Seipelt; A. Berthele; L. Klotz; B. A. Kallmann; F. Paul; A. Achiron; G. Lus; D. Centonze; F. Patti; L. Grimaldi; R. Hupperts; S. Frequin; J. Fermont; S. E. Madueno; A. M. Alonso Torres; L. Costa-Frossard França; J. E. Meca-Lallana; L. B. Ruiz; O. Pearson; D. Rog; N. Evangelou; A. Ismail; E. Lathi; E. Fox; T. Leist; J. Sloane; G. Wu; B. Khatri; B. Steingo; B. Thrower; M. Gudesblatt; J. Calkwood; D. Bandari; J. Scagnelli; C. Laganke; D. Robertson; L. Kipp; M. Belkin; S. Cohan; L. Goldstick; A. Courtney; W. Vargas; A. Sylvester; J. Srinivasan; M. Kannan; M. Picone; J. English; S. Napoli; R. Balabanov; I. Zaydan; J. Nicholas; J. Kaplan; F. Lublin; E. Riser; T. Miller; E. Alvarez; S. Wray; J. Gross; S. Pawate; C. Hersh; L. McCarthy; H. Crayton; J. Graves	2022	Comparison of switching to 6-week dosing of natalizumab versus continuing with 4-week dosing in patients with relapsing-remitting multiple sclerosis (NOVA): a randomised, controlled, open-label, phase 3b trial	10.1016/S1474-4422(22)00143-0
N. M. El Garhy; M. M. El Toukhy; M. M. Fatouh	2022	MR volumetry in detection of brain atrophic changes in MS patients and its implication on disease prognosis: retrospective study	10.1186/s43055-022-00726-y
S. Diker	2022	Fingolimod in Multiple Sclerosis and Familial Mediterranean Fever Coexistence	10.4103/0028-3886.338713
B. A. C. Cree; K. W. Selmaj; L. Steinman; G. Comi; A. Bar-Or; D. L. Arnold; H. P. Hartung; X. Montalbán; E. K. Havrdová; J. K. Sheffield; N. Minton; C. Y. Cheng; D. Silva; L. Kappos; J. A. Cohen	2022	Long-term safety and efficacy of ozanimod in relapsing multiple sclerosis: Up to 5 years of follow-up in the DAYBREAK open-label extension trial	10.1177/13524585221102584

B. A. C. Cree; D. L. Arnold; R. J. Fox; R. Gold; P. Vermersch; R. H. B. Benedict; A. Bar-Or; D. Piani-Meier; N. Rouyrre; S. Ritter; A. Kilaru; G. Karlsson; G. Giovannoni; L. Kappos	2022	Long-term efficacy and safety of siponimod in patients with secondary progressive multiple sclerosis: Analysis of EXPAND core and extension data up to >5 years	10.1177/13524585221083194
L. Chu; A. Balusha; C. Casserly; W. Berger; S. A. Morrow	2022	Relationship between lymphopenia and disease activity in persons with multiple sclerosis treated with dimethyl fumarate	10.1016/j.msard.2021.103384
O. Al-iedani; R. Lea; K. Ribbons; S. Ramadan; J. Lechner-Scott	2022	Neurometabolic changes in multiple sclerosis: Fingolimod versus beta interferon or glatiramer acetate therapy	10.1111/jon.13032
S. Ziccardi; F. B. Pizzini; M. Guandalini; A. Tamanti; C. Cristofori; M. Calabrese	2023	Making Visible the Invisible: Automatically Measured Global and Regional Brain Volume Is Associated with Cognitive Impairment and Fatigue in Multiple Sclerosis	10.3390/bioengineering10010041
B. Wagner; C. L. Härig; B. Walter; J. Sommer; G. Sammer; M. Berghoff	2023	Is There Reduced Hemodynamic Brain Activation in Multiple Sclerosis Even with Undisturbed Cognition?	10.3390/ijms24010112
K. Siddiqui; R. W. Browne; R. H. B. Benedict; D. Jakimovski; B. Weinstock-Guttman; R. Zivadinov; M. Ramanathan	2023	Cholesterol pathway biomarkers are associated with neuropsychological measures in multiple sclerosis	10.1016/j.msard.2022.104374
I. Rosenstein; M. Axelsson; L. Novakova; S. Rasch; K. Blennow; H. Zetterberg; J. Lycke	2023	High levels of kappa free light chain synthesis predict cognitive decline in relapsing-remitting multiple sclerosis	10.3389/fimmu.2023.1106028
A. Damasceno; L. R. Pimentel-Silva; B. P. Damasceno; F. Cendes	2023	Are we ready to define cognitive worsening in MS? How different cutoffs detect future cognitive worsening after six years of follow-up	10.1016/j.msard.2022.104402
Y. T. Chang; P. K. A. Kearns; A. Carson; D. C. Gillespie; R. Meijboom; A. Kampaite; M. D. C. Valdés Hernández; C. Weaver; A. Stenson; N. MacDougall; J. O'Riordan; M. A. Macleod; F. J. Carod-Artal; P. Connick; A. D. Waldman; S. Chandran; P. Foley	2023	Network analysis characterizes key associations between subjective fatigue and specific depressive symptoms in early relapsing-remitting multiple sclerosis	10.1016/j.msard.2022.104429
A. Bellinvia; E. Portaccio; M. P. Amato	2023	Current advances in the pharmacological prevention and management of cognitive dysfunction in multiple sclerosis	10.1080/14656566.2022.2161882
Q. Zhu; Z. Yan; Z. Shi; D. Luo; S. Ding; X. Chen; Y. Li	2023	Increased cortical lesion load contributed to pathological changes beyond focal lesion in cortical gray matter of multiple sclerosis: A diffusion kurtosis imaging analysis	10.1093/cercor/bhad332
