# Supplementary material for: Brain connectivity changes underlying depression and fatigue in relapsing-remitting multiple sclerosis: A systematic review
Source: PLoS One. 2024 Mar 29;19(3):e0299634. doi: 10.1371/journal.pone.0299634 (PMC10980255; doi:10.1371/journal.pone.0299634)
Supplement: Attachment — Submitted filename: Response_To_Reviewers.pdf [file pone.0299634.s015.pdf]

We thank the reviewers for their helpful comments and queries, which we have addressed on a point-by-point basis below, and referenced changes to the manuscript in response to these.

**Journal Requirements:**

Please review your reference list to ensure that it is complete and correct. If you have cited papers that have been retracted, please include the rationale for doing so in the manuscript text, or remove these references and replace them with relevant current references. Any changes to the reference list should be mentioned in the rebuttal letter that accompanies your revised manuscript. If you need to cite a retracted article, indicate the article's retracted status in the References list and also include a citation and full reference for the retraction notice.

We have carefully reviewed the reference list and found no inclusion of retracted papers. We did, however, observe several required modifications. We apologise for previously having overlooked these errors in our reference list. We have now made these modifications to the reference list where needed:

1. Cao et al. - replaced correction with the original corrected paper.

*Cao Y, Diao W, Tian F, Zhang F, He L, Long X, et al. Gray Matter Atrophy in the Cortico-Striatal-Thalamic Network and Sensorimotor Network in Relapsing–Remitting and Primary Progressive Multiple Sclerosis. Neuropsychology Review. 2021;31(4):703-20. doi: 10.1007/s11065-021-09479-3.*

2. Golde et al. – corrected duplicate.

*Golde S, Heine J, Pöttgen J, Mantwill M, Lau S, Wingenfeld K, et al. Distinct Functional Connectivity Signatures of Impaired Social Cognition in Multiple Sclerosis. Frontiers in Neurology. 2020;11. doi: 10.3389/fneur.2020.00507.*

3. Baker and Tadi – edited citation from 'book' to 'website'.

*Banker L, Tadi P. Neuroanatomy, Precentral Gyrus. In: StatPearls [Internet]. StatPearls Publishing, Treasure Island (FL); 2023 [updated 2023 Jul 24]. Available from: https://www.ncbi.nlm.nih.gov/books/NBK544218/.*

4. Ghandili – edited citation from 'book' to 'website'.

*Ghandili M, Munakomi S. Neuroanatomy, Putamen. In: StatPearls [Internet]. StatPearls Publishing, Treasure Island (FL); 2023 [updated 2023 Jan 30]. Available from: https://www.ncbi.nlm.nih.gov/books/NBK542170/.*

5. Valentine et al. – edited doi.

*Valentine TR, Alschuler KN, Ehde DM, Kratz AL. Prevalence, co-occurrence, and trajectories of pain, fatigue, depression, and anxiety in the year following multiple sclerosis diagnosis. Multiple Sclerosis Journal. 2022;28(4):620-31. doi: 10.1177/13524585211023352. PubMed PMID: 34132141.*

**Reviewers' comments:**

Reviewer #3:

1. I miss important information on heat sensitivity and MS in the back ground - this is a typical feature of MS and is intertwined with fatigue in particular. The potential of heat sensitivity to increase fatigue as well as other core symptoms is generally overlooked Consequently, cryotherapy against fatigue should be mentioned more clearly, for example in the introductory text on therapies used for fatigue. Suitable references by Flensner et al, especially https://doi-org.ezproxy.its.uu.se/10.1186/1471-2377-11-27

   We agree with the reviewer and have now revised the introduction and discussion to include heat sensitivity as contributing factor to fatigue in MS and cryotherapy as potential treatment for it.

   Introduction:

   *"Fatigue can appear spontaneously, or be brought on by a combination of internal or external factors, such as mental or physical activity, heat sensitivity, humidity, acute infection, and food ingestion [7, 36]. Commonly suggested primary mechanisms of fatigue in MS involve the immune system or damage to the CNS, such as inflammatory processes (e.g. cytokines), endocrine dysregulation, axonal loss, demyelination, as well as functional connectivity changes. [9, 37, 38]. This review will focus on structural damage of the CNS in the white (WM) and grey matter GM), specifically changes in structural and functional brain connectivity, as potential underlying mechanism of fatigue in pwRRMS."*

   *"Additionally, antidepressants, cognitive behavioural therapy [6] and cryotherapy [55] have had some success in reducing both depression and fatigue symptomatology in MS. Given the limited treatment success, underlying CNS changes of fatigue and depression in MS need to be elucidated, which may aid development of more effective targeted treatments for both symptoms in MS."*

   Discussion:

   *"It may also indicate a possibility for shared treatments such as cognitive behavioural therapy, cryotherapy and balance and/or multicomponent exercise, both of which show promising results in CFS [248-250]."*

2. I also miss one paper on fMRI and fatigue that seems to fit with the inclusion criteria of the authors, i.e. dividing subtypes and reporting fatigue, available on Pubmed: Engstrom et al https://doi-org.ezproxy.its.uu.se/10.1002/brb3.181.

   We thank the reviewer for suggesting the above paper. We have reviewed the article by Engstrom et al. and noticed the study included 15 people with MS. This is below our set inclusion criteria of $n \geq 20$. We realised we did not include this criterion in the abstract, which may have caused confusion. We have now added it to the abstract accordingly.

   *"Searched databases were PubMed, Web-of-Science and Scopus. Inclusion criteria were: studied participants with RRMS (n ≥ 20; ≥ 18 years old) and differentiated between MS subtypes; published between 2001-01-01 and 2023-01-18; used fatigue and depression assessments validated for MS; included brain structural, functional magnetic resonance imaging (fMRI) or*

*diffusion MRI (dMRI). Sixty studies met the criteria: 18 dMRI (15 fatigue, 5 depression) and 22 fMRI (20 fatigue, 5 depression) studies."*

3. This work is congruent with the works by Filippi, but also touches upon potential mechanisms of fatigue, which could be expanded a bit in the text.

   We agree with the reviewer and have revised the introduction to expand on the potential primary mechanisms of fatigue in MS.

   *"Fatigue can appear spontaneously, or be brought on by a combination of internal or external factors, such as mental or physical activity, heat sensitivity, humidity, acute infection, and food ingestion [7, 36]. Commonly suggested primary mechanisms of fatigue in MS involve the immune system or damage to the CNS, such as inflammatory processes (e.g., cytokines), endocrine dysregulation, axonal loss, demyelination, as well as functional connectivity changes [9, 37, 38]. This review will focus on structural damage of the CNS in the white (WM) and grey matter (GM), specifically changes in structural and functional brain connectivity, as potential underlying mechanism of fatigue in pwRRMS."*

4. Finally, you may add the first collection of articles before selection using your criteria, appendix or supplement.

   We thank the reviewer for this excellent suggestion and have now created a list of all the articles included in our initial database search, with duplicates removed, and added this as supporting information file S11.

**<u>Further modifications</u>**

Due to the reviewer comments, we decided that the abstract's conclusion required further modifications to improve clarity. We have now revised the abstract accordingly and apologise for these further changes.

*"Qualitative analysis suggests structural and functional connectivity changes, possibly due to axonal and/or myelin loss, in the cortico-thalamic-basal-ganglial and cortico-limbic network may underlie fatigue and depression in pwRRMS, respectively, but the overall results were inconclusive, possibly explained by heterogeneity and limited number of studies. This highlights the need for further studies including advanced MRI to detect more subtle brain changes in association with depression and fatigue. Future studies using optimised imaging protocols and validated depression and fatigue measures are required to clarify the substrates underlying these symptoms in pwRRMS."*
