# Supplementary material for: Brain connectivity changes underlying depression and fatigue in relapsing-remitting multiple sclerosis: A systematic review
Source: PLoS One. 2024 Mar 29;19(3):e0299634. doi: 10.1371/journal.pone.0299634 (PMC10980255; doi:10.1371/journal.pone.0299634)
Supplement: Attachment — Submitted filename: review.docx [file pone.0299634.s013.docx]

This systematic review used the PRISMA approach to review studies in the MS field, with a focus on depression and fatigue. The papers started with a comprehensive introduction regarding MS symptoms and MRI types of data, followed by the methodology section which referred to the keywords for searching. The results are presented in tables by referring to the papers included in the systematic review. Then, the authors discussed brain networks regarding fatigue and depression in MS patients. The PRISMA approach clearly shows the search process. The brain network reviews for depression and fatigue were a strength of the paper. The limitations of the studies included in the paper serve as a valuable resource for researchers to identify research gaps, which is one of the key objectives of a literature review. It was a nice job!

However, there are a few points that could be modified and added:

1) It is unclear why depression and fatigue were reviewed together and how they are related. The authors could have provided a rationale for why other MS cognitive symptoms were not included.
2) The sentence "This is supported the observation that depression is more prevalent in MS than in other neurodegenerative disorders" could be improved by providing a reference that compares MS to other neurodegenerative disorders in this regard. With this references, It is not proven that depression is more prevalent in MS than in ALS, for instance.
3) The authors utilized three top databases in their search for relevant literature; however, it should be noted that Google Scholar results are also a comprehensive resource when conducting a systematic review. (Please check this paper if it is eligible to be included , found from first page of search on Google scholar with the inclusion and exclusion criteria.)

- Carotenuto, A., Valsasina, P., Preziosa, P., Mistri, D., Filippi, M., & Rocca, M. A. (2023). Monoaminergic network abnormalities: a marker for multiple sclerosis-related fatigue and depression. *Journal of Neurology, Neurosurgery & Psychiatry*, *94*(2), 94-101.

4) The research questions are not stated in the methodology section. The authors should perfectly illustrate the aim of the study.
5) The use of "PwRRMS" as a short form is not common and could be confusing in the paper.
6) Table 1 does not need to be in the main text and could be summarized in a few lines.
7) In Table 4, the "Brain Region" column does not necessarily represent the region. For example, in cells 1-3 I, it shows the condition rather than regions.
8) The authors presented the overlapping of depression and fatigue in the papers included in the review using three long tables. It would be helpful for the readers to better understand the relationship between depression and fatigue in MS patients and to identify potential research gaps. In the text, it would be beneficial to provide a brief explanation of why it is important to study the co-occurrence of these symptoms.
9) The authors discussed brain networks perfectly and in a well-structured manner. The same could be done for disease progression regarding depression and fatigue in MS patients.
